# Harnessing Substituted 4-Chlorothieno[2,3-*b*]pyridine as a New Cap for Potent and Selective Antiproliferative HDAC Inhibitors

**DOI:** 10.3390/ph19030442

**Published:** 2026-03-09

**Authors:** Mostafa M. Badran, Berkay Beyri, Hiroshi Tateishi, Kazunori Shimagaki, Akiko Nakata, Akihiro Ito, Nao Nishimura, Samar H. Abbas, Mohamed Abdel-Aziz, Masami Otsuka, Minoru Yoshida, Mikako Fujita, Stefan Bräse, Mohamed O. Radwan

**Affiliations:** 1 Department of Medicinal Chemistry, Faculty of Pharmacy, Qena University (Formerly South Valley University), Qena 83523, Egypt; mostbadran@svu.edu.eg; 2 Medicinal and Biological Chemistry Science Farm Joint Research Laboratory, Faculty of Life Sciences, Kumamoto University, 5-1 Oe-honmachi, Chuo-ku, Kumamoto 862-0973, Japan; 243y2051@st.kumamoto-u.ac.jp (B.B.); htateishi@kumamoto-u.ac.jp (H.T.); kshimagaki@kumamoto-u.ac.jp (K.S.); motsuka@gpo.kumamoto-u.ac.jp (M.O.); mfujita@kumamoto-u.ac.jp (M.F.); 3 Drug Discovery Seeds Development Unit, RIKEN Center for Sustainable Resource Science, 2-1 Hirosawa, Wako, Saitama 351-0198, Japan; anakata@riken.jp (A.N.); aito@toyaku.ac.jp (A.I.); yoshidam@riken.jp (M.Y.); 4 Laboratory of Cell Signaling, School of Life Sciences, Tokyo University of Pharmacy and Life Sciences, Hachioji, Tokyo 192-0392, Japan; 5 Department of Hematology, Rheumatology and Infectious Diseases, Kumamoto University Hospital, 1-1-1 Honjo, Chuo-ku, Kumamoto 860-8556, Japan; naonishimura.kmuv@gmail.com; 6 Medicinal Chemistry Department, Faculty of Pharmacy, Minia University, Minia 61519, Egypt; samar_hafez@mu.edu.eg (S.H.A.); abulnil@mu.edu.eg (M.A.-A.); 7 Medicinal Chemistry Department, Faculty of Pharmacy, Minia National University, New Minia 61768, Egypt; 8 Department of Drug Discovery, Science Farm Ltd., 1-7-30 Kuhonji, Chuo-ku, Kumamoto 862-0976, Japan; 9 Office of University Professors, The University of Tokyo, 1-1-1 Yayoi, Bunkyo-ku, Tokyo 113-8657, Japan; 10 Department of Molecular Biology and Genetics, Burdur Mehmet Akif, Ersoy University, Degirmenler Mahallesi-Istiklal Yerleskesi, Yakakoy, Merkez, Burdur 15200, Turkey; 11 Institute of Biological and Chemical Systems—Functional Molecular Systems (IBCS-FMS), Karlsruhe Institute of Technology (KIT), Kaisersrasse 12, 76131 Karlsruhe, Germany; 12 UF Genetics Institute, University of Florida, Gainesville, FL 32610, USA; 13 Department of Cellular and Systems Pharmacology, College of Pharmacy, University of Florida, Gainesville, FL 32610, USA; 14 Department of Medicinal Chemistry, College of Pharmacy, University of Florida, Gainesville, FL 32610, USA

**Keywords:** HDAC inhibitors, 4-chlorothieno[2,3-*b*]pyridine, [2,3-*b*]pyridine, anticancer, Vilsmier Haack, hydroxamic acid, RPMI-8226 multiple myeloma

## Abstract

**Background:** Inhibition of histone deacetylase is a highly sought-after objective in the fight against cancer. Thus, the development of innovative HDAC inhibitors with significantly higher potency than SAHA against specific cancer cell types represents complex and demanding work. **Method:** The utilization of the underexplored and privileged scaffold 4-chlorothieno[2,3-*b*]pyridine as a cap tethering diverse aliphatic and aromatic linkers, followed by the screening of both cellular and enzymatic activities, is undertaken in this study. **Results:** Compounds **7a** and **9a** demonstrated impressive mean GI_50_ values of 2.15 µM and 1.89 µM, respectively. Both compounds reduced caspase-3 levels in RPMI-8226 cells, suggesting induction of apoptosis. Compound **7a** showed remarkable IC_50_ values of 0.37 µM, 0.58 µM, and 0.70 µM against HDACs 1, 4, and 6, respectively, consistent with the cellular assay. Additionally, compound **7a** exhibited a selectivity index of 11 for RPMI-8226 cells over PBMCs, reflecting its high selectivity and potential safety. Moreover, ADMET prediction tools indicated that compounds **7a** and **9b** may have more favorable pharmacokinetic properties than the gold-standard HDAC inhibitor, SAHA. **Conclusions:** Further study and exploration of the derivatives of compounds **7a** and **9a** can lead to further advancement in the development of potent HDAC inhibitor anticancer drugs.

## 1. Introduction

Histone deacetylases (HDACs) are a family of conserved enzymes that catalyze the removal of acetyl groups from lysine residues of the histone protein tail. The enzymes are involved in many cellular functions, including gene transcription, protein stability, protein interactions, and subcellular localization [1,2].

HDACs comprise four main classes based on their structure and specificity [1,2,3]:

Class I includes HDAC1, HDAC2, HDAC3, and HDAC8. Class IIa consists of HDAC4, HDAC5, HDAC6, and HDAC11. Class IIb includes HDAC4, HDAC5, HDAC6, HDAC7, HDAC9, and HDAC10. Class III consists of seven isozymes, called Sirtuins (Sirtuins 1–7), and class IV includes HDAC11.

The classical family of HDACs, which includes classes I, IIa, IIb, and IV, relies on Zn^+2^ as a cofactor for their deacetylation process. On the other hand, Sirtuins, which belong to the class III HDACs, are NAD+-dependent [4,5].

Aberrant gene expression of HDACs is associated with a vast array of malignancies, such as squamous cell lung carcinoma, pancreatic cancer, hepatocellular carcinoma, myeloid leukemia, and prostate cancers [6]. In addition to their role in tumorigenesis, HDACs also play a role in inflammatory diseases, including neuromuscular degeneration, arthritis, and gastrointestinal diseases [7,8,9].

**Figure 1:**
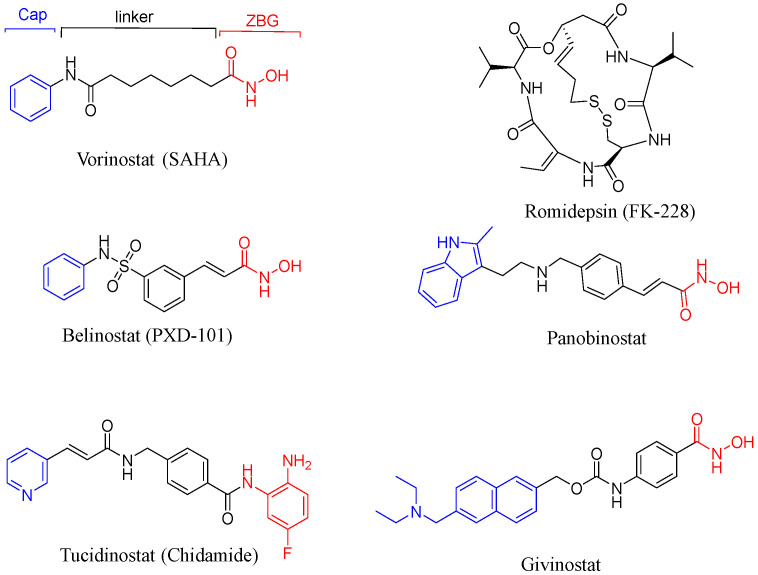
List of approved HDAC inhibitors.

For over three decades, the natural product trichostatin A has been extensively studied for its inhibitory effects on mammalian HDACs. Inhibition of HDACs using small molecules has proven successful in suppressing various types of cancer. Currently, five approved drugs for the treatment of hematologic malignancies use this approach: vorinostat (SAHA), belinostat (PXD-101), romidepsin (FK-228), Panobinostat (LBH589), and Tucidinostat (Chidamide) (Figure 1) [10]. Recently, givinostat was approved for the treatment of Duchenne muscular dystrophy in patients over 6 years of age [11].

The HDAC inhibitor pharmacophore consists of three motifs [12]:

1. A cap of a hydrophobic aromatic or heteroaromatic nucleus group that binds the HDAC surface.
2. A zinc binding group (ZBG) of hydroxamic, thiol, benzamide, hydrazide, or other groups that chelate the Zn^+2^ ion.
3. An aliphatic, aromatic, or heteroaromatic spacer that connects the previous motifs and forms a hydrophobic interaction with the hydrophobic tunnel.

Thieno[2,3-*b*]pyridine is a heterocyclic compound that garnered attention in recent years due to its biological importance. This scaffold was used to produce a vast array of bioactive compounds with antiepileptic [13], antiproliferative [14,15], anabolic [16], and anti-inflammatory effects [17]. Despite the promising biological potential of the thieno[2,3-b]pyridine ring, its medicinal chemistry applications are yet to be sufficiently explored. For example, 4-chlorothieno[2,3-*b*]pyridine derivatives were efficiently synthesized by Abdelwahab et al., but their biological activities were not explored [18].

**Figure 2:**
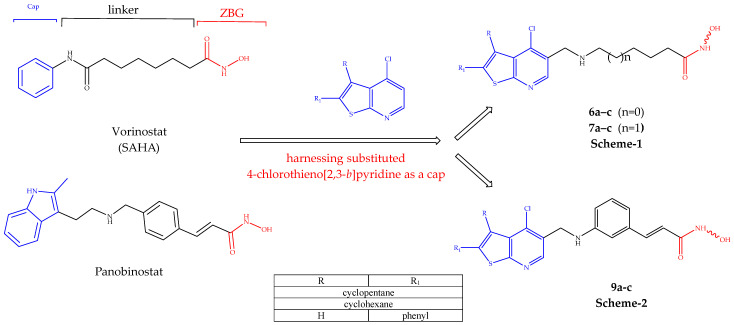
Rational design of compounds **6a**–**c** and **7a**–**c** (Scheme 1) and compounds **9a**–**e** (Scheme 2).

In an attempt to continue targeting the epigenetic machinery with new chemical entities [19,20,21], herein, the design and synthesis of a novel series of HDAC inhibitors are presented by incorporating 4-chlotothieno[2,3-b]pyridine as a new cap structure, with variable flexible and restricted linkers. Indeed, some of these candidates showed enhanced HDAC activity and antiproliferative activity. The rational design is given in Figure 2.

**Scheme 1:**
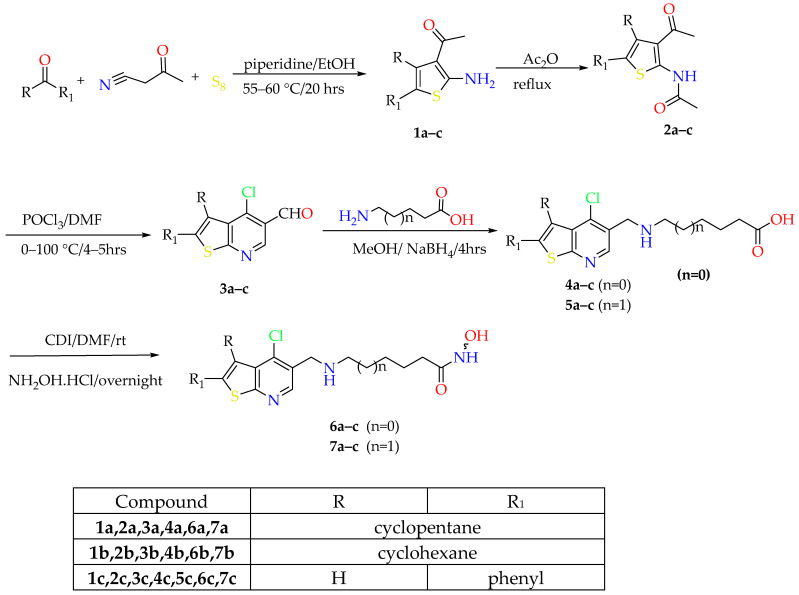
Synthetic approach of compounds **6a**–**c** and **7a**–**c**.

**Scheme 2:**
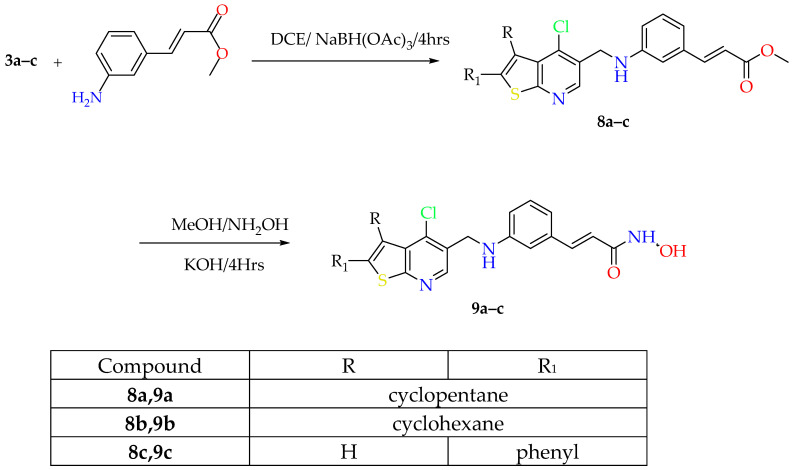
Synthetic approach of compounds **9a**–**c**.

## 2. Results and Discussion

### 2.1. Chemistry

The key starting intermediates **1a**–**c** were synthesized in good yield via the Gewald multicomponent reaction. To acquire formylated derivatives of 4–chlorothieno[2,3-*b*]pyridine instead of 4-chlorothieno[2,3-*b*]pyridine itself, it was necessary to perform acetylation on the thiophene derivatives **1a**–**c** using Ac_2_O and subsequently react them with 12 equivalents of Vilsmeier–Haack reagent added as one portion at a temperature not exceeding 65 °C [18]. The synthesized aldehyde derivatives **3a**–**c** underwent reductive amination with methyl (*E*)-3-aminocinnamate utilizing NaBH(OAc)_3_ in 1,2–dichloroethane (DCE), affording the ester intermediates **8a**–**e**. Due to the limited solubility of 6-aminohexanoic acid and 7-amino heptanoic acid in DCE, the synthesis of intermediates **4a**–**c** and **5a**–**c** was carried out in MeOH, followed by reduction using NaBH_4_. Synthesis of the final hydroxamic acid derivatives **6a**–**e** and **7a**–**c** was achieved by activation of acids **4a**–**c** and **5a**–**c** using *N,N′*- carbonyldiimidazole for 4 h, followed by addition of hydroxylamine hydrochloride (Scheme 1). Esters **8a**–**c** were reacted with NH_2_OH solution under high pH conditions using 10 equivalents of KOH to give rise to the remaining hydroxamic acid compounds **9a**–**c** (Scheme 1).

### 2.2. NCI-60 Screening of Anticancer Activity

All novel final compounds were selected by the National Cancer Institute (NCI) according to the protocol of the Drug Evaluation Branch of the National Cancer Institute, Bethesda, USA, for an in vitro one-dose anticancer assay. An anticancer assay was performed on all NCI-60 cell lines derived from nine tumor subpanels, including leukemia, melanoma, and lung, colon, CNS, ovarian, renal, prostate, and breast cancer.

#### 2.2.1. One-Dose Screening

The selected compounds were first tested at a single concentration of 10 µM. The activity was expressed as growth inhibition percentage (GI%), outlined in Table 1. Compounds **6a** and **6b**, which have shorter aliphatic linkers, generally showed low GI% values. However, compound 6a showed moderate activity against the MDA-MB-468 breast cancer cell line, with an inhibition rate of 40.42%. A remarkable increase in the inhibition activity was noticed with the longer linker in compounds **7a**–**c**. Compound **7a** demonstrated significant potency against all 59 cell lines, ranging from moderate to high levels, with an inhibition activity percentage of 42.55 to 169.23%. Compounds **7b** and **7c** showed inhibition activity with ranges of 16.77–129.98% and 21.80–111.86%, respectively. Ultimately, the cinnamic acid derivatives recorded significant potential antiproliferative activity, particularly compounds **9a** and **9b**. Notably, compound **9a** exhibited potent antiproliferative effects across all 59 cell lines tested, yielding exceptional results, with GI% ranging from 72.46 to 189.06%. The remaining compounds **9b** and **9c** exhibited GI% ranges of 45.74–191.11% and 11.55–104.52%, respectively.

#### 2.2.2. Five-Dose Screening

Compounds **6c**, **7a**,**b**, and **9a**,**b** displayed considerable cytotoxicity against different cancer cell lines in the preliminary NCI one-dose assay, so they were selected for advanced five-dose testing against the full panel of 58–60 human tumor cell lines. The outcomes were used to create log-concentration versus % growth inhibition curves, and three response parameters (GI_50_, TGI, and LC_50_) were calculated for each cell line. The GI_50_ value (growth inhibitory activity) corresponds to the concentration of the compound causing a 50% decrease in net cell growth, the TGI value (cytostatic activity) is the concentration of the compound resulting in total growth inhibition (TGI) and the LC_50_ value (cytotoxic activity) is the concentration of the compound causing net 50% loss of initial cells at the end of the incubation period of 48 h. Additionally, we retrieved the NCI screening results of SAHA as a reference for comparison (**NSC 759852**). All five-dose assay results are shown in Table 2 and the Supplementary Materials (Tables S1–S5).

Screening results for the selected compounds showed GI50 values in the submicromolar to single-digit micromolar range against most cell lines. Compound **6c** showed potent activity against all cancer cell lines, especially leukemia cancer cell lines, with GI_50_ values ranging from 1.38 µM to 2.03 µM, while it showed a TGI value of more than 65 µM against SR leukemia cell lines. The TGI values against the other cell lines were recorded to range from 3.48 µM to 18.4 µM. The LC_50_ of compound **6c** ranged from 25.2 µM to more than 65 µM. The potent compound **7a** demonstrated high antiproliferative activity against leukemia cell lines CCRF-CEM and SR, with submicomolar GI50 values of 0.97 µM and 9.56 µM, respectively. In addition, compound **7a** possessed a TGI range of 3.73 µM to more than 100 µM. Notably, it showed potent activity against RPMI-8226 leukemia, exceeding that of SAHA. Compound **7b** showed potential antiproliferative activity, with GI50 values ranging from 1.05 µM to 8.32 µM. Also, compound **7b** showed a TGI range of 3.54 µM to 29.3 µM. The LC_50_ range was 23.8 to >100 µM. On the other hand, compounds **9a** and **9b**, containing a cinnamic acid linker, exhibited potent antiproliferative activity against all cancer cell lines, with submicromolar activity against CCRF-CEM and SR leukemia cell lines, as well as HCT-15 colon cancer cell lines. Compound **9a** demonstrated values of 0.61 µM, 0.67 µM, and 0.55 µM, respectively, while compound 9b showed values of 0.78 µM, 0.82 µM, and 0.69 µM, respectively. Compound **9a** had the lowest mean GI50 across all 60 cell lines, at 1.88 µM. Interestingly, compounds **9a**,**b** showed higher potency than SAHA against the RPMI-8226 leukemia cell line, the NCI-H226 NCI-H322M non-small lung cancer cell lines, the SF-268, SF-539, and SNB-19 CNS cell lines, the IGROV1 and OVCAR-4 ovarian cancer cell lines, the 786-0 renal cancer cell line, and the MDA-MB-231/ATCC breast cancer cell line, while compound **9a** showed higher antiproliferative activity than SAHA against the RPMI-8226 leukemia cell line, the NCI-H23 NSCL cancer cell line, the OVCAR-3 ovarian cancer cell line, and the SN12C renal cancer cell line.

### 2.3. Antiproliferative Assay of Compound ***7a*** on Peripheral Blood Mononuclear Cells (PBMCs)

To investigate the selectivity of compound **7a** on the RPMI-8226 cell line over PBMCs, the cytotoxic effect of compound **7a** on PBMCs was examined using an MTT assay. The IC_50_ of compound **7a** against PBMCs was 15.03 µM, denoting a selectivity index around 11 and implying a favorable safety profile.

### 2.4. Evaluation of Apoptotic Gene Expression

**Figure 3:**
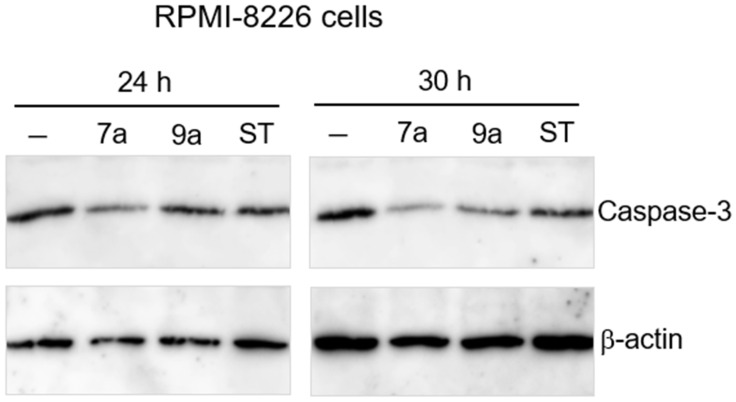
Western blot caspase-3 at various time points in the presence of 10 µM of compounds **7a** and **9a**, and 0.1 µM of staurosporine (ST) as a reference.

Compounds **7a** and **9a**, which exhibited the highest antiproliferative effects against cancer cell lines, were investigated to determine their ability to induce apoptosis through the assessment of the levels of the key apoptosis enzyme caspase-3. As anticipated, compounds **7a** and **9a** reduced the amount of caspase-3 at 24 h and 30 h, suggesting that the cytotoxic effect can be attributed to the induction of apoptosis (Figure 3).

### 2.5. HDAC Inhibition Activity

Compounds **6a**–**c**, **7a** and **9b** were screened for their inhibitory activity against HDACs 1, 4, and 6, taking trichostatin A (TSA) as a positive control (Table 3) [22]. Most of the screened compounds exhibited single-digit micromolar IC_50_ values against the three isozymes. Compound **7a**, featuring five methylene linkers, displayed the highest HDAC inhibitory activity, showing all IC50 values in the submicromolar range—recording 0.37 µM, 0.580 µM, and 0.7 µM against HDACs 1, 4, and 6, respectively. The other prominent compound, **9b** with a cinnamic acid linker, showed IC_50_ values of 2.26, 0.49 and 1.83 µM against HDACs 1, 4, and 6, respectively. Compounds **6a**–**c** exhibited comparable potencies, with IC_50_ values in a single-digit micromolar range, except for compound **6c** IC_50_ against HDAC6. The HADC inhibitory results showed the optimum length for HDAC inhibitors for the synthesis series is either a hexamethylene or a cinnamic acid linker.

### 2.6. Docking Study

**Figure 4:**
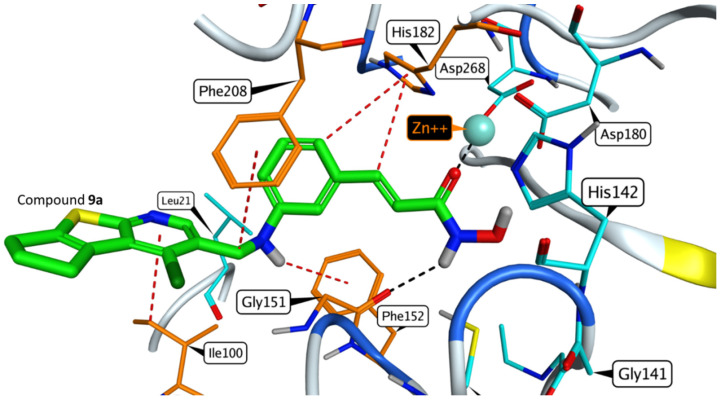
3D interaction of compound **9a** with the active site of HDLP (PDB: 1ZZ1).

An in silico docking study of the final compounds was performed with the Molecular Operating Environment 2019 (MOE). All final hydroxamic acid derivatives were docked to the active site of the HDLP protein (PDB: 1ZZ1). For compounds **6a**–**c** and **7a**–**c**, which contain an aliphatic linker with hydroxamic acid derivatives, SAHA was chosen as a reference compound. On the other hand, for compounds **9a**–**c**, which have a cinnamic acid linker, Panobinostat was selected as a reference compound. The tested compounds showed a relatively similar binding mode to SAHA. The SAHA carbonyl of the hydroxamic acid motif forms a metal bond with the Zn^+2^ ion of the enzyme. Moreover, hydroxamic acids NH and OH act as hydrogen-binding donors with Gly151 and Cys153, respectively. In the case of compound **6c**, the aliphatic chain linker forms a hydrophobic bond with His 182, while for the other compounds and SAHA, hydrophobic interactions occur with Phe208. The thienopyridine ring in the case of compound **7b** is precisely tailored to have a hydrophobic cap region that fits well. Additionally, it forms hydrogen bonding interactions between the sulfur (S) and nitrogen (N) atoms with Cys267. Furthermore, it engages in hydrophobic interactions with Phe208 for added potency. Regarding the binding of the potent compound **9a**, it exhibited a striking resemblance to Panobinostat. In addition to its hydroxamic function group binding to Zn and Gly151, the cinnamic acid linker in the molecule establishes multiple hydrophobic interactions with Phe28 and His182 residues. Moreover, the thienopyridine ring substitution results in an interaction with the amino acid residue Cys207. Interestingly, all the final compounds showed a docking score range of −8.97 to –9.73 kj/mol, surpassing both SAHA (−8.66 kj/mol) and Panobinostat (−8.68 kj/mol) (Figure 4 and Supplementary Materials Figures S1 and S2).

### 2.7. In Silico Physicochemical and Pharmacokinetic Prediction

To validate the enhanced physicochemical and pharmacokinetic properties of the synthesized compounds, the freely accessible SwissADME website provided by the Swiss Institute of Bioinformatics (http://www.swissadme.ch/index.php (accessed on 16 December 2023)) was employed. This allowed us to predict various physicochemical and pharmacokinetic parameters of the synthesized compounds and compare them with **SAHA** [23,24,25].

The BOILED-Egg method, a sturdy model developed to accurately forecast gastrointestinal absorption and brain accessibility of chosen compounds, utilizes lipophilicity (measured in WLOGP) and polarity (measured in TPSA) calculations.

Compounds **6b** and **7a** exhibit similar ADMET properties, leading to their placement in the same position within the BOILED_egg model. Consequently, the results for each compound are portrayed in separate diagrams alongside the remaining compounds.

**Figure 5:**
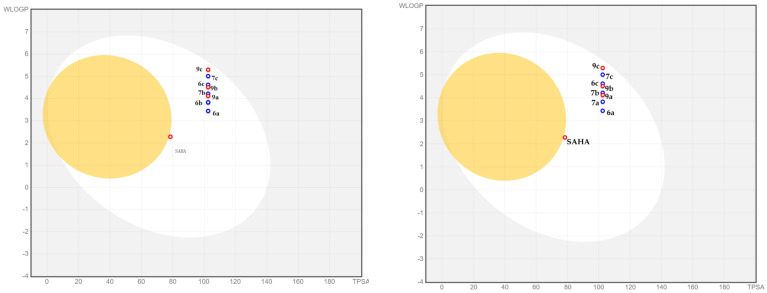
BOILED-Egg plots for SAHA and final compounds.

All the final compounds exhibited high GI absorption due to their balanced lipophilicity (WLOGP = 3.44–5.3) and polarity (TPSA = 102.49 Å^2^). Importantly, none of these compounds were able to cross the blood–brain barrier (BBB) (Figure 5).

**Figure 6:**
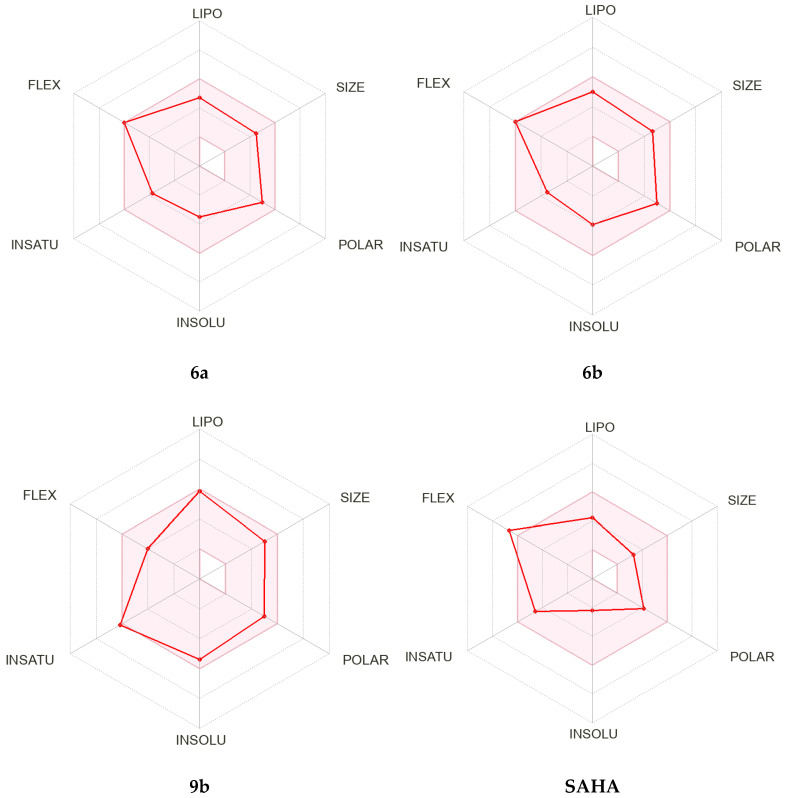
Bioavailability radar plots for compounds **6a**,**b**, **9b** and SAHA.

The bioavailability radar model considers six specific physicochemical properties for evaluation, namely lipophilicity, size, polarity, solubility, flexibility, and saturation of the final compounds (Figure 6 and Supplementary Materials Figure S3).

The output parameters are summarized in Table 4. All the final compounds showed no violation of the Lipinski rule (all have molecular weight < 500 g/mol). Furthermore, compounds **6a**,**b** and **7a**,**b** possessed higher Fsp^3^ than that of SAHA. Regarding drug resistance, it was observed that none of the compounds containing cinnamic linker **9a**–**c** exhibited any efflux ability based on glycoprotein GPG (red-colored).

## 3. Materials and Methods

### 3.1. Chemistry

All starting chemicals were purchased from Tokyo Chemical Industry (Tokyo, Japan), Kanto Chemical (Tokyo, Japan), Nacalai Tesque (Kyoto, Japan), Sigma-Aldrich (St. Louis, MO, USA), and Wako (Osaka, Japan), used without further purification. Monitoring of reaction progress was performed by thin layer chromatography (Kiesel gel 60 F254 precoated plates, Merck, St. Louis, MO, USA). The spots were detected by exposure to a UV lamp at 254 nm. Flash column chromatography was run on (40–100 mesh, Kanto Chemical, Tokyo, Japan) silica gel. NMR spectra were measured on Bruker Avance 600 (Billerica, MA, USA) (600 MHz) using tetramethylsilane as a reference. Mass spectra were determined on JEOL JMS-DX303HF (Tokyo, Japan) or BRUKER esquire3000plus-K1 (Billerica, MA, USA). Cyanoacetone and methyl (*E*)-3-aminocinnamate were prepared according to the procedure of previously reported publications [26,27] (Supplementary Materials Figures S4–S56).

Synthetic procedures of compounds **1a**–**c**

Cyclopentanone, cyclohexanone, or phenylacetaldehyde (2.08 g, 0.025 mol) was added to a mixture of freshly prepared cyanoacetone (2.08 g, 0.03 mol), elemental sulfur (0.96 g, 0.03 mol), and piperidine (2.55 g, 0.03 mol) in either methanol or ethanol (40 mL). The solution was stirred at 55–65 °C for 24 h. Then, the reaction mixture was poured into ice water. The formed precipitate was filtered off under vacuum, collected, dried, and recrystallized using the appropriate solvent [28].

**1-(2-Amino-5,6-dihydro-4*H*-cyclopenta[*b*]thiophen-3-yl)ethan-1-one (1a)**

Dark brown solid (4.2 g, 93%); MS (FAB) (m/z): 182.1 (M+H)^+^ [28].

**1-(2-Amino-4,5,6,7-tetrahydrobenzo[*b*]thiophen-3-yl)ethan-1-one (1b)**

Faint brown solid (3.75 g, 77%); ^1^H NMR (600 MHz, CDCl_3_) δ (ppm): 6.93 (s, 2H, NH_2_), 2.70–2.68 (m, 2H, CH_2_-C = C-S), 2.53–2.50 (m, 2H, CH_2_-C = C-C), 2.40 (s, 3H, -COCH_3_), 1.81–1.79 (m, 4H, -CH_2_-CH_2_- of cyclohexene). ^13^C NMR (150 MHz, CDCl_3_) δ (ppm): 194.07, 163.87, 130.65, 117.59, 115.87, 30.64, 28.06, 24.74, 23.08, 22.93. MS (FAB) (m/z):196.1 (M+H)^+^ [28].

**1-(2-Amino-5-phenylthiophen-3-yl)ethan-1-one (1c)**

Reddish brown solid (40.8 mg, 87%); ^1^H NMR (600 MHz, CDCl_3_) δ (ppm): 7.45–7.42 (m, 2H, Ar-H), 7.35–7.33 (m, 2H, Ar-H), 7.24–7.21 (m, 1H, Ar-H), 7.16 (s, 1H, Thiophene-H), 6.85 (s, 2H, NH_2_), 2.44 (s, 3H, COCH_3_). ^13^C NMR (150 MHz, CDCl_3_) δ (ppm): 193.95, 163.65, 133.91, 128.89, 126.80, 124.79, 124.33, 121.17, 116.98, 28.17. MS (FAB) (m/z): 218.2 (M+H)^+^.

Synthetic procedures of compounds **2a**–**c**

Compounds **1a**–**c** (1 g) were heated with Ac_2_O (3 mL) for 15 min. Then the reaction was quenched by heating with ethanol (5 mL). The reaction mixture was stirred with a saturated solution of NaOAc in ice until the decomposition of Ac_2_O occurred, then the resulting solid was filtered off under vacuum, washed, dried and used for the next step without further purification [18].

***N*** **-(3-Acetyl-5,6-dihydro-4*H*-cyclopenta[b]thiophen-2-yl)acetamide (2a)**

Dark green solid (1.08 g, 88%); MS (FAB) (m/z): 224 (M+H)^+^ [18].

***N*** **-(3-Acetyl-4,5,6,7-tetrahydrobenzo[b]thiophen-2-yl)acetamide (2b)**

Brown solid (1.12 g, 92%); MS (FAB) (m/z): 238.1 (M+H)^+^ [18].

***N*-(3-Acetyl-5-phenylthiophen-2-yl)acetamide (2c)**

Beige powder (1.13 g, 95%); ^1^H NMR (600 MHz, CDCl_3_) δ (ppm): 11.90 (s, 1H, NH), 7.59–7.57 (m, 2H, Ar-H), 7.39–7.34 (m, 2H, Ar-H), 7.30 (s, 1H, Thiophene-H), 7.29–7.26 (m, 1H, Ar-H), 2.55 (s, 3H, NHCOCH_3_), 2.30 (s, 3H, COCH_3_). ^13^C NMR (150 MHz, CDCl_3_) δ (ppm): 195.98, 167.79, 148.88, 133.80, 133.62, 129.03, 129.02, 127.62, 125.55, 121.69, 119.33, 28.73, 23.60. MS (FAB) (m/z): 260.2 (M+H)^+^.

Synthetic procedures of compounds **3a**–**c**

POCl_3_ (10 mL) was slowly added in a drop-wise manner to DMF (40 mL) at 0 °C over a period of 15 min. The resulting reagent was then added at once to thiophene (0.42 g, 5 mmol), and the mixture was stirred at 65 °C for 4–5 h. Upon completion of the reaction, the reaction was quenched using a mixture of ice and water and neutralized with NaOAc. If a solid was formed it was filtered off under vacuum, then dissolved in EtOAc and washed multiple times with water. The organic phase was separated, dried over anhydrous Na_2_SO_4_, and concentrated under vacuum. Subsequently, the residue was purified using column chromatography with silica gel using an EtOAc/cyclohexane mixture (1:9 ratio) as eluent. In case a solid was not obtained, the solution was directly extracted with EtOAc and purified through the same process [18,29].

**4-Chloro-6,7-dihydro-5*H*-cyclopenta[4,5]thieno[2,3-*b*]pyridine-3-carbaldehyde (3a)**

White solid (863 mg, 73%); MS (FAB) (m/z): 238 (M+H)^+^ [18].

**4-Chloro-5,6,7,8-tetrahydrobenzo[4,5]thieno[2,3-*b*]pyridine-3-carbaldehyde (3b)**

White solid (983 mg, 78%); MS (FAB) (m/z): 252 (M+H)^+^ [18].

**4-Chloro-2-phenylthieno[2,3-*b*]pyridine-5-carbaldehyde (3c)**

Faint yellow solid (1.1 g, 81%); ^1^H NMR (600 MHz, CDCl_3_) δ (ppm): 10.62–10.45 (m, 1H, CHO), 8.92 (s, 1H, Pyridine-H), 7.74–7.72 (m, 2H, Ar-H), 7.68 (s, 1H, Thiophene-H), 7.49–7.46 (m, 2H, Ar-H), 7.44–7.42 (m, 1H, Ar-H). ^13^C NMR (150 MHz, CDCl_3_) δ (ppm): 188.61, 165.73, 147.33, 146.74, 140.96, 133.08, 132.75, 129.78, 129.32, 126.73, 124.03, 114.83. MS (FAB) (m/z): 274 (M+H)^+^.

Synthetic procedures of compounds **4a**–**c** and **5a**–**c**

Compound **3a**, **3b** or **3c** (1 mmol) was dissolved in 10 mL of methanol, along with either 6-aminocaproic acid or 7-aminoheptanoic acid (1.03 mmol). The solution was heated at reflux for 3 h and monitored using TLC until the imine intermediate was fully formed. After cooling to room temperature, NaBH_4_ (60 mg, 1.6 mmol) was added gradually while stirring over 15 min. The reaction mixture was then added to an ice/water solution and acidified with glacial acetic acid. The resulting solid was filtered off under reduced pressure, collected, and dried. The resulting solid was purified using column chromatography with a mixture of MeOH and CH_2_Cl_2_ (1:9).

**6-(((4-Chloro-6,7-dihydro-5*H*-cyclopenta[4,5]thieno[2,3-*b*]pyridin-3-yl)methyl)- amino)hexanoic acid (4a)**

White solid (310 mg, 88%); mp 186–188 °C; ^1^H NMR (600 MHz, DMSO-*d*_6_) δ (ppm): 8.77 (s, 1H, Pyridine-H), 7.5 (s, 1H, NH), 4.34 (s, 2H, -C = C-CH_2_-NH), 3.20 (t, *J* = 7.4 Hz, 2H, -NH-CH_2_-CH_2_), 3.08 (t, *J* = 7.4 Hz, 2H, CH_2_-CH_2_-C = C-S), 2.94 (t, *J* = 7.4 Hz, 2H, CH_2_-CH_2_-C = C-), 2.44–2.42 (m, 2H, CH_2_-CH_2_-CH_2_-C = C-S-), 2.20 (t, *J* = 7.4 Hz, 2H, CH_2_-CH_2_-COOH), 1.73–1.71 (m, 2H, -CH_2_-CH_2_-COOH), 1.50–1.48 (m, 2H, NH-CH_2_-CH_2_-), 1.32–1.31 (m, 2H, NH-CH_2_-CH_2_-CH_2_-). ^13^C NMR (150 MHz, DMSO-*d*_6_) δ (ppm): 174.24, 166.05, 147.58, 144.89, 137.08, 136.33, 127.06, 122.35, 46.72, 44.37, 33.42, 29.75, 29.64, 26.86, 25.52, 24.96, 23.93. MS (FAB) (m/z): 353 (M+H)^+^.

**6-(((4-Chloro-5,6,7,8-tetrahydrobenzo[4,5]thieno[2,3-*b*]pyridin-3-yl)methyl)- amino)hexanoic acid (4b)**

White solid (336 mg, 92%); mp 181–183 °C; ^1^H NMR (600 MHz, MeOD) δ (ppm): 8.34 (s, 1H, Pyridine-H), 4.25 (s, 2H, -C = C-CH_2_-NH), 3.00–2.96 (m, 4H, 2CH_2_, NH-CH_2_-CH_2_ and CH_2_-CH_2_-C = C-S), 2.73 (t, *J* = 7.4 Hz, 2H, CH_2_-CH_2_-C = C-), 2.12 (t, *J* = 7.4 Hz, 2H, CH_2_-CH_2_-COOH), 1.75–1.74 (m, 4H, -CH_2_-CH_2_-C = C and –CH_2_-CH_2_-C = C-S), 1.62–1.60 (m, 2H, -CH_2_-CH_2_-COOH), 1.50–1.47 (m, 2H, NH-CH_2_-CH_2_-), 1.30–1.27 (m, 2H, NH-CH_2_-CH_2_-CH_2_-). ^13^C NMR (150 MHz, MeOD) δ (ppm): 164.22, 148.29, 142.31, 139.94, 131.81, 129.42, 123.60, 121.55, 48.60, 47.19, 35.61, 28.06, 27.20, 27.12, 26.94, 25.77, 23.56, 23.40. MS (FAB) (m/z): 367 (M+H)^+^.

**6-(((4-Chloro-2-phenylthieno[2,3-*b*]pyridin-5-yl)methyl)amino)hexanoic acid (4c)**

White solid (334 mg, 86%); mp 174–176 °C; ^1^H NMR (600 MHz, DMSO-*d*_6_) δ (ppm): 8.63 (s, 1H, Pyridine-H), 7.91–7.89 (m, 3H, Ar-H), 7.54–7.47 (m, 3H, Ar-H and NH), 4.02 (s, 2H, -C = C-CH_2_-NH), 2.62 (t, *J* = 7.2 Hz, 2H, NH-CH_2_-CH_2_), 2.20 (t, *J* = 7.4 Hz, 2H, CH_2_-CH_2_-COOH), 1.51–1.48 (m, 4H, -CH_2_-CH_2_-COOH and NH-CH_2_-CH_2_-CH_2_-), 1.34–1.32 (m, 2H, NH-CH_2_-CH_2_-CH_2_-). ^13^C NMR (150 MHz, DMSO-*d*_6_) δ (ppm): 174.46, 148.32, 144.78, 141.04, 136.63, 132.56, 132.39, 129.53, 129.36, 127.82, 126.44, 115.33, 48.27, 47.22, 33.65, 26.20, 24.36. MS (FAB) (m/z): 389 (M+H)^+^.

**7-(((4-Chloro-6,7-dihydro-5*H*-cyclopenta[4,5]thieno[2,3-b]pyridin-3-yl)methyl)- amino)heptanoic acid (5a)**

White solid (325 mg, 89%); mp 179–181 °C; ^1^H NMR (600 MHz, DMSO-*d*_6_) δ (ppm): 8.58 (s, 1H, Pyridine-H), 4.07 (s, 2H, -C = C-CH_2_-NH), 3.10 (t, *J* = 7.2 Hz, 2H, CH_2_-CH_2_-C = C-S), 3.02 (t, *J* = 7.3 Hz, 2H, CH_2_-CH_2_-C = C-), 2.68 (t, *J* = 7.4 Hz, 2H, NH-CH_2_-CH_2_), 2.43–2.42 (m, 2H, CH_2_-CH_2_-CH_2_-C = C-S-), 2.18 (t, *J* = 7.4 Hz, 2H, CH_2_-CH_2_-COOH) 1.55–1.47 (m, 4H, -CH_2_-CH_2_-COOH and NH-CH_2_-CH_2_-CH_2_-), 1.30–1.25 (m, 4H, -CH_2_-CH_2_-CH_2_-COOH and NH-CH_2_-CH_2_-CH_2_). ^13^C NMR (150 MHz, DMSO-*d*_6_) δ (ppm): 174.46, 166.24, 147.48, 145.14, 137.18, 136.35, 127.17, 122.33, 47.02, 44.61, 33.49, 29.76, 29.64, 27.95, 26.88, 25.63, 25.18, 24.18. MS (FAB) (m/z): 367 (M+H)^+^.

**7-(((4-Chloro-5,6,7,8-tetrahydrobenzo[4,5]thieno[2,3-*b*]pyridin-3-yl)methyl)-amino)heptanoic acid (5b)**

White solid (229 mg, 77%); mp 176–178 °C; ^1^H NMR (600 MHz, MeOD) δ (ppm): 8.52 (s, 1H, Pyridine-H), 4.35 (s, 2H, -C = C-CH_2_-NH), 3.19 (t, *J* = 7.2 Hz, 2H, CH_2_-CH_2_-C = C-S), 3.06 (t, *J* = 7.2 Hz, 2H, CH_2_-CH_2_-C = C-), 2.92 (t, *J* = 7.2 Hz, 2H, NH-CH_2_-CH_2_), 2.23 (t, *J* = 7.5 Hz, 2H, CH_2_-CH_2_-COOH), 1.96–1.94 (m, 4H, -CH_2_-CH_2_-C = C and –CH_2_-CH_2_-C = C-S), 1.78–1.73 (m, 2H, CH_2_-CH_2_-COOH), 1.65–1.58 (m, 2H, NH-CH_2_-CH_2_-CH_2_-), 1.48–1.40 (m, 4H, -CH_2_-CH_2_-CH_2_-COOH and NH-CH_2_-CH_2_-CH_2_). ^13^C NMR (150 MHz, MeOD) δ (ppm): 169.02, 163.55, 148.20, 141.86, 139.75, 132.03, 131.77, 129.38, 49.54, 47.59, 37.70, 30.09, 28.10, 27.91, 27.58, 27.12, 26.82, 23.59, 23.43. MS (FAB) (m/z): 381 (M+H)^+^.

**7-(((4-Chloro-2-phenylthieno[2,3-*b*]pyridin-5-yl)methyl)amino)heptanoic acid (5c)**

Yellowish white solid (330 mg, 92%); mp 173–175 °C; ^1^H NMR (600 MHz, DMSO-*d*_6_) δ (ppm): 8.66 (s, 1H, Pyridine-H), 7.90–7.87 (m, 3H, Ar-H), 7.56–7.45 (m, 3H, Ar-H), 4.05 (s, 2H, -C = C-CH_2_-NH), 2.66 (t, *J* = 7.3 Hz, 2H, NH-CH_2_-CH_2_), 2.19 (t, *J* = 7.4 Hz, 2H, CH_2_-CH_2_-COOH), 1.51–1.48 (m, 4H, CH_2_-CH_2_-COOH and NH-CH_2_-CH_2_-), 1.30–1.27 (m, 4H, -CH_2_-CH_2_-CH_2_-COOH and NH-CH_2_-CH_2_-CH_2_). ^13^C NMR (150 MHz, DMSO-*d*_6_) δ (ppm): 174.51, 159.44, 148.40, 144.83, 137.22, 136.75, 132.53, 132.38, 129.52, 129.34, 126.42, 115.32, 48.20, 46.91, 33.67, 28.38, 28.19, 26.28, 24.44. MS (FAB) (m/z): 403 (M+H)^+^.

Synthetic Procedures of compounds **6a**–**c** and **7a**–**c**

The appropriate acid derivative (0.25 mmol) was dissolved in anhydrous DMF (3 mL), then *N,N′*- carbonyldiimidazole (48.3 mg, 0.3 mmol) was added in a portion-wise manner, and the mixture was allowed to stir at ambient temperature for 4 h; then, solid hydroxylamine hydrochloride (0.075 g, 10 mmol) was added, and the mixture was stirred overnight. The reaction was quenched by addition of cold water and acidified with glacial acetic acid. The final products were obtained by filtration under reduced pressure and washing with water and recrystallization with an appropriate solvent.

**6-(((4-Chloro-6,7-dihydro-5H-cyclopenta[4,5]thieno[2,3-*b*]pyridin-3-yl)methyl)- amino)-*N*-hydroxyhexanamide (6a)**

White solid (55 mg, 61%) recrystallized from ethanol; m.p: 170–172 °C; ^1^H NMR (600 MHz, MeOD) δ (ppm): 8.30 (s, 1H, Pyridine-H), 4.84 (s, 2H, -C = C-CH_2_-NH), 3.52 (m, 2H, CH_2_-CH_2_-C = C-S), 3.35 (m, 2H, CH_2_-CH_2_-C = C-), 3.20–3.19 (m, 2H, NH-CH_2_-CH_2_), 3.07–3.06 (m, 2H, CH_2_-CH_2_-C = C-S-), 2.66 (t, *J* = 7.4 Hz, 2H, CH_2_-CH_2_-CONHOH), 2.55–2.52 (m, 2H, CH_2_-CH_2_-CONHOH), 1.78–1.71 (m, 2H, NH-CH_2_-CH_2_), 1.57–1.54 (m, 2H, NH-CH_2_-CH_2_-CH_2_). ^13^C NMR (150 MHz, MeOD) δ (ppm): 178.87, 166.91, 146.98, 146.55, 138.09, 137.96, 129.50, 128.47, 50.57, 47.55, 37.67, 31.14, 30.88, 30.72, 29.11, 28.43, 24.38. HRMS (ESI) m/z [M+H]^+^: Calcd. for C_17_H_23_ClN_3_O_2_S: 368.1200, Found, 368.1271.

**6-(((4-Chloro-5,6,7,8-tetrahydrobenzo[4,5]thieno[2,3-*b*]pyridin-3-yl)methyl)- amino)-*N*-hydroxyhexanamide (6b)**

Yellowish white solid (63 mg, 66%) recrystallized from ethanol; m.p: 167–169 °C; ^1^H NMR (600 MHz, MeOD) δ (ppm): 8.16 (s, 1H, Pyridine-H), 4.70 (s, 2H, -C = C-CH_2_-NH), 3.38 (t, *J* = 7.3 Hz, 2H, CH_2_-CH_2_-C = C-S), 3.04 (t, *J* = 7.3 Hz, 2H, CH_2_-CH_2_-C = C-), 2.77 (t, *J* = 7.3 Hz, 2H, NH-CH_2_-CH_2_), 2.54 (t, *J* = 7.4 Hz, 2H, CH_2_-CH_2_-CONHOH), 1.81–1.79 (m, 4H, -CH_2_-CH_2_-CH_2_-C = C-S-), 1.65–1.60 (m, 4H, -CH_2_-CH_2_-CONHOH and NH-CH_2_-CH_2_), 1.43 (t, *J* = 7.4 Hz, 2H, NH-CH_2_-CH_2_-CH_2_). ^13^C NMR (150 MHz, MeOD) δ (ppm): 178.88, 162.07, 147.16, 141.21, 138.73, 131.73, 129.22, 128.61, 50.53, 47.80, 37.68, 30.73, 29.09, 28.18, 27.11, 24.39, 23.64, 23.46. HRMS (ESI) m/z [M+H]^+^: Calcd. for C_18_H_25_ClN_3_O_2_S: 382.1356, Found, 382.1462.

**6-(((4-Chloro-2-phenylthieno[2,3-*b*]pyridin-5-yl)methyl)amino)-*N*-hydroxy-hexanamide (6c)**

Yellowish white (68.5 mg, 68%) recrystallized from aqueous ethanol; m.p: 162–164 °C; ^1^H NMR (600 MHz, DMSO-*d*_6_) δ (ppm): 8.44 (s, 1H, Pyridine-H), 7.92–7.89 (m, 3H, Ar-H), 7.54–7.47 (m, 3H, Ar-H), 4.75 (s, 2H, -C = C-CH_2_-NH), 3.46 (t, *J* = 7.3 Hz, 2H, NH-CH_2_-CH_2_), 2.56 (t, *J* = 7.4 Hz, 2H, CH_2_-CH_2_-CONHOH), 1.70–1.56 (m, 4H, -CH_2_-CH_2_-CONHOH and NH-CH_2_-CH_2_), 1.50–1.41 (m, 2H, NH-CH_2_-CH_2_-CH_2_). ^13^C NMR (150 MHz, DMSO-*d*_6_) δ (ppm): 175.09, 159.42, 147.54, 145.03, 136.16, 132.52, 132.50, 129.56, 129.35, 127.92, 126.45, 115.23, 48.59, 46.06, 36.32, 29.08, 27.89, 22.91. HRMS (ESI) m/z [M+H]^+^: Calcd. for C_20_H_23_ClN_3_O_2_S: 404.1200, Found, 404.1296.

**7-(((4-Chloro-6,7-dihydro-5*H*-cyclopenta[4,5]thieno[2,3-*b*]pyridin-3-yl)methyl)- amino)-*N*-hydroxyheptanamide (7a)**

Yellowish white (67.8 mg, 71%) recrystallized from ethanol; mp 166–168 °C; ^1^H NMR (600 MHz, MeOD) δ (ppm) 8.40 (s, 1H, Pyridine-H), 4.02 (s, 2H, -C = C-CH_2_-NH), 3.21–3.19 (m, 2H, CH_2_-CH_2_-C = C-S), 3.07–3.06 (m, 2H, CH_2_-CH_2_-C = C-), 2.67 (t, *J* = 7.4 Hz, 2H, NH-CH_2_-CH_2_), 2.54–2.52 (m, 2H, -CH_2_-CH_2_-C = C-S-), 2.10 (t, *J* = 7.4 Hz, 2H, CH_2_-CH_2_-CONHOH), 1.64–1.59 (m, 4H, -CH_2_-CH_2_-CONHOH and NH-CH_2_-CH_2_), 1.38–1.37 (m, 4H, -CH_2_-CH_2_-CH_2_-CONHOH and NH-CH_2_-CH_2_-CH_2_). ^13^C NMR (150 MHz, MeOD) δ (ppm): 171.53, 165.31, 145.93, 144.86, 137.10, 136.59, 128.75, 128.12, 48.59, 32.29, 29.74, 29.46, 29.27, 28.80, 28.56, 27.01, 26.56, 25.23. HRMS (ESI) m/z [M+H]^+^: Calcd. for C_18_H_25_ClN_3_O_2_S: 382.1356, Found, 382.1457.

**7-(((4-Chloro-5,6,7,8-tetrahydrobenzo[4,5]thieno[2,3-*b*]pyridin-3-yl)methyl)- amino)-*N*-hydroxyheptanamide (7b)**

Yellowish white solid (4.3 mg, 65%) recrystallized from ethanol; mp 163–165 °C; ^1^H NMR (600 MHz, DMSO-*d*_6_) δ (ppm): 10.39 (s, 1H, CONHOH), 8.43 (s, 1H, Pyridine-H), 3.87 (s, 2H, -C = C-CH_2_-NH), 3.06 (t, *J* = 7.4 Hz, 2H, CH_2_-CH_2_-C = C-S), 2.85 (t, *J* = 7.4 Hz, 2H, CH_2_-CH_2_-C = C-), 2.52 (t, *J* = 7.4 Hz, 2H, NH-CH_2_-CH_2_), 1.92 (t, *J* = 7.4 Hz, 2H, CH_2_-CH_2_-CONHOH), 1.82–1.80 (m, 4H, CH_2_-CH_2_-CH_2_-C = C-S-), 1.45–1.40 (m, 4H, CH_2_-CH_2_-CONHOH and NH-CH_2_- CH_2_), 1.26–1.24 (m, 4H, -CH_2_-CH_2_-CH_2_-CONHOH and NH-CH_2_-CH_2_- CH_2_). ^13^C NMR (150 MHz, DMSO-*d*_6_) δ (ppm): 169.43, 159.43, 150.53, 146.66, 138.76, 136.52, 129.29, 127.45, 48.50, 47.68, 32.16, 29.08, 28.42, 26.57, 26.40, 25.61, 25.03, 21.98, 21.81. HRMS (ESI) m/z [M+H]^+^: Calcd. for C_19_H_27_ClN_3_O_2_S: 396.1513, Found, 396.1603.

**7-(((4-Chloro-2-phenylthieno[2,3-*b*]pyridin-5-yl)methyl)amino)-*N*-hydroxy- heptanamide (7c)**

Yellowish white solid (70 mg, 67%) recrystallized from aqueous ethanol; mp 159–162 °C; ^1^H NMR (600 MHz, MeOD) δ (ppm): 8.55 (s, 1H, Pyridine-H), 7.85–7.77 (m, 3H, Ar-H), 7.53–7.45 (m, 3H, Ar-H), 4.09 (s, 2H, C = C-CH_2_-NH), 2.72 (t, *J* = 7.3 Hz, 2H, NH-CH_2_-CH_2_), 2.11 (t, *J* = 7.4 Hz, 2H, CH_2_-CH_2_-CONHOH), 1.66–1.61 (m, 4H, CH_2_-CH_2_-CONHOH and NH-CH_2_- CH_2_), 1.41–1.38 (m, 4H, CH_2_-CH_2_-CH_2_-CONHOH and NH-CH_2_-CH_2_- CH_2_). ^13^C NMR (150 MHz, MeOD) δ (ppm): 172.99, 161.47, 149.00, 147.57, 139.09, 134.69, 134.44, 130.65, 130.42, 130.41, 127.66, 116.07, 50.01, 49.46, 33.70, 30.01, 29.93, 27.93, 26.62. HRMS (ESI) m/z [M+H]^+^: Calcd. for C_21_H_25_ClN_3_O_2_S: 418.1356, Found, 418.1450.

Synthetic procedures of compounds **8a**–**c**

Methyl (*E*)-3-aminocinnamate (0.5 mmol) was added to a solution of an appropriate aldehyde, **3a**–**c** (0.55 mmol), in 1,2 DCE for 30 min. Next, sodium triacetoxyborohydride (148.4 mg, 0.7 mmol) was added, and the mixture was continuously stirred for 3 h. After completion of the initial reaction, the saturated solution of sodium bicarbonate was used to quench the reaction. Subsequently, the mixture was extracted three times with DCM (10 mL each time). The combined organic layer was then dried using anhydrous Na_2_SO_4_, and the solvent was removed under reduced pressure. The resulting precipitate was further purified through column chromatography, utilizing a DCM and MeOH mixture as the elution system [20,30].

**Methyl (*E*)-3-(3-(((4-chloro-6,7-dihydro-5*H*-cyclopenta[4,5]thieno[2,3-*b*]pyridin-3-yl)methyl)amino)phenyl)acrylate (8a)**

White solid (183 mg, 92%); mp 142–144 °C; ^1^H NMR (600 MHz, CDCl_3_) δ (ppm): 8.38 (s, 1H, Pyridine-H), 7.59 (d, *J* = 16.0 Hz, 1H, -CH = CH-), 7.19–7.17 (m, 1H, Ar-H), 6.91–6.90 (m, 1H, Ar-H), 6.77 (s, 1H, Ar-H), 6.69–6.67 (m, 1H, Ar-H), 6.36 (d, *J* = 16.0 Hz, 1H, -CH = CH-), 4.53 (s, 2H, C = C-CH_2_-NH), 3.78 (s, 3H, OCH_3_), 3.24 (t, *J* = 7.4 Hz, 2H, CH_2_-CH_2_-C = C-S), 3.05 (t, *J* = 7.4 Hz, 2H, CH_2_-CH_2_-C = C-) 2.50–2.47 (m, 2H, -CH_2_-CH_2_-C = C-S-). ^13^C NMR (150 MHz, CDCl_3_) δ (ppm): 167.49, 166.31, 147.75, 145.39, 145.31, 144.89, 136.61, 136.50, 135.49, 129.83, 128.30, 127.61, 118.13, 117.67, 115.20, 112.19, 51.63, 43.72, 30.23, 30.15, 27.44. MS (FAB) (m/z): 399 (M+H)^+^.

**Methyl (*E*)-3-(3-(((4-chloro-5,6,7,8-tetrahydrobenzo[4,5]thieno[2,3-*b*]pyridin-3-yl)methyl)amino)phenyl)acrylate (8b)**

White solid (185 mg, 90%); mp 139–142 °C; ^1^H NMR (600 MHz, CDCl_3_) δ (ppm): 8.36 (s, 1H, Pyridine-H), 7.58 (d, *J* = 16.0 Hz, 1H, -CH = CH-), 7.18–7.16 (m, 1H, Ar-H), 6.90 (d, *J* = 7.00 Hz, 1H, Ar-H), 6.75 (s, 1H, Ar-H), 6.68–6.66 (m, 1H, Ar-H), 6.35 (d, *J* = 16.0 Hz, 1H, -CH = CH-), 4.52 (s, 2H, C = C-CH_2_-NH), 3.78 (s, 3H, OCH_3_), 3.14 (t, *J* = 7.4 Hz, 2H, CH_2_-CH_2_-C = C-S), 2.85 (t, *J* = 7.4 Hz, 2H, CH_2_-CH_2_-C = C-), 1.90–1.87 (m, 4H, CH_2_-CH_2_-CH_2_-C = C-S-). ^13^C NMR (150 MHz, CDCl_3_) δ (ppm): 167.50, 161.42, 147.82, 145.69, 145.35, 139.57, 137.11, 135.46, 130.42, 129.81, 127.86, 127.78, 118.04, 117.61, 115.17, 112.15, 51.63, 43.93, 27.01, 26.32, 22.61, 22.43. MS (FAB) (m/z): 413 (M+H)^+^.

**Methyl (*E*)-3-(3-(((4-chloro-2-phenylthieno[2,3-*b*]pyridin-5-yl)methyl)amino)- phenyl)acrylate (8c)**

Yellowish white solid (193 mg, 89%); mp 128–130 °C; ^1^H NMR (600 MHz, DMSO-*d*_6_) δ (ppm): 8.56 (s, 1H, Pyridine-H), 7.94–7.89 (m, 3H, Ar-H), 7.55–7.46 (m, 4H, Ar-H, NH and CH = CH-), 7.16–7.14 (m, 1H, Ar-H), 6.96–6.92 (m, 2H, Ar-H), 6.73 (d, *J* = 7.0 Hz, 1H, Ar-H), 6.52–6.47 (m, 2H, Ar-H and CH = CH-), 4.56 (s, 2H, C = C-CH_2_-NH), 3.71 (s, 3H, OCH_3_). ^13^C NMR (150 MHz, DMSO-*d*_6_) δ (ppm): 166.70, 159.33, 158.60, 148.48, 147.42, 145.38, 144.90, 144.46, 136.44, 134.65, 132.55, 129.54, 129.35, 129.11, 126.44, 117.06, 116.81, 115.17, 115.09, 111.15, 51.37, 42.14. MS (FAB) (m/z): 435 (M+H)^+^.

**Synthetic Procedures of compounds 9a–c**

A suitable ester intermediate, **8a**–**c** (0.25 mmol), was dissolved in MeOH (5 mL). Following that, a solution containing 50 wt. % NH_2_OH in H_2_O (0.3 mL) was added along with a solution of 4 M KOH (1 mL, 10 equivalents). The resulting mixture was stirred for 4 h. Afterward, the solution was diluted with water and made acidic by adding dilute HCl. The ensuing precipitate was filtered off, collected, dried, and subsequently recrystallized using a mixture of MeOH and H_2_O.

**(*E*)-3-(3-(((4-chloro-6,7-dihydro-5*H*-cyclopenta[4,5]thieno[2,3-*b*]pyridin-3-yl)- methyl)amino)phenyl)-*N*-hydroxyacrylamide (9a)**

Yellowish white solid (62.8 mg, 63%); mp 153–155 °C; ^1^H NMR (600 MHz, DMSO-*d*_6_) δ (ppm): 10.73 (s, 1H, NHOH), 9.00 (s, 1H, NHOH), 8.42 (s, 1H, Pyridine-H), 7.32 (d, *J* = 15.8 Hz, 1H, CH = CH-), 7.12–7.10 (m, 1H, Ar-H), 6.80 (s, 1H, Ar-H), 6.77 (d, *J* = 7.4 Hz, 1H, Ar-H), 6.63 (d, *J* = 7.4 Hz, 1H, Ar-H), 6.43 (bs, 1H, NH), 6.35 (d, *J* = 15.8 Hz, 1H, CH = CH-), 4.48 (s, 2H, C = C-CH_2_-NH), 3.13 (t, *J* = 7.4 Hz, 2H, CH_2_-CH_2_-C = C-S), 3.02 (t, *J* = 7.4 Hz, 2H, CH_2_-CH_2_-C = C-), 2.46–2.41 (m, 2H, CH_2_-CH_2_-C = C-S). ^13^C NMR (150 MHz, DMSO-*d*_6_) δ (ppm): 164.71, 162.89, 148.45, 145.69, 144.24, 139.11, 136.08, 135.72, 135.46, 129.50, 128.70, 127.25, 118.37, 115.81, 113.67, 110.93, 42.05, 29.69, 26.88. HRMS (ESI) m/z [M+H]^+^: Calcd. for C_20_H_19_ClN_3_O_2_S: 400.0887, Found, 400.0976

**(*E*)-3-(3-(((4-Chloro-5,6,7,8-tetrahydrobenzo[4,5]thieno[2,3-*b*]pyridin-3-yl)methyl)- amino)phenyl)-*N*-hydroxyacrylamide (9b)**

Yellowish white solid (69.2 mg, 67%); mp 150–152 °C; ^1^H NMR (600 MHz, DMSO-*d*_6_) δ (ppm): 10.71 (s, 1H, NHOH), 8.98 (s, 1H, NHOH), 8.41 (s, 1H, Pyridine-H), 7.31 (d, *J* = 15.8 Hz, 1H, CH = CH-), 7.11 (t, *J* = 7.8 Hz, 1H, Ar-H), 6.79 (s, 1H, Ar-H), 6.77 (d, *J* = 7.6 Hz, 1H, Ar-H), 6.62 (d, *J* = 7.6 Hz, 1H, Ar-H), 6.40 (bs, 1H, NH), 6.33 (d, *J* = 15.8 Hz, 1H, CH = CH-), 4.47 (s, 2H, C = C-CH_2_-NH), 3.11 (t, *J* = 7.4 Hz, 2H, CH_2_-CH_2_-C = C-S), 2.86 (t, *J* = 7.4 Hz, 2H, CH_2_-CH_2_-C = C-), 1.89–1.80 (m, 4H, CH_2_-CH_2_-CH_2_-C = C-S-). ^13^C NMR (150 MHz, DMSO-*d*_6_) δ (ppm): 162.78, 159.79, 148.47, 145.98, 138.97, 136.43, 136.42, 135.46, 129.51, 129.38, 128.83, 127.43, 118.35, 115.79, 113.67, 110.89, 48.58, 26.58, 25.66, 22.03, 21.86. HRMS (ESI) m/z [M+H]^+^: Calcd. for C_21_H_21_ClN_3_O_2_S: 414.1043, Found, 414.1140.

**(*E*)-3-(3-(((4-Chloro-2-phenylthieno[2,3-*b*]pyridin-5-yl)methyl)amino)phenyl)-*N*-hydroxyacrylamide (9c)**

Yellowish white solid (75 mg, 69%); mp 185–187 °C; ^1^H NMR (600 MHz, DMSO-*d*_6_) δ (ppm): 10.78 (s, 1H, NHOH), 10.28 (s, 1H, NHOH), 8.55 (s, 1H, Pyridine-H), 7.93–7.89 (m, 3H, Ar-H and -CH = CH-), 7.54–7.46 (m, 4H, Ar-H), 7.30 (s, 1H, Ar-H), 6.83–6.56 (m, 3H, Ar-H), 6.38 (d, *J* = 15.8 Hz, 1H, -CH = CH-), 4.53 (s, 2H, C = C-CH_2_-NH). ^13^C NMR (150 MHz, DMSO-*d*_6_) δ (ppm): 159.32, 148.43, 147.41, 144.92, 139.20, 136.40, 135.50, 132.56, 132.53, 129.54, 129.35, 129.26, 129.12, 126.44, 125.64, 118.44, 115.88, 115.18, 113.72, 110.98, 42.16. HRMS (ESI) m/z [M+H]^+^: Calcd. for C_23_H_19_ClN_3_O_2_S: 436.0887, Found, 436.0982.

### 3.2. NCI-60 Screening of Anticancer Activity

#### 3.2.1. One-Dose Assay

The NCI procedures for screening anticancer compounds are shown on the website https://dtp.cancer.gov/ (accessed on 2 March 2026). These entail testing potential anticancer candidates against a set of sixty cell lines derived from nine distinct human tumors. The NCI-60 testing adheres to a specific protocol established by the Drug Evaluation Branch of the National Cancer Institute in Bethesda, USA. The screening is executed at a consistent concentration of 10^−5^ M, or 15 μg/mL. Endpoint determinations were made using a protein binding dye, SRB. Absorbance was assessed spectrophotometrically, with results for each tested compound presented as the percentage of growth inhibition compared to untreated cells.

#### 3.2.2. Five-Dose Assay

Compounds showing significant cell growth inhibition during the initial screening were subjected to additional testing on a range of 58–60 cells at different concentration levels. The drugs were first dissolved in dimethyl sulfoxide, introduced to the cells, and left to incubate at 37 °C, with 5% CO_2_, 95% air, and 100% relative humidity, for a period of 48 h. Following incubation, the cells were stained with SRB and the absorbance levels were determined using an automated plate reader. The growth percentage was then calculated for various concentrations and timeframe intervals.

### 3.3. Antiproliferative Assay Against Normal PBMCs

PBMCs procured from Precision for Medicine in Bethesda, MD, USA, were cultured in RPMI-1640 medium with 10% FBS and plated onto a 96-well plate at a concentration of 5 × 10^5^ cells per 300 μL per well. Subsequently, compound **7a**, dissolved in 3 μL of dimethyl sulfoxide (DMSO), was introduced, and the cells were allowed to incubate for 2 days. Following this incubation period, the MTT assay was conducted in accordance with the previously described procedures [31].

### 3.4. Evaluation of Apoptotic Gene Caspase-3 Expression

The RPMI-8226 cell line was cultured in RPMI-1640 supplemented in 10% FBS. Immunoblot analysis was performed using cells lysed in PBS/Laemmli sample buffer (1:1) as previously described [32]. As antibodies, caspase-3 (D3R6Y) Rabbit mAb (Cell Signalling, Danvers, MA, USA, 1:1000) and mouse monoclonal anti-β-Actin antibody (AC-15) (Sigma-Aldrich, Burlington, MA, USA, 1:1000) were used. Immunoreactivity was detected by chemiluminescence using ImmunoStar LD (Wako).

### 3.5. HDAC Inhibitory Activity Assay

A fluorogenic assay was used to evaluate the enzymatic activities of HDACs, including HDAC1, HDAC4, and HDAC6, as described previously [33,34]. HDAC proteins purified from 293 T or HeLa cells were incubated at 37 °C for 30 min with a fluorescent peptide substrate (2 mM Ac-KGLGK(Ac)-MCA) in 20 μL of HDAC assay buffer (20 mM Tris–HCl (pH 8.0), 150 mM NaCl, and 10% glycerol). The reaction was then terminated by the addition of 20 μL of trypsin (20 mg/mL), followed by incubation at 37 °C for 15 min. The released aminomethylcoumarin (AMC) was measured using a fluorescence plate reader (Molecular Devices, San Jose, CA, USA).

### 3.6. Docking Study

The molecular operating environment software version MOE 2019 was utilized for conducting the molecular docking studies. Initially, the co-crystalized HDLP protein with **SAHA** (**PDB: 1ZZ1**) was downloaded from https://www.rcsb.org/. A library of target compounds, **Panobinostat** and **SAHA**, was drawn from, and their energy was minimized using the Hamiltonian-force field-MMFF94x method, followed by the calculation of force field partial charges for each molecule [35]. Subsequently, 3D protonation and correction were performed, and any water molecules were removed. The docking process employed the triangle matcher placement method, and the rescoring function utilized was London DG [36]. To validate the docking methodology, redocking of **SAHA** into the active site of the enzyme was carried out, and the results demonstrated an identical alignment to the original one, along with identical interactions.

### 3.7. In Silico Physicochemical and Pharmacokinetic Prediction

The Swiss Institute of Bioinformatics provides several free services, including SwissADME, which was utilized to analyze the physicochemical properties and pharmacokinetics of compounds **6a**–**c**, **7a**–**c**, and **9a**–**c**. BOILED-Egg is a plot of TPSA versus WLOGP, with the white region representing the highest probability of gastrointestinal absorption, and the yolk region representing the highest probability of BBB permeability. Lipophilicity is expressed in consensus log Po/w, which is calculated by SwissADME, and it equals the arithmetic mean of the five different log *p* values predicted by different freely available models, namely XLOGP3, MLOGP, SILICOS-IT, and iLOGP, in addition to their own model WLOGP, which is also involved in the BOILED-Egg plot. The bioavailability radar represents six physicochemical properties:

Lipophilicity (−0.7 < XLOGP3 > +5.0), size (150 g/mol < MV > 500 g/mol), polarity (20 Å^2^ < TPSA > 130 Å^2^), insolubility (0 < Log S (ESOL) > 6), instauration (0.25 < Fraction Csp3 > 1.0), and flexibility (0 < no. of rotatable bonds > 9). The central pink hexagon represents the optimum range for all six parameters. The Lipinski filter is used to assess the drug-likeness of synthesized compounds (MW ≤ 500, MLOGP ≤ 4.15, N or O ≤ 10, NH or OH ≤ 5) [25].

## 4. Conclusions

Harnessing substituted 4-chlorothieno[2,3-*b*]pyridine scaffold as a new cap for HDACs inhibitors, alongside using variable linkers with variable length and flexibility, led to the identification of hitherto unreported strong HDAC inhibitors with prominent antiproliferative activity as confirmed by NCI-60 screening. Of interest, compound **7a** stands out as a sub micromolar inhibitor of HDACs 1, 4, and 6. Its low cytotoxicity on PBMCs makes it a promising lead compound with a favorable safety rating. The cytotoxicity of **7a** and **9a** is attributed to the induction of apoptosis, as shown by their effect on caspase-3 in RPMI-8226 cells, placing them in the forefront of our series. In sum, our study highlights the importance of utilizing 4-chlorothieno[2,3-*b*]pyridine scaffolds for developing new HDAC inhibitors. Continued exploration and discovery of derivatives based on this privileged structure may lead to further tangible advancements in anticancer drug discovery.

## Figures and Tables

**Table 1:** NCI one-dose screening results for final compounds **6a**–**c**, **7a**–**c,** and **9a**–**c** (GI%) *.

	6a	6b	6c	7a	7b	7c	9a	9b	9c
CCRF-CEM	24.01	9.16	106.46	82.60	99.01	97.30	86.62	118.37	104.52
HL-60(TB)	22.27	6.96	120.35	93.82	120.74	111.86	98.97	108.14	44.44
K-562	28.28	20.52	86.02	85.52	85.42	66.67	92.99	92.63	61.18
MOLT-4	30.00	23.12	78.25	75.61	89.67	80.17	90.29	96.08	80.27
RPMI-8226	29.36	23.22	100.93	97.53	96.64	92.54	93.51	110.11	86.54
SR	30.13	24.68	58.85	72.04	ND	58.27	87.98	86.25	76.15
A549/ATCC	5.00	8.50	64.06	89.48	70.37	53.40	121.20	99.49	65.40
EKVX	13.38	20.08	62.57	81.87	49.83	59.73	120.14	113.99	67.93
HOP-62	7.49	7.23	65.08	98.58	63.07	53.12	155.19	145.74	57.77
HOP-92	−7.40	8.68	103.61	102.36	90.34	84.37	121.99	119.74	27.40
NCI-H226	16.86	15.11	21.76	42.55	16.77	21.80	122.62	87.67	16.47
NCI-H23	2.04	5.85	53.32	96.03	62.95	48.42	170.69	160.15	46.98
NCI-H322M	14.10	15.67	60.67	98.23	58.80	56.41	106.15	96.97	57.95
NCI-H460	12.29	11.70	72.83	84.66	69.53	65.73	128.32	133.74	83.76
NCI-H522	29.98	25.76	86.81	101.96	74.90	75.53	166.02	150.73	71.40
COLO 205	−6.34	−4.85	74.95	169.23	129.98	68.05	165.93	161.95	18.55
HCC-2998	1.20	−1.21	47.36	110.66	65.37	49.54	167.51	141.57	26.86
HCT-116	10.91	15.92	87.25	95.39	93.28	86.76	123.11	96.93	77.26
HCT-15	6.77	12.97	61.49	90.05	48.96	52.78	125.70	92.25	84.53
HT29	7.71	10.93	82.88	95.59	85.53	74.60	95.58	87.19	69.44
KM12	2.06	3.76	49.87	88.50	63.57	37.86	124.99	101.17	56.84
SW-620	1.12	11.11	118.38	98.61	99.20	106.03	100.31	112.33	64.26
SF-268	0.64	−1.55	74.53	95.81	63.42	49.36	134.59	122.90	36.44
SF-295	9.74	16.14	74.50	145.22	81.74	64.91	175.66	159.35	19.52
SF-539	15.25	11.25	65.67	87.65	60.65	63.42	170.44	158.87	44.36
SNB-19	27.61	25.04	49.53	77.99	52.48	43.68	154.42	131.87	31.18
SNB-75	36.40	26.85	89.71	93.58	48.49	85.02	115.33	104.24	59.18
U251	36.71	20.82	70.69	95.77	70.87	56.13	176.04	149.19	65.24
LOX IMVI	−0.06	−2.02	105.44	163.31	95.25	97.97	189.06	191.11	86.92
MALME-3M	25.39	20.21	96.16	107.93	97.98	95.96	122.46	114.15	38.64
M14	−4.00	−1.26	53.68	88.35	69.89	50.09	156.81	133.37	28.86
MDA-MB-435	8.71	5.88	89.11	154.83	92.43	87.09	177.97	137.57	54.88
SK-MEL-2	−5.74	11.61	97.31	124.41	104.11	78.06	125.61	57.04	14.60
SK-MEL-28	0.10	−0.75	74.54	83.82	67.32	71.40	166.77	95.16	25.10
SK-MEL-5	5.22	6.65	64.25	144.11	113.38	63.98	189.02	154.34	14.00
UACC-257	5.42	4.50	57.89	80.68	82.31	56.46	97.47	79.18	11.55
UACC-62	22.09	21.35	76.55	118.57	88.45	74.98	186.57	177.33	58.37
IGROV1	5.57	6.13	67.04	133.14	57.69	59.38	180.08	166.05	49.58
OVCAR-3	6.60	5.43	80.12	98.09	70.43	63.63	129.21	123.45	86.68
OVCAR-4	16.05	19.60	41.03	59.61	41.13	35.99	101.49	98.77	65.01
OVCAR-5	1.51	6.42	74.56	95.99	82.81	72.49	127.59	100.63	35.26
OVCAR-8	21.95	16.70	71.56	88.37	88.99	68.61	147.20	141.04	52.49
NCI/ADR-RES	18.72	19.03	98.52	106.90	59.38	93.09	149.76	148.94	61.10
SK-OV-3	3.83	2.88	98.20	140.01	101.51	66.06	167.15	166.34	26.97
786-0	9.09	14.58	42.08	76.61	49.71	25.70	147.54	105.90	11.83
A498	16.22	14.24	93.50	111.66	73.98	69.98	72.46	45.74	21.94
ACHN	−3.34	−2.82	80.00	128.83	70.15	74.81	168.73	161.28	89.57
CAKI-1	25.82	15.38	80.69	118.67	81.95	73.78	156.02	144.51	93.16
RXF 393	5.33	2.68	68.28	118.76	70.41	77.78	140.07	114.75	35.69
SN12C	11.49	10.32	55.30	101.65	41.55	48.18	150.87	126.56	53.23
TK-10	1.97	14.73	71.06	98.39	64.60	58.09	94.93	82.40	53.92
UO-31	30.29	22.18	77.08	154.50	62.82	67.99	187.83	166.75	67.23
PC-3	18.08	9.83	74.73	75.37	63.23	65.31	86.45	95.75	71.33
DU-145	10.23	13.25	78.26	90.05	78.75	64.04	128.17	115.92	69.00
MCF7	22.09	17.95	73.51	99.24	75.32	69.76	116.60	113.88	77.84
MDA-MB-231/ATCC	12.23	7.92	85.42	108.08	83.58	80.32	128.27	104.93	53.97
HS 578T	17.49	16.67	78.50	81.46	76.12	70.58	79.14	85.65	14.22
BT-549	0.93	−2.98	36.76	53.08	38.68	31.13	113.87	87.09	29.45
T-47D	ND	16.42	77.63	99.34	101.67	69.43	101.72	105.39	69.10
MDA-MB-468	40.42	ND	ND	ND	103.09	ND	ND	ND	ND
Mean	12.84	11.63	74.7	100.86	75.6	66.87	133.55	120.01	52.94

* 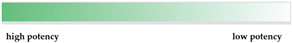

**Table 2:** NCI five-dose results for compounds **6c**, **7a**,**b**, **9a**,**b** and **SAHA** (GI_50_, µM). ND (not determined); red-colored (more potent than **SAHA**).

Cell Line Name/Cpd	6c	7a	7b	9a	9b	SAHA
**Leukemia**						
CCRF-CEM	1.53	0.98	2.32	0.61	0.78	0.37
HL-60(TB)	1.38	2.19	2.58	1.99	1.92	0.47
K-562	2.04	1.49	2.10	2.73	2.92	ND
MOLT-4	1.75	1.34	2.01	1.47	1.70	0.32
RPMI-8226	2.03	1.32	1.78	1.38	1.71	1.48
SR	1.37	0.56	ND	0.67	0.82	0.37
**Non-Small Cell Lung**						
A549/ATCC	3.20	2.05	3.45	1.76	1.72	1.73
EKVX	4.58	3.28	3.97	3.01	2.75	1.86
HOP-62	2.73	2.77	2.98	1.81	1.88	1.68
HOP-92	1.59	2.07	2.57	2.05	2.14	0.25
NCI-H226	8.97	9.45	12.20	1.87	1.85	2.76
NCI-H23	5.26	3.24	3.99	1.77	1.93	1.78
NCI-H322M	3.57	2.08	4.74	1.88	1.96	2.02
NCI-H460	2.90	1.96	2.79	1.57	1.71	0.88
NCI-H522	2.17	2.20	2.05	1.49	1.54	0.50
**Colon**						
COLO 205	2.20	1.50	2.10	1.75	1.85	1.14
HCC-2998	4.17	2.64	2.30	1.67	1.99	1.24
HCT-116	2.50	1.29	2.44	1.84	1.70	0.38
HCT-15	3.72	2.82	7.26	0.55	0.69	3.06
HT29	2.16	1.73	2.65	1.89	2.36	0.63
KM12	3.59	1.95	2.34	2.07	2.03	1.90
SW-620	1.59	1.29	2.10	1.83	1.88	0.49
**Central Nervous System**						
SF-268	3.19	2.05	3.68	1.72	1.73	1.81
SF-295	4.60	2.03	4.16	1.84	1.87	1.05
SF-539	3.03	2.25	5.97	1.72	1.68	1.86
SNB-19	5.48	3.00	5.92	1.76	1.89	1.95
SNB-75	2.25	1.94	2.20	1.76	2.62	0.54
U251	2.49	1.89	3.49	1.51	1.51	0.95
**Melanoma**						
LOX IMVI	2.24	2.27	1.71	1.56	1.69	1.17
MALME-3M	1.31	0.61	4.59	1.71	1.70	0.35
M14	3.48	2.40	4.46	1.48	1.67	0.94
MDA-MB-435	2.43	1.27	2.33	1.82	2.01	0.55
SK-MEL-2	2.48	2.40	3.47	1.98	1.92	1.27
SK-MEL-28	2.77	2.83	6.79	2.89	3.00	0.82
SK-MEL-5	3.39	1.83	1.97	1.83	1.84	0.47
UACC-257	2.14	0.86	1.65	2.07	2.15	0.63
UACC-62	2.45	1.48	ND	1.70	1.76	0.35
**Ovarian**						
IGROV1	3.88	2.22	5.03	1.65	1.89	2.15
OVCAR-3	2.77	1.41	3.70	1.39	1.72	1.52
OVCAR-4	3.66	2.56	6.25	1.84	2.30	2.70
OVCAR-5	2.26	1.05	3.42	1.98	2.03	0.54
OVCAR-8	2.21	1.14	2.39	1.39	1.53	0.47
NCI/ADR-RES	2.04	1.47	8.31	1.35	1.52	0.27
SK-OV-3	1.78	1.75	2.35	2.34	1.86	1.63
**Renal**						
786-0	7.88	3.69	8.48	2.01	2.11	2.82
A498	2.04	1.35	2.96	6.69	4.61	0.81
ACHN	3.34	2.35	4.03	1.72	1.78	1.11
CAKI-1	2.40	1.80	2.50	1.71	1.76	0.65
RXF 393	1.84	1.61	2.38	1.55	1.76	0.52
SN12C	4.77	2.58	4.64	1.93	2.03	1.98
TK-10	3.92	2.27	4.54	2.29	2.70	0.72
UO-31	3.00	1.55	3.95	1.11	1.20	0.53
**Prostate**						
PC-3	2.49	3.03	3.73	2.33	2.83	1.45
DU-145	2.62	1.37	3.18	1.82	2.02	1.22
**Breast**						
MCF7	3.63	2.16	2.41	2.43	2.33	1.05
MDA-MB-231/ATCC	2.27	2.35	3.71	1.89	2.14	2.18
HS 578T	2.38	2.37	2.58	2.46	2.44	2.16
BT-549	6.28	4.53	3.15	3.05	2.63	2.30
T-47D	2.04	1.66	ND	1.53	1.88	0.58
MDA-MB-468	5.62	3.24	1.05	1.80	1.72	0.36
**MID ^a^**	3.06	2.15	3.65	1.89	1.96	0.95

^a^ The average of the total GI_50_ values.

**Table 3:** Enzymatic inhibition activity of compounds **6a**–**c**, **7b,** and **9b** against HDACs 1,4, and 6 versus **TSA** (IC_50_, µM).

	HDAC1	HDAC4	HDAC6
**6a**	3.28 ± 0.53	4.41 ± 0.30	3.11 ± 0.52
**6b**	4.27 ± 0.67	6.93 ± 1.65	3.46 ± 0.17
**6c**	4.15 ± 0.74	5.99 ± 0.11	11.72 ± 1.25
**7a**	0.37 ± 0.04	0.58 ± 0.05	0.70 ± 0.10
**9b**	2.16 ± 0.20	0.49 ± 0.08	1.83 ± 0.27
**TSA**	0.0036 ± 0.0007	0.009 ± 0.0002	0.0095 ± 0.002

**Table 4:** Swiss ADME physicochemical and pharmacokinetic parameters of SAHA and final compounds.

Molecule	6a	6b	6c	7a	7b	7c	9a	9b	9c	SAHA
MW	367.89	381.92	403.93	381.92	395.95	417.95	399.89	413.92	435.93	264.32
Fraction Csp3	0.53	0.56	0.3	0.56	0.58	0.33	0.2	0.24	0.04	0.43
No. of rotatable bonds	9	9	10	10	10	11	6	6	7	10
No. of H-bond acceptors	4	4	4	4	4	4	3	3	3	3
No. of H-bond donors	3	3	3	3	3	3	3	3	3	3
TPSA	102.49	102.49	102.49	102.49	102.49	102.49	102.49	102.49	102.49	78.43
LOGP	2.49	3.02	3.54	3.46	3.65	3.71	2.91	3.24	2.71	1.84
GI absorption	High	High	High	High	High	High	High	High	High	High
BBB permeant	No	No	No	No	No	No	No	No	No	No
Pgp substrate	Yes	Yes	Yes	Yes	Yes	Yes	No	No	No	No
Lipinski no. of violations	0	0	0	0	0	0	0	0	0	0
Bioavailability score	0.55	0.55	0.55	0.55	0.55	0.55	0.55	0.55	0.55	0.55

## Data Availability

The original contributions presented in this study are included in the article or Supplementary Materials. Further inquiries can be directed to the corresponding author.

## Supplementary Materials

The following supporting information can be downloaded at: https://www.mdpi.com/article/10.3390/ph19030442/s1, Table S1: NCI five-dose screening results of compound **6c**; Table S2: NCI five-dose screening results of compound **7a**; Table S3: NCI five-dose screening results of compound **7b**; Table S4: NCI five-dose screening results of compound **9a**; Table S5: NCI five-dose screening results of compound **9b**; Figure S1: 2D interactions between SAHA (d), compounds **6c** (a), **7b** (b), and **7c** (c) with the active site of HDLP (PDB:lZZ1); Figure S2: 2D interactions between Panobinostat and compound **9a** with the active site of HDLP (PDB:lZZ1); Figure S3: Bioavailability radar plots for compounds **6c**, **7a**–**c**, and **9a**,**c**; Figure S4: ^1^H NMR of compound **1b** (600 MHz, CDCl3); Figure S5: ^13^C NMR of compound **1b** (151 MHz, CDCl3); Figure S6: ^1^H NMR of compound **1c** (600 MHz, CDCl3); Figure S7: ^13^C NMR of compound **1c** (151 MHz, CDCl3); Figure S8. ^1^H NMR of compound **2c** (600 MHz, CDCl3); Figure S9: ^13^C NMR of compound **2c** (151 MHz, CDCl3); Figure S10. ^1^H NMR of compound **3c** (600 MHz, CDCl3); Figure S11: ^13^C NMR of compound **3c** (151 MHz, CDCl3); Figure S12: ^1^H NMR of compound **4a** (600 MHz, DMSO-*d*_6_); Figure S13: ^13^C NMR spectrum of compound **4a** (151MHz, DMSO-*d*_6_); Figure S14: ^1^H NMR of compound **4b** (600 MHz, MeOD); Figure S15: ^13^C NMR spectrum of compound **4b** (151 MHz, MeOD); Figure S16: ^1^H NMR of compound **4c** (600 MHz, DMSO-*d*_6_); Figure S17: ^13^C NMR spectrum of compound **4c** (151 MHz, DMSO-d6); Figure S18: ^1^H NMR of compound **5a** (600 MHz, DMSO-*d*_6_); Figure S19: ^13^C NMR spectrum of compound **5a** (151 MHz, DMSO-*d*_6_); Figure S20: ^1^H NMR of compound **5b** (600 MHz, MeOD); Figure S21: ^13^C NMR spectrum of compound **5b** (151 MHz, MeOD); Figure S22: ^1^H NMR of compound **5c** (600 MHz, DMSO-*d*_6_); Figure S23: ^13^C NMR spectrum of compound **5c** (151 MHz, DMSO-*d*_6_); Figure S24: ^1^H NMR of compound **6a** (600 MHz, MeOD); Figure S25: ^13^C NMR spectrum of compound **6a** (151 MHz, MeOD); Figure S26: ^1^H NMR of compound **6b** (600 MHz, MeOD); Figure S27: ^13^C NMR spectrum of compound **6b** (151 MHz, MeOD); Figure S28: ^1^H NMR of compound **6c** (600 MHz, DMSO-d6); Figure S29: ^13^C NMR spectrum of compound **6c** (151 MHz, DMSO-*d*_6_); Figure S30: ^1^H NMR of compound **7a** (600 MHz, MeOD); Figure S31: ^13^C NMR spectrum of compound **7a** (151 MHz, MeOD); Figure S32: ^1^H NMR of compound **7b** (600 MHz, DMSO-*d*_6_); Figure S33: ^13^C NMR spectrum of compound **7b** (151 MHz, DMSO-d6); Figure S34: ^1^H NMR of compound **7c** (600 MHz, MeOD); Figure S35: ^13^C NMR spectrum of compound **7c** (151 MHz, MeOD); Figure S36: ^1^H NMR of compound **8a** (600 MHz, CDCl3); Figure S37: ^13^C NMR spectrum of compound **8a** (151 MHz, CDCl3); Figure S38: ^1^H NMR of compound **8b** (600 MHz, CDCl3); Figure S39: ^13^C NMR spectrum of compound **8b** (151 MHz, CDCl3); Figure S40: ^1^H NMR of compound **8c** (600 MHz, DMSO-d6); Figure S41: ^13^C NMR spectrum of compound **8c** (151 MHz, DMSO-*d*_6_); Figure S42: 1H NMR of compound **9a** (600 MHz, DMSO-*d*_6_); Figure S43: ^13^C NMR spectrum of compound **9a** (151 MHz, DMSO-*d*_6_); Figure S44: ^1^H NMR of compound **9b** (600 MHz, DMSO-*d*_6_); Figure S45: ^13^C NMR spectrum of compound **9b** (151 MHz, DMSO-*d*_6_); Figure S46: ^1^H NMR of compound **9c** (600 MHz, DMSO-*d*_6_); Figure S47: ^13^C NMR spectrum of compound **9c** (151 MHz, DMSO-*d*_6_); Figure S48: ESI-HRMs of compound **6a**; Figure S49: ESI-HRMs of compound **6b**; Figure S50: ESI-HRMs of compound **6c**; Figure S51: ESI-HRMs of compound **7a**; Figure S52: ESI-HRMs of compound **7b**; Figure S53: ESI-HRMs of compound **7c**; Figure S54: ESI-HRMs of compound **9a**; Figure S55: ESI-HRMs of compound **9b**; Figure S56: ESI-HRMs of compound **6c**.
