# Supplementary material for: Harnessing Substituted 4-Chlorothieno[2,3-b]pyridine as a New Cap for Potent and Selective Antiproliferative HDAC Inhibitors
Source: Pharmaceuticals (Basel). 2026 Mar 9;19(3):442. doi: 10.3390/ph19030442 (PMC13029047; doi:10.3390/ph19030442)
Supplement: Supplementary file 1 [file pharmaceuticals-19-00442-s001.zip › pharmaceuticals-4178309-supplementary.pdf]

Supplementary Information

Harnessing Substituted 4-Chlorothieno[2,3-*b*]pyridine as a New Cap for Potent and Selective Antiproliferative HDAC Inhibitors

Mostafa M. Badran [1], Berkay Beyri [2], Hiroshi Tateishi [2], Kazunori Shimagaki [2], Akiko Nakata [3], Akihiro Ito [3,4], Nao Nishimura [5], Samar H. Abbas [6,7], Mohamed Abdel-Aziz [6,7], Masami Otsuka [2,8], Minoru Yoshida [3,9], Mikako Fujita [2,10], Stefan Bräse [11,*] and Mohamed O. Radwan [2,12,13,14,*]

1 Department of Medicinal Chemistry, Faculty of Pharmacy, Qena University (Formerly South Valley University), Qena 83523, Egypt; mostbadran@svu.edu.eg
2 Medicinal and Biological Chemistry Science Farm Joint Research Laboratory, Faculty of Life Sciences, Kumamoto University, 5-1 Oe-honmachi, Chuo-ku, Kumamoto 862-0973, Japan; 243y2051@st.kumamoto-u.ac.jp (B.B.); htateishi@kumamoto-u.ac.jp (H.T.); kshimagaki@kumamoto-u.ac.jp (K.S.); motsuka@gpo.kumamoto-u.ac.jp (M.O.); mfujita@kumamoto-u.ac.jp (M.F.)
3 Drug Discovery Seeds Development Unit, RIKEN Center for Sustainable Resource Science, 2-1 Hirosawa, Wako, Saitama 351-0198, Japan; anakata@riken.jp (A.N.); aito@toyaku.ac.jp (A.I.); yoshidam@riken.jp (M.Y.)
4 Laboratory of Cell Signaling, School of Life Sciences, Tokyo University of Pharmacy and Life Sciences, Hachioji, Tokyo 192-0392, Japan
5 Department of Hematology, Rheumatology and Infectious Diseases, Kumamoto University Hospital, 1-1-1 Honjo, Chuo-ku, Kumamoto 860-8556, Japan; naonishimura.kmuv@gmail.com
6 Medicinal Chemistry Department, Faculty of Pharmacy, Minia University, Minia 61519, Egypt; samar_hafez@mu.edu.eg (S.H.A.); abulnil@mu.edu.eg (M.A.-A.)
7 Medicinal Chemistry Department, Faculty of Pharmacy, Minia National University, New Minia 61768, Egypt
8 Department of Drug Discovery, Science Farm Ltd., 1-7-30 Kuhonji, Chuo-ku, Kumamoto 862-0976, Japan
9 Office of University Professors, The University of Tokyo, 1-1-1 Yayoi, Bunkyo-ku, Tokyo 113-8657, Japan
10 Department of Molecular Biology and Genetics, Burdur Mehmet Akif, Ersoy University, Degirmenler Mahallesi-Istiklal Yerleskesi, Yakakoy, Merkez, Burdur 15200, Turkey
11 Institute of Biological and Chemical Systems—Functional Molecular Systems (IBCS-FMS), Karlsruhe Institute of Technology (KIT), Kaisersrasse 12, 76131 Karlsruhe, Germany
12 UF Genetics Institute, University of Florida, Gainesville, FL 32610, USA
13 Department of Cellular and Systems Pharmacology, College of Pharmacy, University of Florida, Gainesville, FL 32610, USA
14 Department of Medicinal Chemistry, College of Pharmacy, University of Florida, Gainesville, FL 32610, USA
* Correspondence: stefan.braese@kit.edu (S.B.); radwanm@ufl.edu (M.O.R.)

Table S1. NCI five-dose screening results of compound **6c** (μM).

panel	cell line	GI_{50}	MID^b	MID^a/MID^b	TGI	LC_{50}
Leukemia	CCRF-CEM	1.53	1.68	1.82	18.4	>65
	HL-60(TB)	1.38			3.48	>65
	K-562	2.04			10.2	>65
	MOLT-4	1.75			9.53	>65
	RPMI-8226	2.03			12.4	>65
	SR	1.37			>65	>65
Non-Small Cell Lung	A549/ATCC	3.2	3.89	0.79	12.8	36.5
	EKVX	4.58			14	35.2
	HOP-62	2.73			10.7	30.5
	HOP-92	1.59			5.64	24.5
	NCI-H226	8.97			18.5	38.3
	NCI-H23	5.26			14.4	34.8
	NCI-H322M	3.57			12.4	32.8
	NCI-H460	2.9			12	35.7
	NCI-H522	2.17			10.6	36.6
Colon	COLO 205	2.2	2.85	1.08	7.96	29.9
	HCC-2998	4.17			12.5	29.6
	HCT-116	2.5			9.17	26.4
	HCT-15	3.72			12.6	31.2
	HT29	2.16			11.7	>65
	KM12	3.59			12.2	29.8
	SW-620	1.59			4.95	24.2
Central Nervous System	SF-268	3.19	3.51	0.87	11.3	31.4
	SF-295	4.6			13.2	30.6
	SF-539	3.03			11.2	28.4
	SNB-19	5.48			13.9	31.4
	SNB-75	2.25			12.9	>65
	U251	2.49			10.9	30
Melanoma	LOX IMVI	2.24	2.52	1.22	8.3	26.5
	MALME-3M	1.31			8.1	27.3
	M14	3.48			13.6	37.9
	MDA-MB-435	2.43			9.09	25.2
	SK-MEL-2	2.48			9.99	28.8
	SK-MEL-28	2.77			10.1	26.1
	SK-MEL-5	3.39			11.2	27.3
	UACC-257	2.14			11.2	31.8
	UACC-62	2.45			9.95	27.1
Ovarian	IGROV1	3.88	2.66	1.15	15.2	50.9
	OVCAR-3	2.77			11.2	30.8
	OVCAR-4	3.66			14.1	39.8
	OVCAR-5	2.26			9.31	26.3
	OVCAR-8	2.21			9.59	25.5
	NCI/ADR-RES	2.04			8.34	34.1
	SK-OV-3	1.78			5.16	19.7
Renal	786-0	7.88	3.65	0.84	16.6	34.9
	A498	2.04			8.93	25.2
	ACHN	3.34			11.7	28.9
	CAKI-1	2.4			10.2	26.6

	RXF 393	1.84			6.51	22.4
	SN12C	4.77			13.8	32.4
	TK-10	3.92			12.6	30.3
	UO-31	3			11.6	29.5
Pros-tate	PC-3	2.49	2.56	1.20	15.3	>65
	DU-145	2.62			13.9	>65
Breast	MCF7	3.63	3.70	0.83	12.7	32.7
	MDA-MB-231/ATCC	2.27			8.52	25.2
	HS 578T	2.38			14.4	64.4
	BT-549	6.28			15.1	35.3
	T-47D	2.04			10.4	>65
	MDA-MB-468	5.62			14.4	33.9
	MID[a]			3.06		

Table S2. NCI five-dose screening results of compound **7a** (μM).

panel	cell line	GI_{50}	MID[b]	MID[a]/MID[b]	TGI	LC_{50}
Leukemia	CCRF-CEM	0.978	1.31	1.63	>100	>100
	HL-60(TB)	2.19			14.9	>100
	K-562	1.49			>100	>100
	MOLT-4	1.34			>100	>100
	RPMI-8226	1.32			37.1	>100
	SR	0.562			>100	>100
Non-Small Cell Lung	A549/ATCC	2.05	3.23	0.66	11.6	>100
	EKVX	3.28			20	>100
	HOP-62	2.77			12.6	43.2
	HOP-92	2.07			9.94	59.9
	NCI-H226	9.45			22.9	53
	NCI-H23	3.24			15	43.6
	NCI-H322M	2.08			13.4	56.4
	NCI-H460	1.96			12.5	48.6
	NCI-H522	2.2			14.1	48.3
Colon	COLO 205	1.5	1.89	1.14	2.88	5.53
	HCC-2998	2.64			11.8	35
	HCT-116	1.29			5.13	27
	HCT-15	2.82			13.9	42.7
	HT29	1.73			15.6	>100
	KM12	1.95			9.68	31.7
	SW-620	1.29			3.73	13.1
Central Nervous System	SF-268	2.05	2.19	0.98	7.45	36.9
	SF-295	2.03			5.07	16.9
	SF-539	2.25			13.6	39.1
	SNB-19	3			14.9	51.8
	SNB-75	1.94			14.2	>100
	U251	1.89			6.93	28.8
Melanoma	LOX IMVI	2.27	1.77	1.21	7.58	29.3
	MALME-3M	0.613			10.3	36.9
	M14	2.4			12.9	49.1
	MDA-MB-435	1.27			3.65	11.1
	SK-MEL-2	2.4			8.69	31.5

	SK-MEL-28	2.83			14.4	38.8
	SK-MEL-5	1.83			3.73	7.58
	UACC-257	0.861			10.6	37.7
	UACC-62	1.48			4.96	20.9
Ovarian	IGROV1	2.22	1.66	1.30	10.3	53.6
	OVCAR-3	1.41			6.71	27.9
	OVCAR-4	2.56			14	45.4
	OVCAR-5	1.05			5.31	43.3
	OVCAR-8	1.14			10.1	32.8
	NCI/ADR-RES	1.47			11.4	67.7
	SK-OV-3	1.75			4.5	14.5

Table S2. (continued). NCI five-dose screening results of compound **7a** (μM).

Renal	786-0	3.69	2.15	1.00	14.7	45
	A498	1.35			5.82	26.6
	ACHN	2.35			10.1	34.1
	CAKI-1	1.8			9.56	32.6
	RXF 393	1.61			4.89	18.8
	SN12C	2.58			11.6	65.2
	TK-10	2.27			11.3	37.6
	UO-31	1.55			11.3	35.2
Pros-tate	PC-3	3.03	2.2	0.98	31.8	>100
	DU-145	1.37			6.7	59.7
Breast	MCF7	2.16	2.72	0.79	14.3	>100
	MDA-MB-231/ATCC	2.35			6.85	27.7
	HS 578T	2.37			15.1	>100
	BT-549	4.53			19.8	78.8
	T-47D	1.66			6.95	>100
	MDA-MB-468	3.24			15.3	43
	MID[a]			2.15		

Table S3. NCI five-dose screening results of compound **7b** (μM).

panel	cell line	GI_{50}	MID[b]	MID[a]/MID[b]	TGI	LC_{50}
Leukemia	CCRF-CEM	2.32	2.16	1.69	24.90	>100
	HL-60(TB)	2.58			5.86	32.40
	K-562	2.10			10.10	60.60
	MOLT-4	2.01			11.60	>100
	RPMI-8226	1.78			11.20	97.40
Non-Small Cell Lung	A549/ATCC	3.45	4.30	0.85	14.20	40.50
	EKVX	3.97			18.00	53.60
	HOP-62	2.98			15.10	44.20
	HOP-92	2.57			12.70	43.70
	NCI-H226	12.20			29.00	69.20
	NCI-H23	3.99			15.70	42.80
	NCI-H322M	4.74			21.20	66.50
	NCI-H460	2.79			14.90	50.50
	NCI-H522	2.05			12.90	45.10
Colon	COLO 205	2.10	3.03	1.20	6.21	33.30
	HCC-2998	2.30			11.50	34.20

	HCT-116	2.44			11.80	38.50
	HCT-15	7.26			20.30	46.60
	HT29	2.65			12.90	76.40
	KM12	2.34			11.90	36.50
	SW-620	2.10			6.19	30.40
Central Nervous System	SF-268	3.68	4.24	0.86	14.70	48.00
	SF-295	4.16			16.60	48.90
	SF-539	5.97			19.80	49.70
	SNB-19	5.92			21.60	57.40
	SNB-75	2.20			12.40	42.00
	U251	3.49			14.50	40.60
Melanoma	LOX IMVI	1.71	3.37	1.08	3.54	7.34
	MALME-3M	4.59			20.50	59.90
	M14	4.46			18.00	49.10
	MDA-MB-435	2.33			11.50	36.20
	SK-MEL-2	3.47			13.20	41.70
	SK-MEL-28	6.79			22.10	59.90
	SK-MEL-5	1.97			6.30	23.80
	UACC-257	1.65			12.90	40.80
Ovarian	IGROV1	5.03	4.49	1.23	22.00	92.00
	OVCAR-3	3.70			15.20	41.30
	OVCAR-4	6.25			22.10	58.80
	OVCAR-5	3.42			18.10	>100
	OVCAR-8	2.39			12.30	37.70
	NCI/ADR-RES	8.31			29.30	92.80
	SK-OV-3	2.35			12.30	37.80
Renal	786-0	8.48	3.57	1.02	24.00	61.10
	A498	2.96			14.70	74.70
	ACHN	4.03			16.10	54.90
	CAKI-1	2.50			9.77	34.20
	RXF 393	2.38			8.26	34.70
	SN12C	4.64			20.70	71.80
	TK-10	4.54			17.80	45.80
	UO-31	3.95			16.90	46.40
Prostate	PC-3	3.73	3.46	1.06	21.30	82.80
	DU-145	3.18			16.20	69.00
Breast	MCF7	2.41	2.58	1.41	13.40	41.90
	MDA-MB-231/ATCC	3.71			13.10	54.80
	HS 578T	2.58			18.60	>100
	BT-549	3.15			16.90	49.20
	MDA-MB-468	1.05			5.08	25.80
	MID[a]			3.65		

Table S4. NCI five-dose screening results of compound **9a** (μM).

panel	cell line	GI_{50}	MID[b]	MID[a]/MID[b]	TGI	LC_{50}
Leukemia	CCRF-CEM	0.61	1.47	1.28	>100	>100
	HL-60(TB)	1.99			10.90	>100
	K-562	2.73			>100	>100
	MOLT-4	1.47			13.20	>100

	RPMI-8226	1.38			22.70	>100
	SR	0.67			>100	>100
Non-Small Cell Lung	A549/ATCC	1.76	1.91	0.99	3.85	>100
	EKVX	3.01			9.14	39.10
	HOP-62	1.81			3.85	8.20
	HOP-92	2.05			5.96	35.30
	NCI-H226	1.87			4.60	25.30
	NCI-H23	1.77			3.80	8.15
	NCI-H322M	1.88			8.86	40.20
	NCI-H460	1.57			3.79	9.11
	NCI-H522	1.49			3.26	7.14
Colon	COLO 205	1.75	1.66	1.14	3.46	6.82
	HCC-2998	1.67			4.21	11.50
	HCT-116	1.84			4.89	17.70
	HCT-15	0.55			10.70	39.50
	HT29	1.89			5.06	22.90
	KM12	2.07			7.68	28.80
	SW-620	1.83			5.39	30.10
Central Nervous System	SF-268	1.72	1.72	1.10	4.25	13.60
	SF-295	1.84			3.66	7.29
	SF-539	1.72			3.47	6.99
	SNB-19	1.76			4.20	10.10
	SNB-75	1.76			7.58	55.20
	U251	1.51			3.00	5.93
Melanoma	LOX IMVI	1.56	1.89	1.00	3.06	5.97
	MALME-3M	1.71			5.64	27.40
	M14	1.48			3.27	7.22
	MDA-MB-435	1.82			4.44	12.40
	SK-MEL-2	1.98			4.71	13.20
	SK-MEL-28	2.89			9.48	34.40
	SK-MEL-5	1.83			3.46	6.52
	UACC-257	2.07			6.86	46.30
	UACC-62	1.7			3.45	7.00
Ovarian	IGROV1	1.65	1.71	1.11	3.81	8.79
	OVCAR-3	1.39			4.08	14.20
	OVCAR-4	1.84			8.41	>100
	OVCAR-5	1.98			4.82	15.20
	OVCAR-8	1.39			2.77	5.52
	NCI/ADR-RES	1.35			3.30	8.07
	SK-OV-3	2.34			4.20	7.54
Renal	786-0	2.01	2.38	0.79	4.33	9.33
	A498	6.69			20.30	46.50
	ACHN	1.72			4.00	9.28
	CAKI-1	1.71			4.62	15.70
	RXF 393	1.55			3.95	10.20
	SN12C	1.93			4.55	12.10
	TK-10	2.29			6.71	25.60
	UO-31	1.11			2.48	5.55
Pros-tate	PC-3	2.33	2.08	0.91	18.40	>100
	DU-145	1.82			4.31	11.60

Breast	MCF7	2.43	2.19	0.86	9.52	49.30
	MDA-MB-231/ATCC	1.89			6.78	30.90
	HS 578T	2.46			9.25	>100
	BT-549	3.05			8.67	44.30
	T-47D	1.53			6.42	>100
	MDA-MB-468	1.8			3.66	7.46
	MID[a]			1.89		

Table S5. NCI five-dose screening results of compound **9b** (μM).

panel	cell line	GI_{50}	MID[b]	MID[a]/MID[b]	TGI	LC_{50}
Leukemia	CCRF-CEM	0.78	1.64	1.19	>100	>100
	HL-60(TB)	1.92			8.18	>100
	K-562	2.92			>100	>100
	MOLT-4	1.70			10.40	>100
	RPMI-8226	1.71			67.00	>100
	SR	0.82			>100	>100
Non-Small Cell Lung	A549/ATCC	1.72	1.94	1.01	3.63	7.65
	EKVX	2.75			8.07	36.10
	HOP-62	1.88			3.87	7.97
	HOP-92	2.14			6.75	48.60
	NCI-H226	1.85			4.05	8.87
	NCI-H23	1.93			4.20	9.11
	NCI-H322M	1.96			10.10	38.00
	NCI-H460	1.71			3.86	8.74
	NCI-H522	1.54			3.38	7.42
Colon	COLO 205	1.85	1.79	1.10	3.49	6.60
	HCC-2998	1.99			4.64	12.30
	HCT-116	1.70			3.82	8.57
	HCT-15	0.69			10.40	39.40
	HT29	2.36			9.10	66.60
	KM12	2.03			7.05	27.40
	SW-620	1.88			4.92	24.20
Central Nervous System	SF-268	1.73	1.88	1.04	4.04	9.42
	SF-295	1.87			3.72	7.40
	SF-539	1.68			3.47	7.20
	SNB-19	1.89			4.59	13.40
	SNB-75	2.62			12.10	48.80
	U251	1.51			3.01	5.99
Melanoma	LOX IMVI	1.69	1.97	0.99	3.27	6.30
	MALME-3M	1.70			5.61	28.10
	M14	1.67			3.54	7.49
	MDA-MB-435	2.01			4.87	16.20
	SK-MEL-2	1.92			4.61	12.90
	SK-MEL-28	3.00			10.30	37.00
	SK-MEL-5	1.84			3.44	6.44
	UACC-257	2.15			6.87	39.30
	UACC-62	1.76			3.55	7.14
Ovarian	IGROV1	1.89	1.84	1.07	4.32	9.87
	OVCAR-3	1.72			4.56	14.80

	OVCAR-4	2.30			9.61	54.10
	OVCAR-5	2.03			5.02	16.20
	OVCAR-8	1.53			2.95	5.68
	NCI/ADR-RES	1.52			3.61	8.55
	SK-OV-3	1.86			3.65	7.15
**Renal**	**786-0**	**2.11**	**2.24**	**0.87**	**4.64**	**10.90**
	**A498**	**4.61**			**18.10**	**44.10**
	**ACHN**	**1.78**			**4.08**	**9.35**
	**CAKI-1**	**1.76**			**4.90**	**17.70**
	**RXF 393**	**1.76**			**4.31**	**11.60**
	**SN12C**	**2.03**			**4.95**	**15.30**
	**TK-10**	**2.70**			**9.38**	**31.60**
	**UO-31**	**1.20**			**2.70**	**6.09**
Pros-tate	PC-3	2.83	2.43	0.81	19.40	>100
	DU-145	2.02			4.91	19.30
Breast	MCF7	2.33	2.19	0.90	7.69	35.10
	MDA-MB-231/ATCC	2.14			7.11	29.30
	HS 578T	2.44			9.53	>100
	BT-549	2.63			7.18	28.40
	T-47D	1.88			5.49	>100
	MDA-MB-468	1.72			3.62	7.59
	MID[a]		1.96			

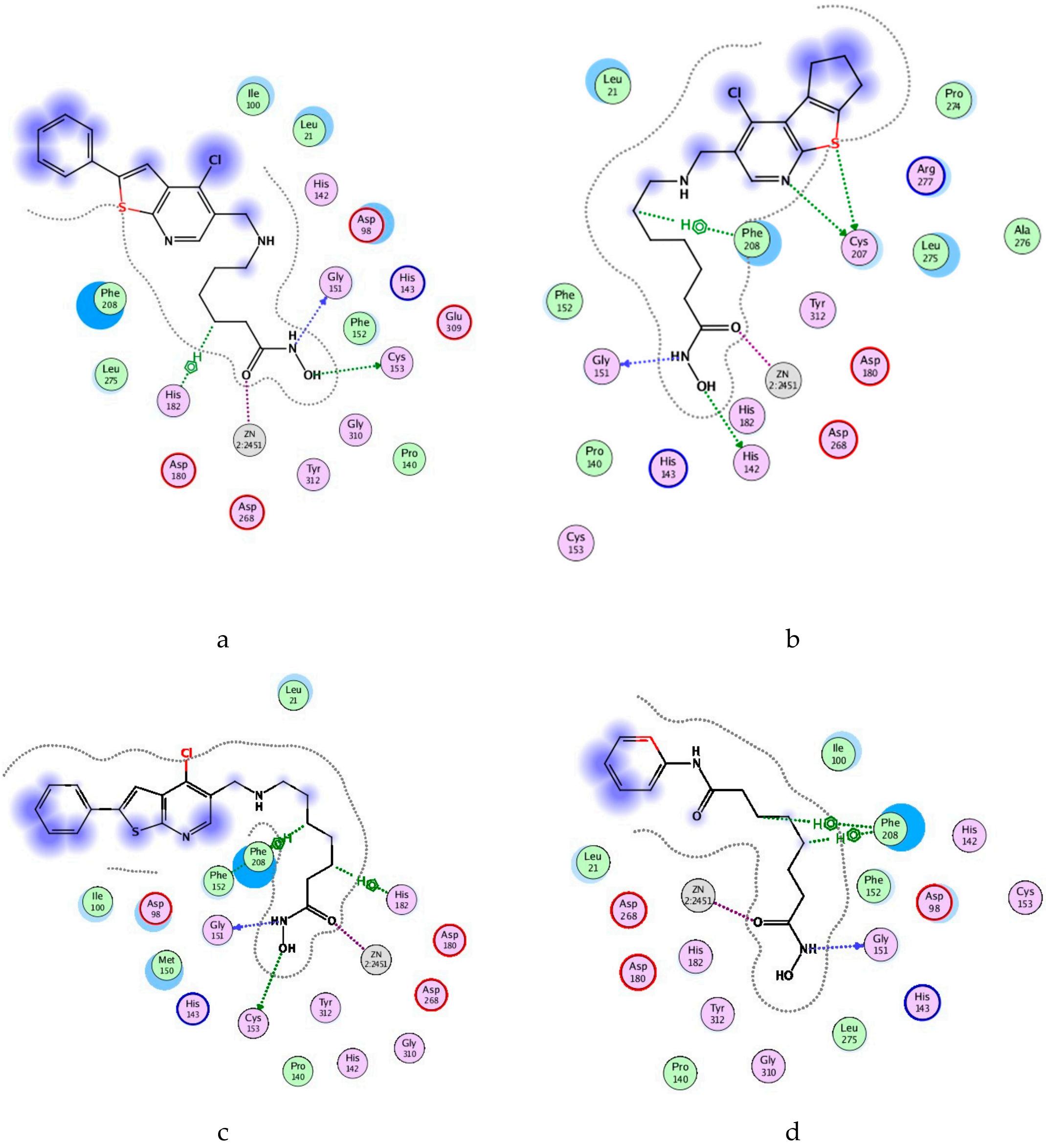

Figure S1. 2D interactions between SAHA (d), compounds **6c** (a), **7a** (b), and **7c** (c) with the active site of HDLP(PDB: lZZ1).

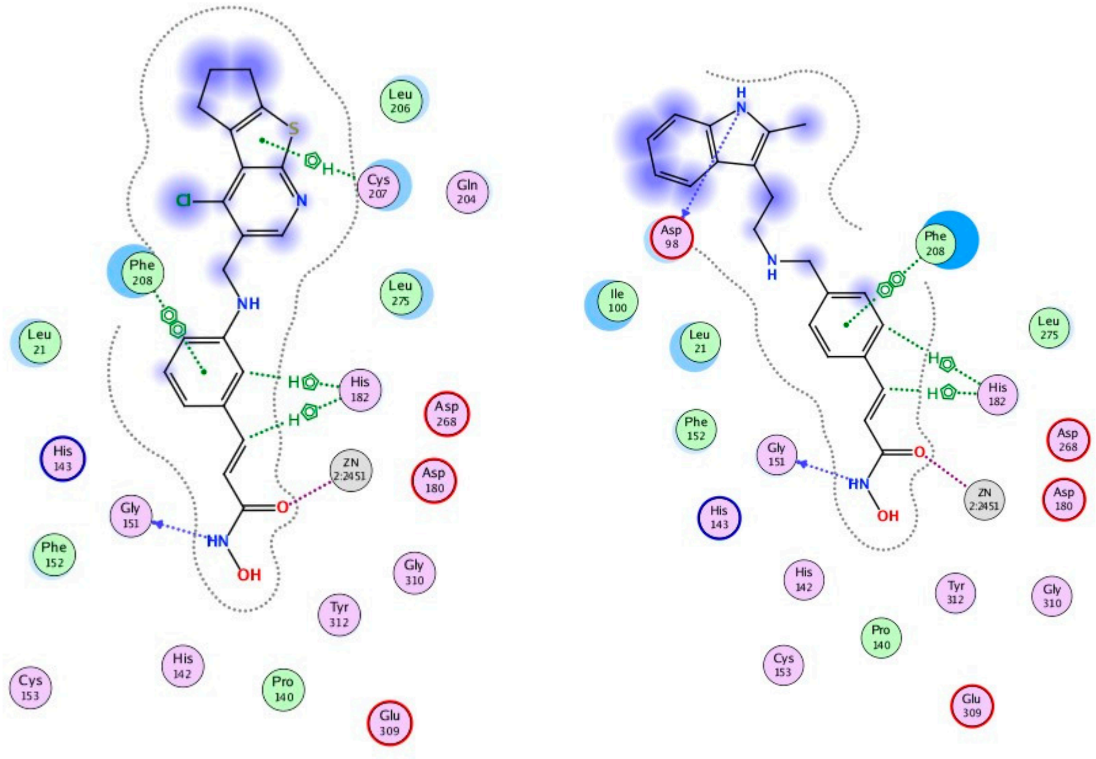

Figure S2. 2D interactions between Panobinostat (right) and compound **9a** (left) with the active site of HDLP(PDB: lZZ1).

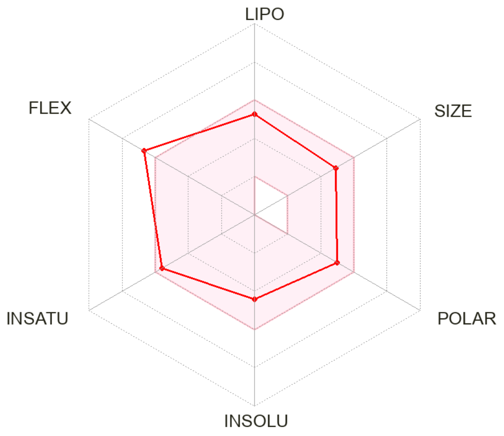

6c

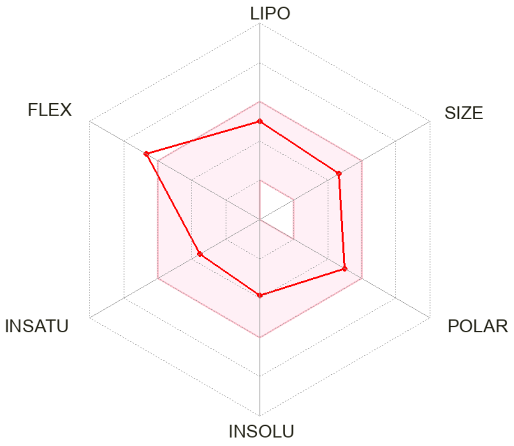

7a

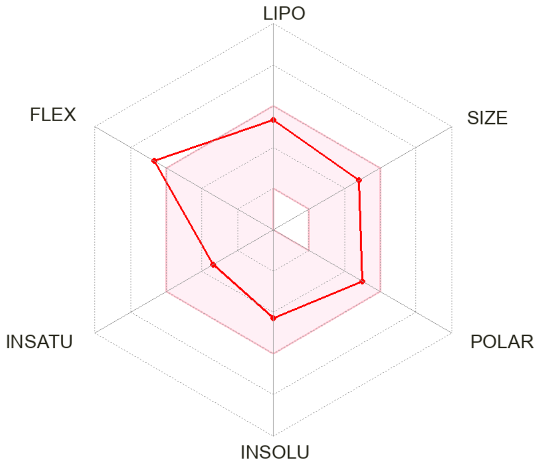

7b

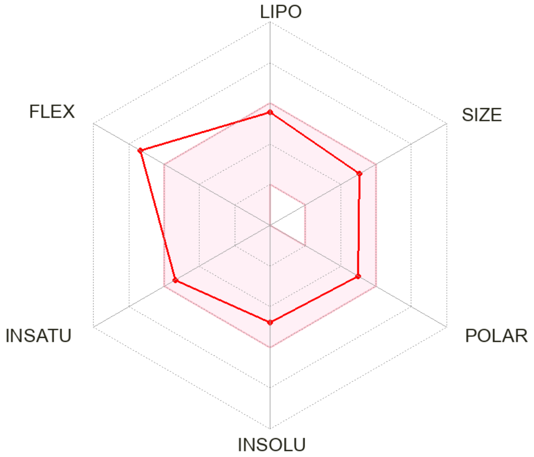

7c

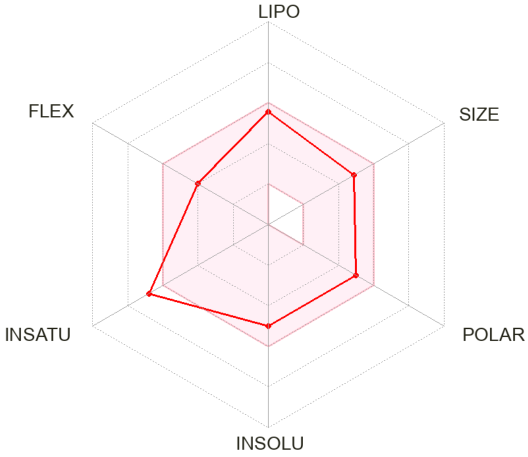

9a

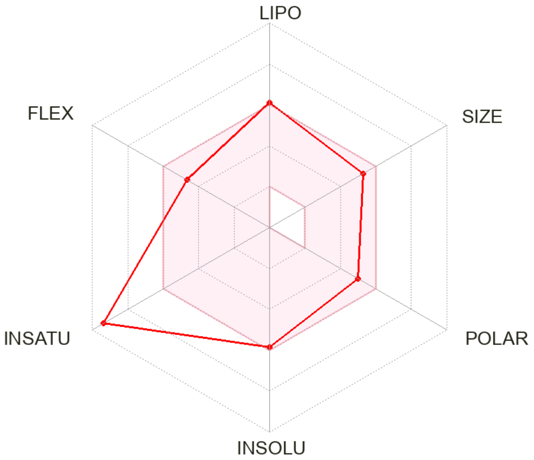

9c

Figure S3. Bioavailability radar plots for compounds **6c**, **7a-c**, and **9a,c**.

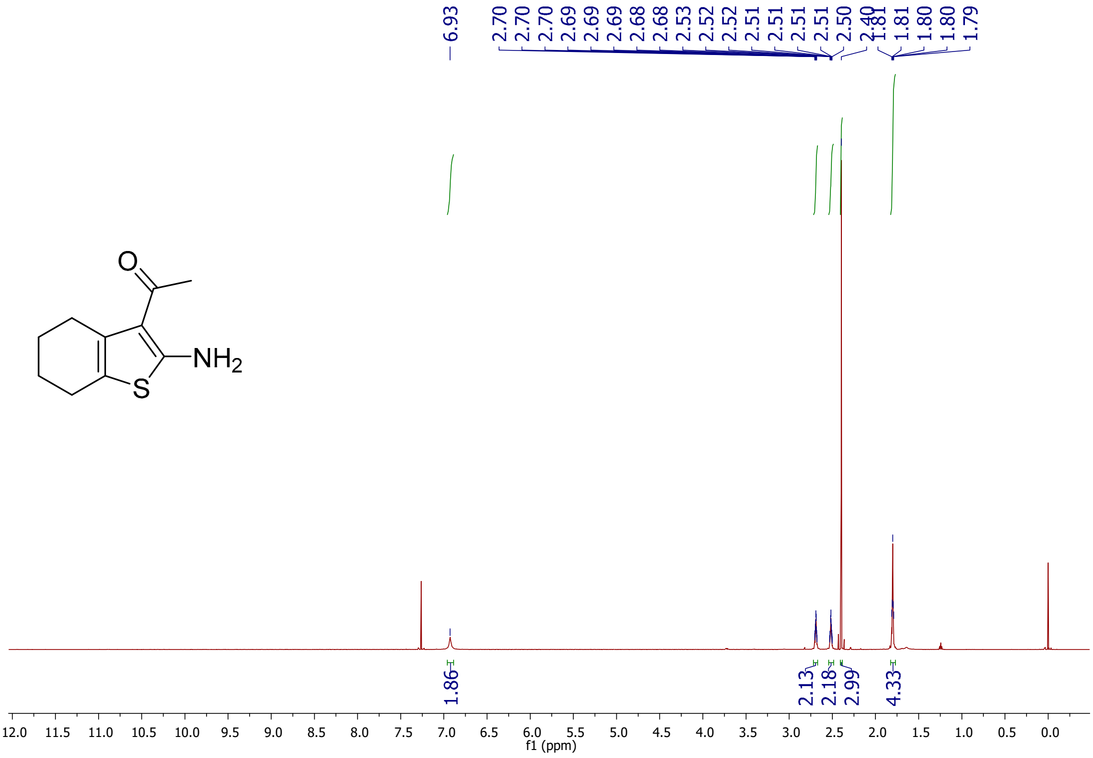

Figure S4. ^{1}H NMR of compound **1b** (600 MHz, $CDCl_3$).

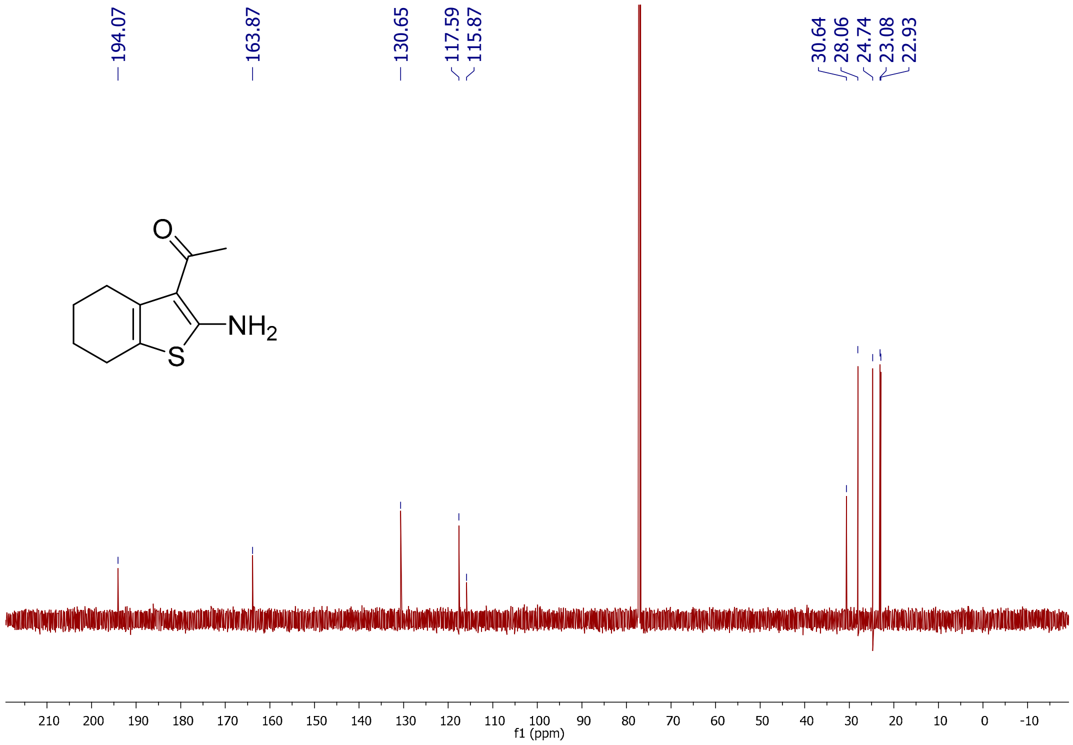

Figure S5. ^{13}C NMR of compound **1b** (151 MHz, $CDCl_3$).

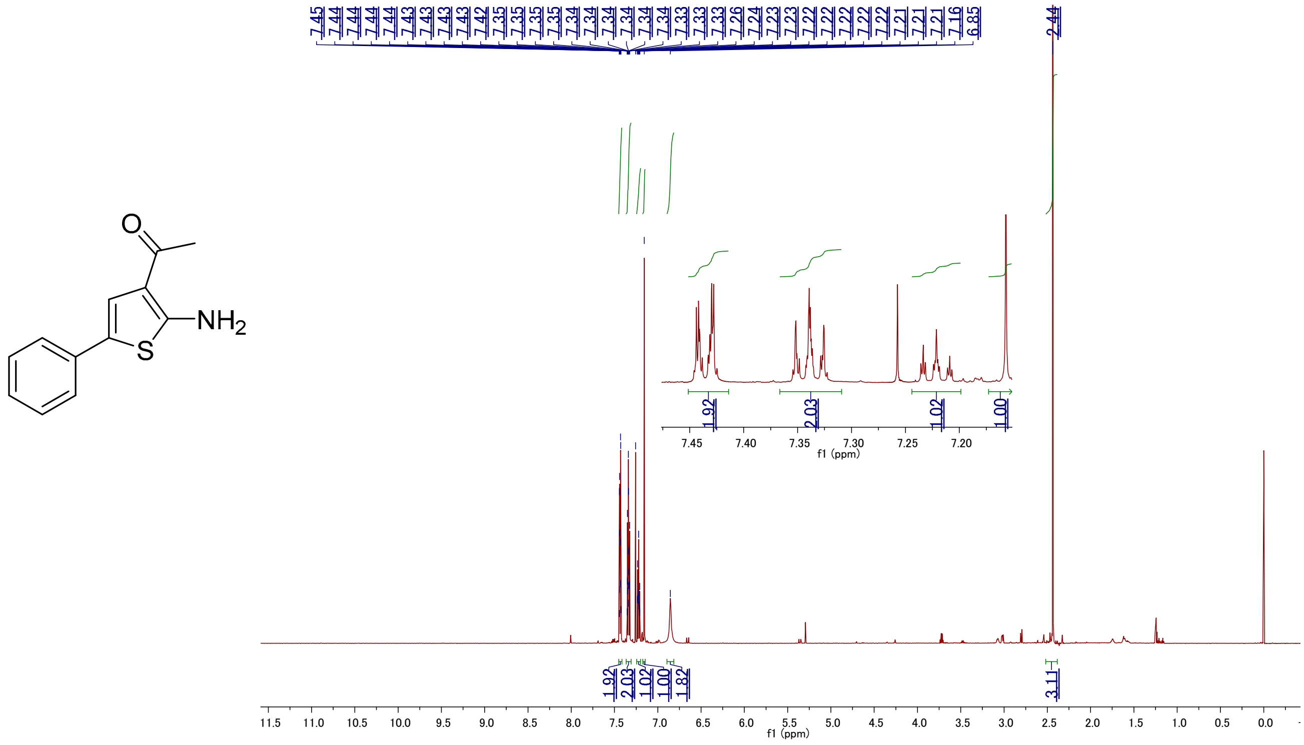

Figure S6. ^{1}H NMR of compound **1c** (600 MHz, $CDCl_3$).

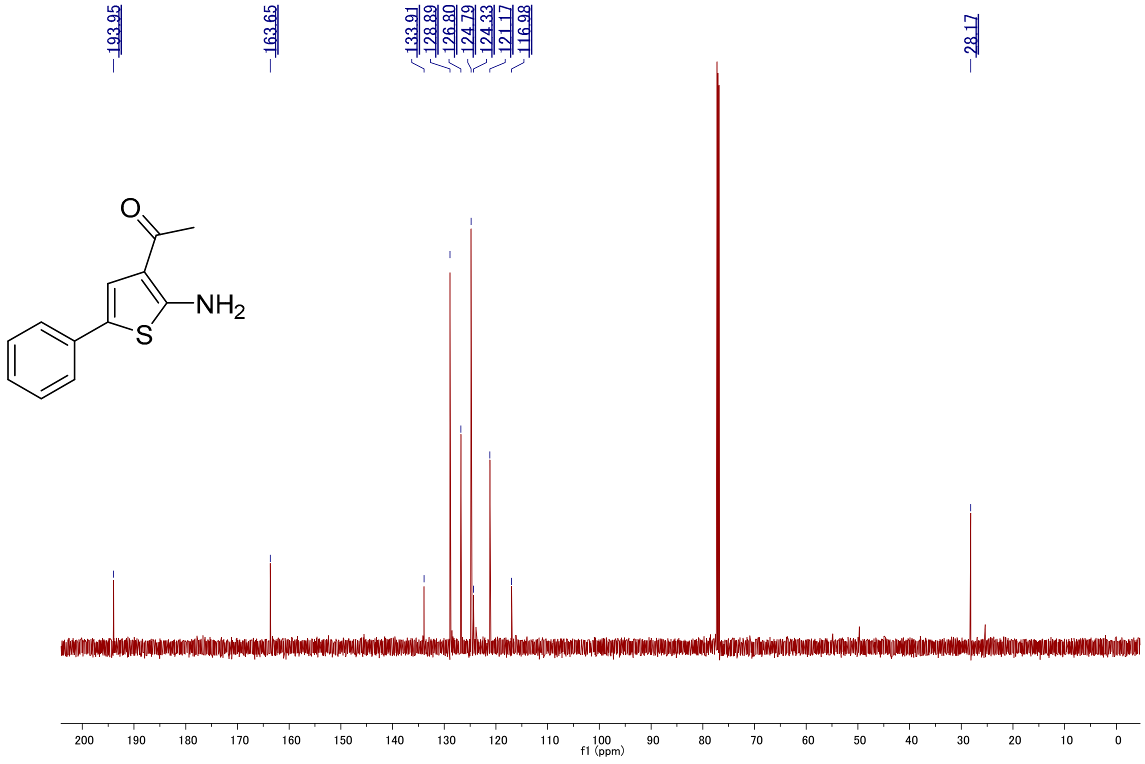

Figure S7. ^{13}C NMR of compound **1c** (151 MHz, $CDCl_3$).

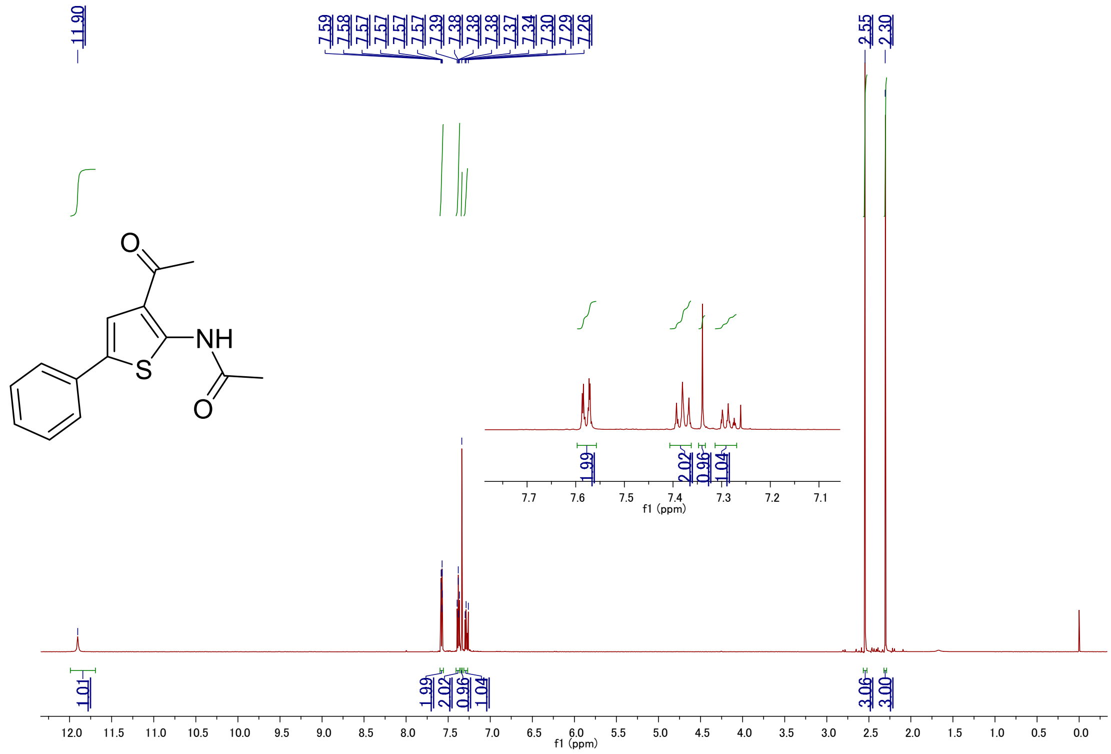

Figure S7. ^{1}H NMR of compound **2c** (600 MHz, $CDCl_3$).

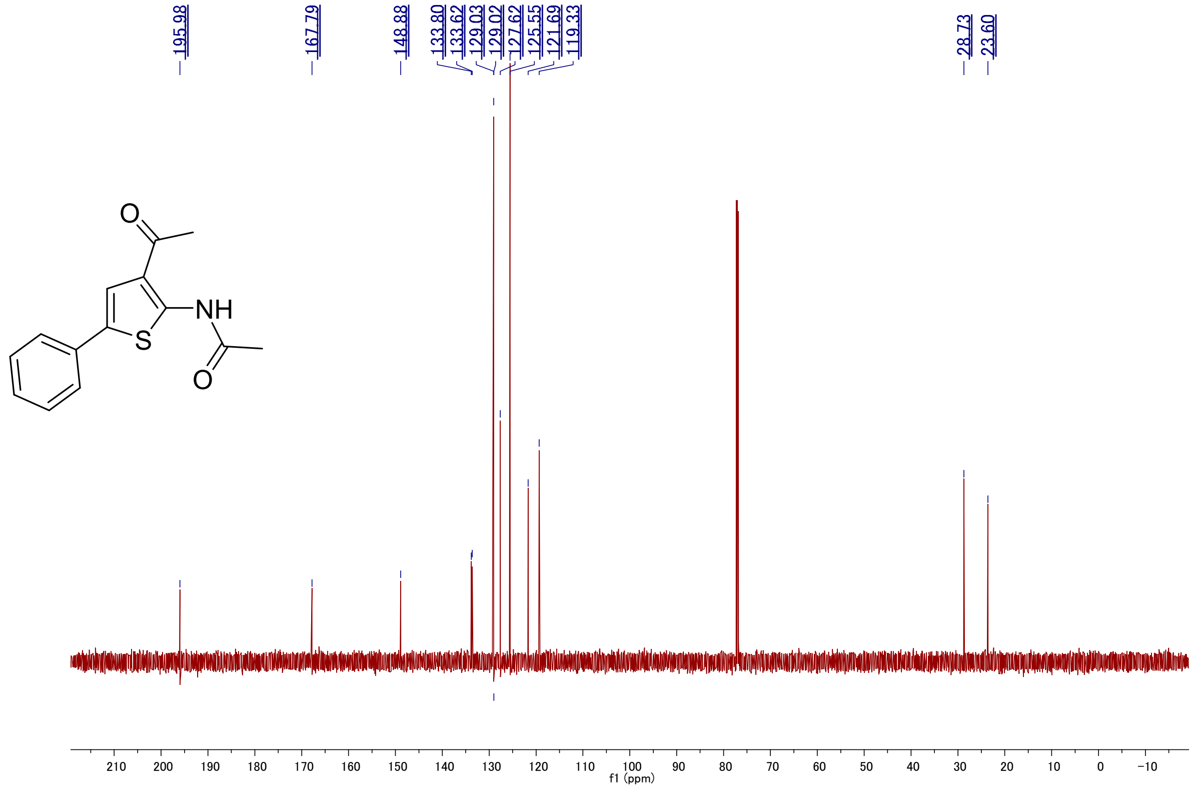

Figure S9. ^{13}C NMR of compound **2c** (151 MHz, $CDCl_3$).

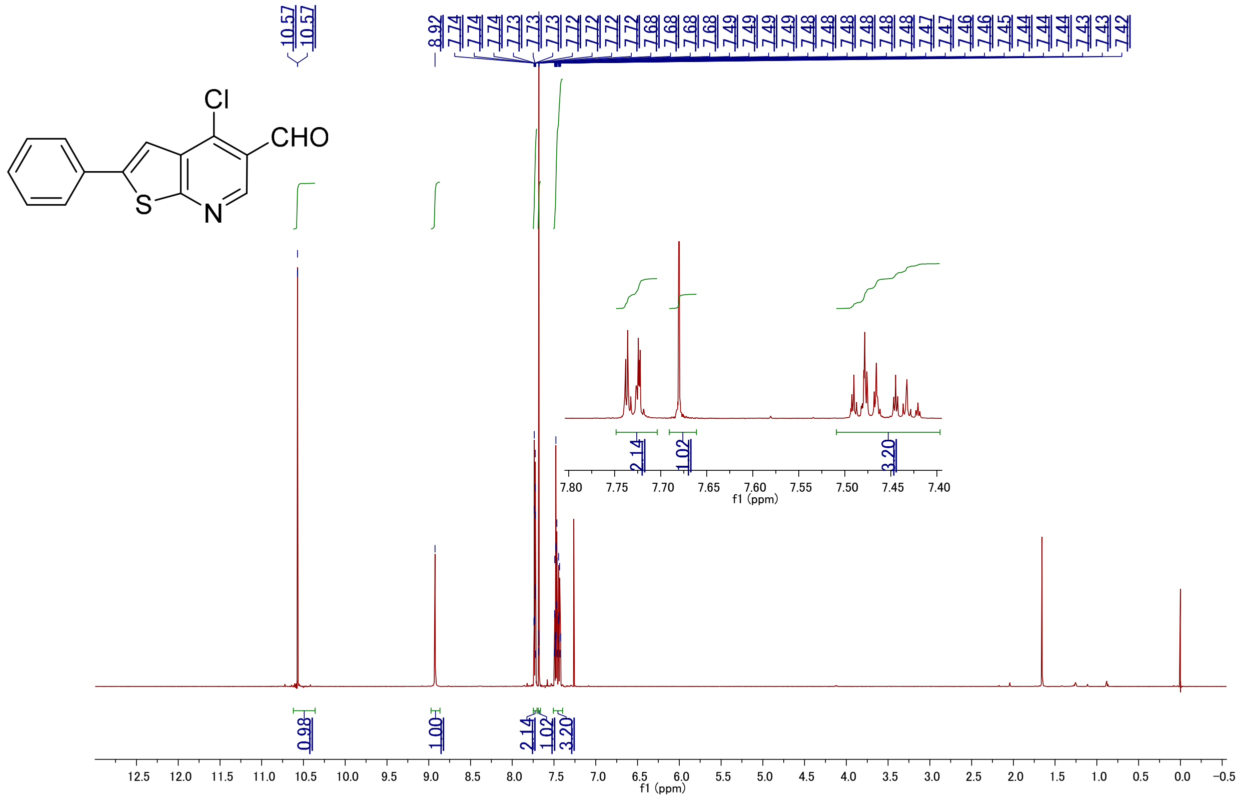

Figure S8. ^{1}H NMR of compound **3c** (600 MHz, $CDCl_3$).

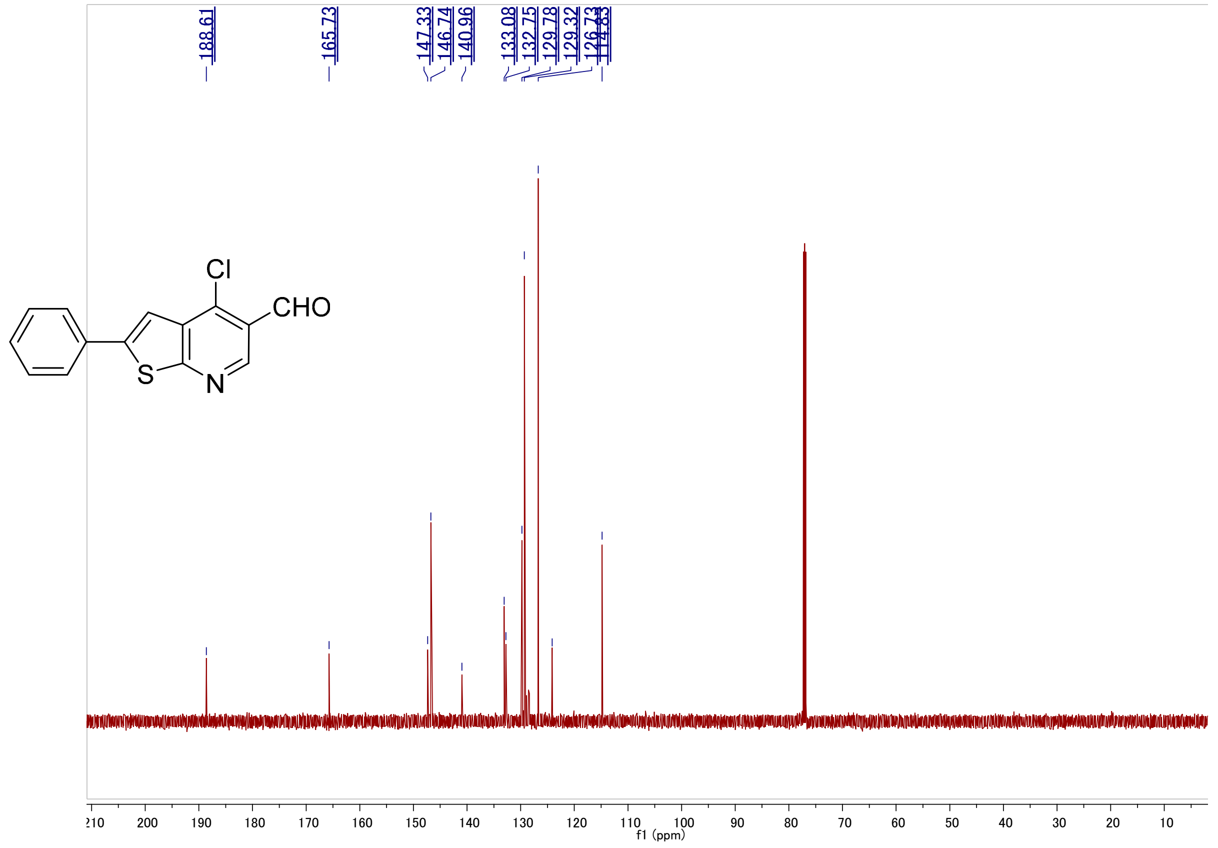

Figure S11. ^{13}C NMR of compound **3c** (151 MHz, $CDCl_3$).

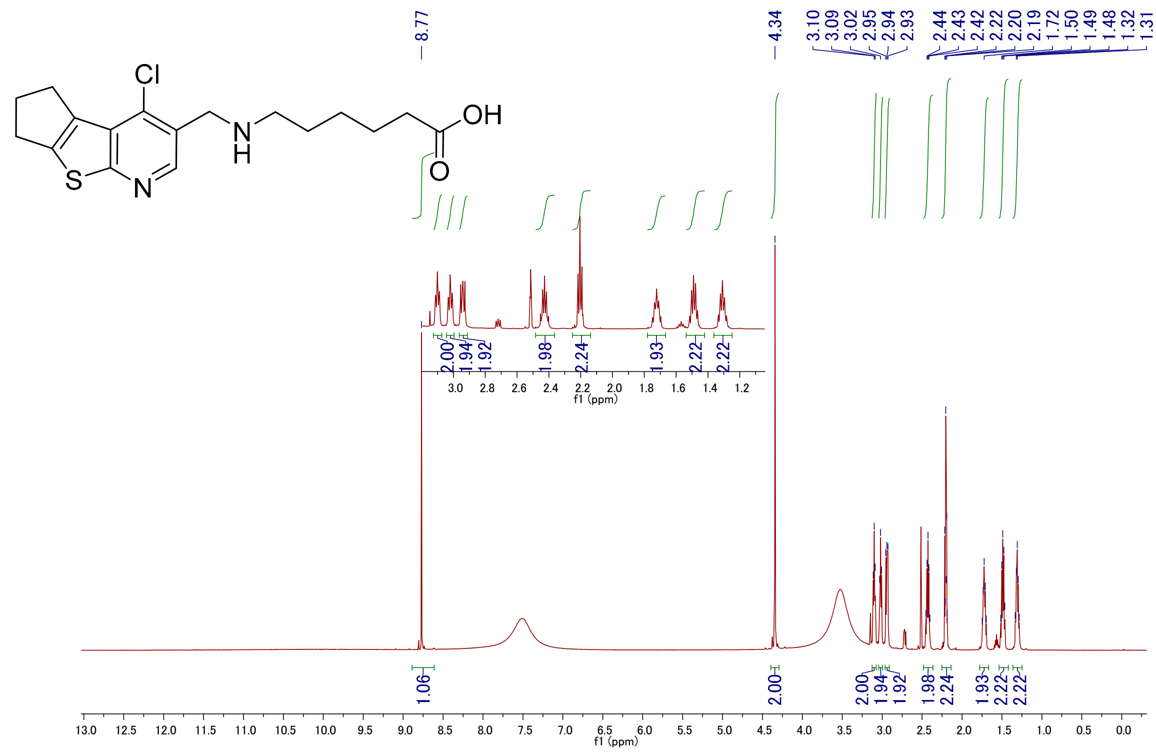

Figure S12. ^{1}H NMR of compound **4a** (600 MHz, DMSO-*d*$_6$).

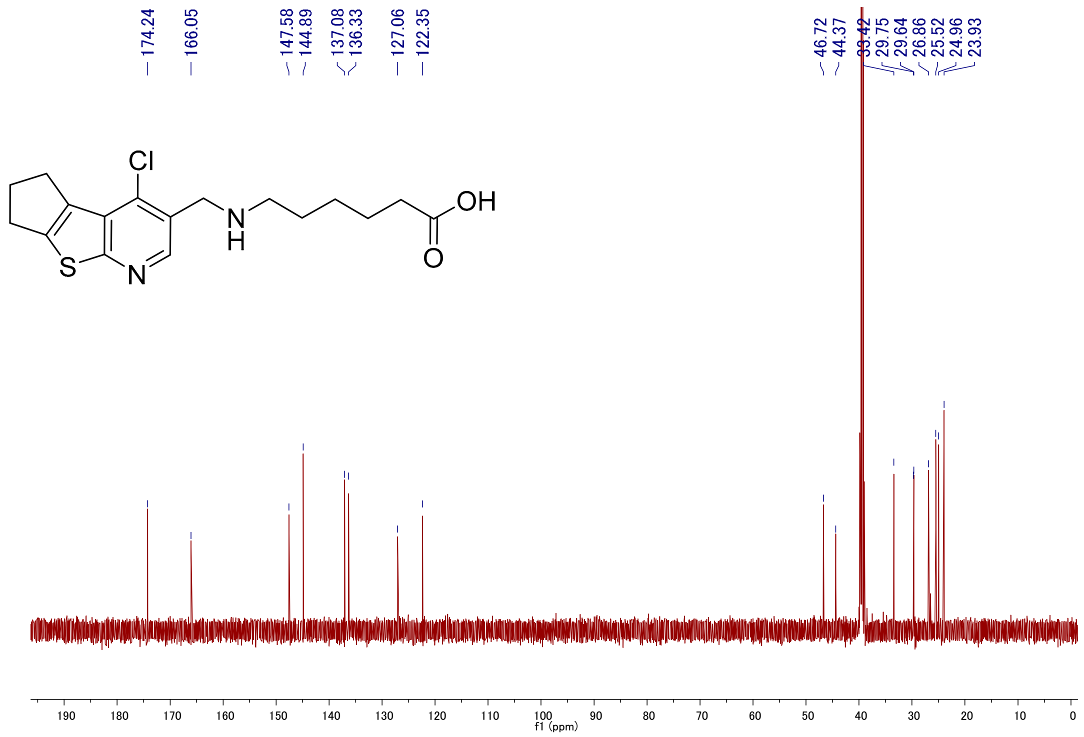

Figure S13. ^{13}C NMR spectrum of compound **4a** (151MHz, DMSO-*d*$_6$).

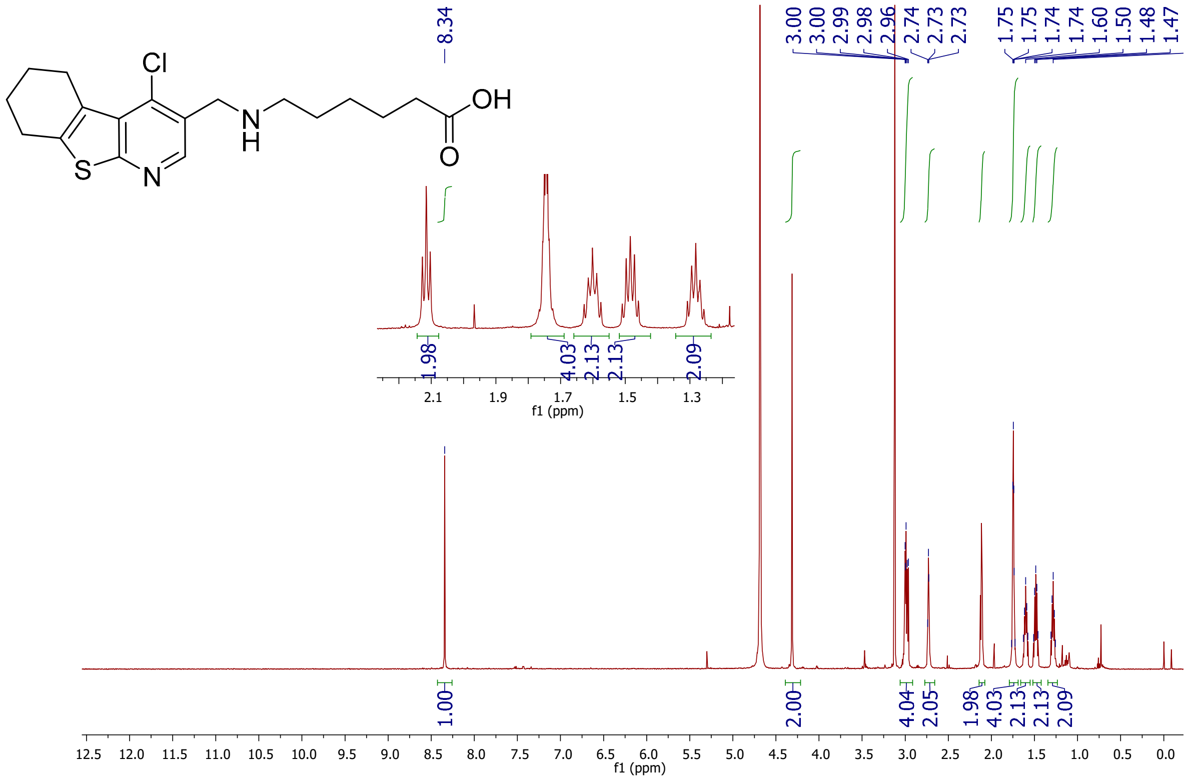

Figure S14. ^{1}H NMR of compound **4b** (600 MHz, MeOD).

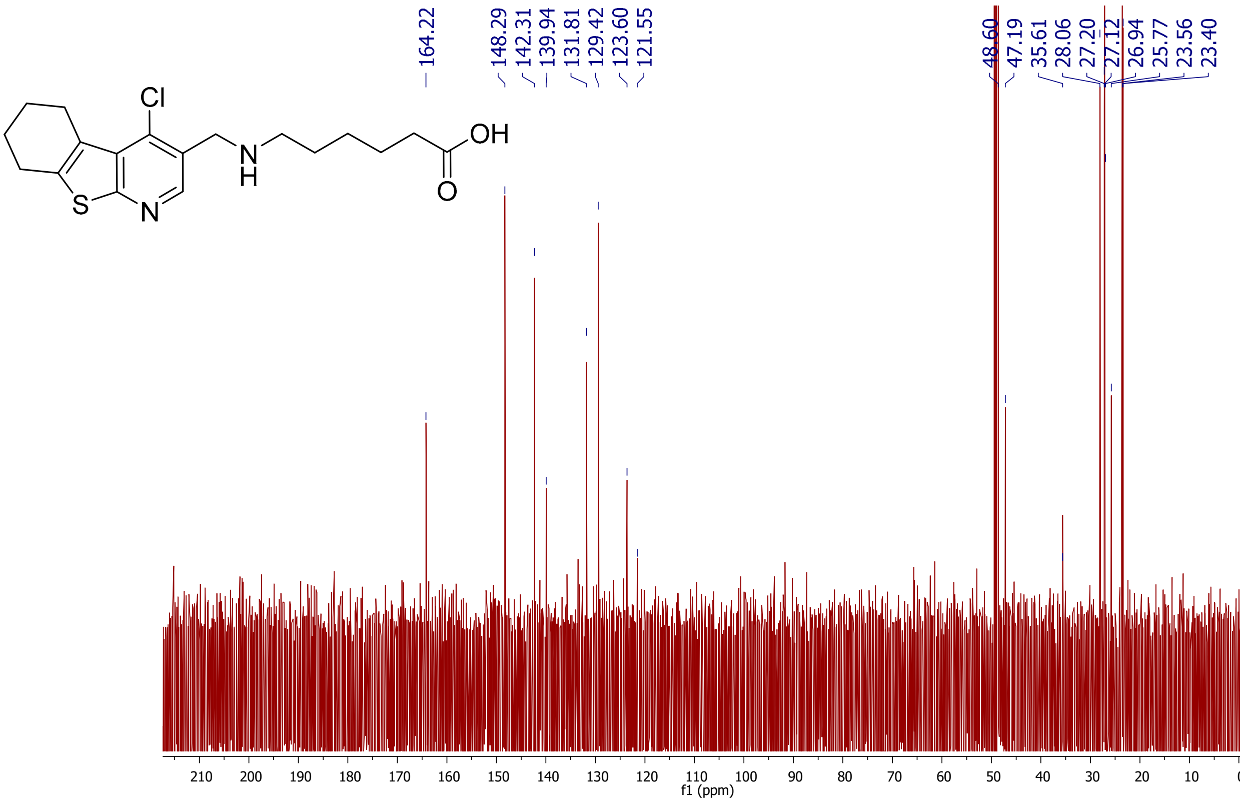

Figure S15. ^{13}C NMR spectrum of compound **4b** (151 MHz, MeOD).

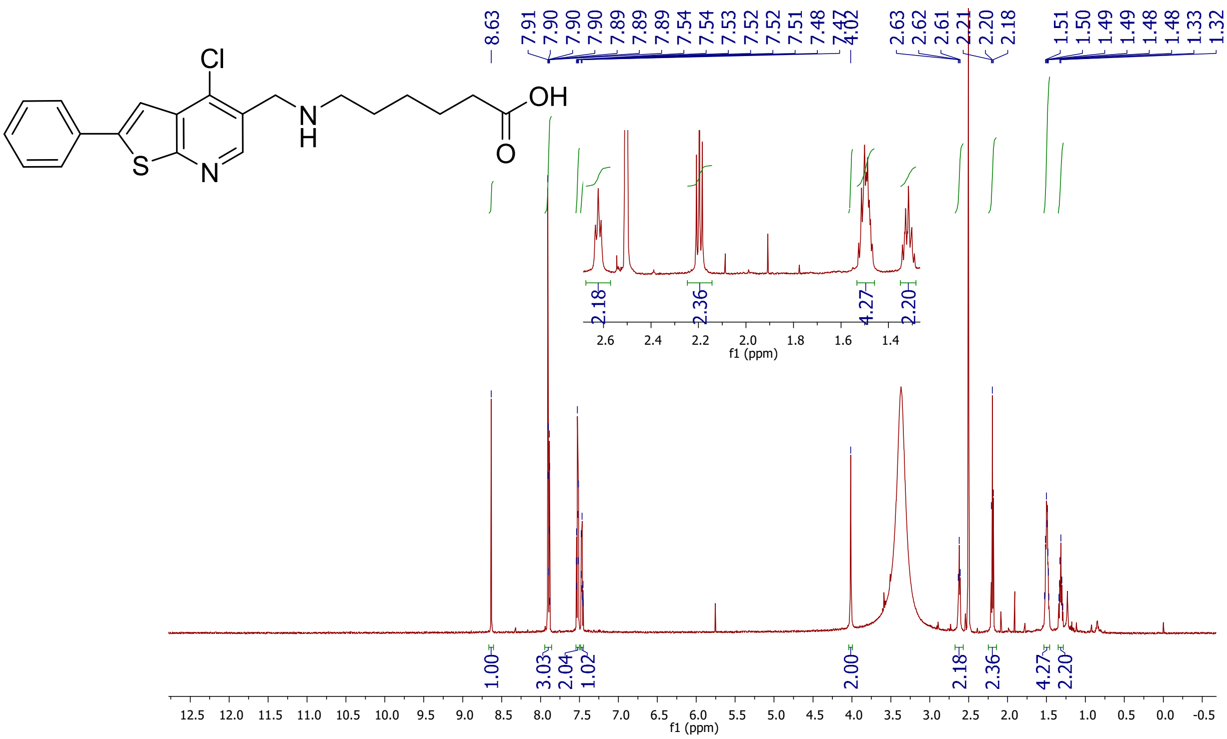

Figure S16. ^{1}H NMR of compound **4c** (600 MHz, DMSO-d_6).

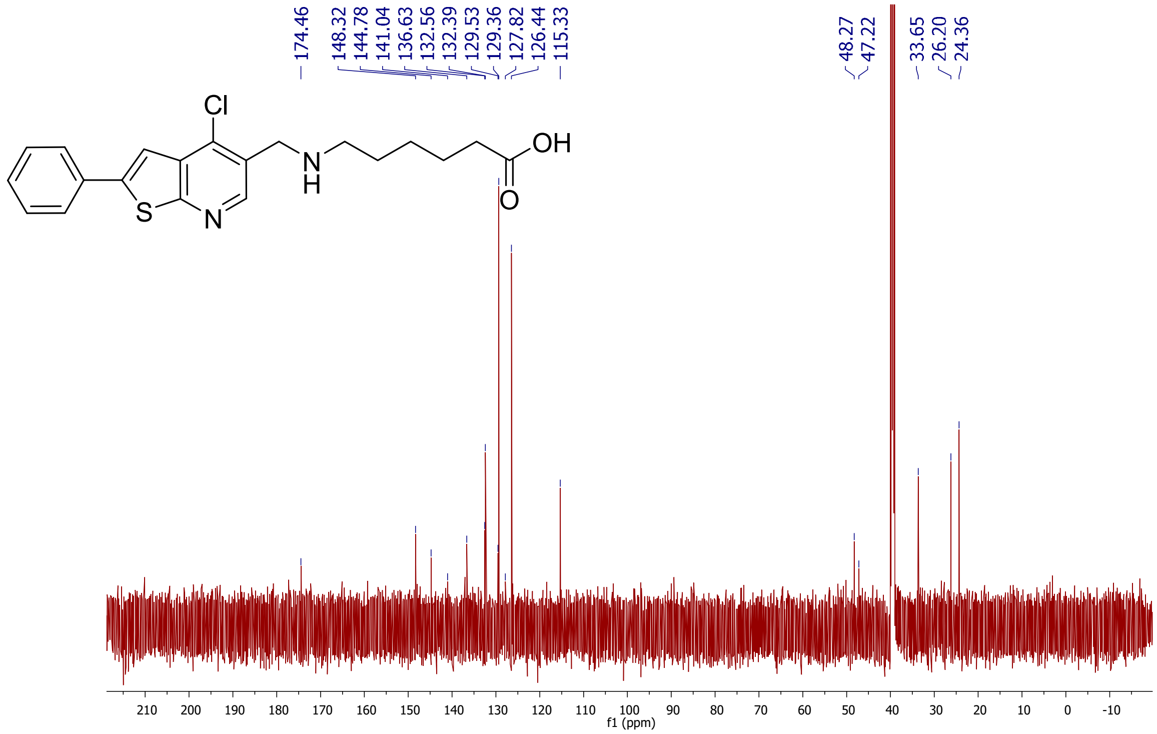

Figure S17. ^{13}C NMR spectrum of compound **4c** (151 MHz, DMSO-d_6).

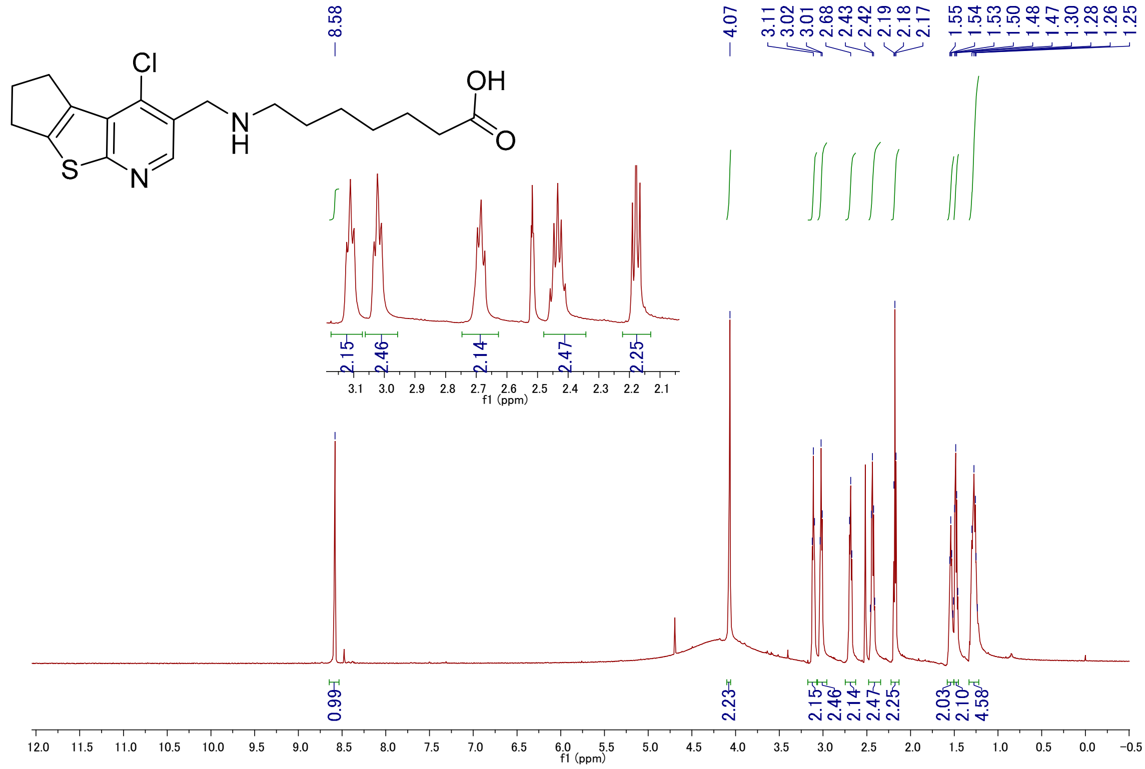

Figure S18. ^{1}H NMR of compound **5a** (600 MHz, DMSO-*d*6).

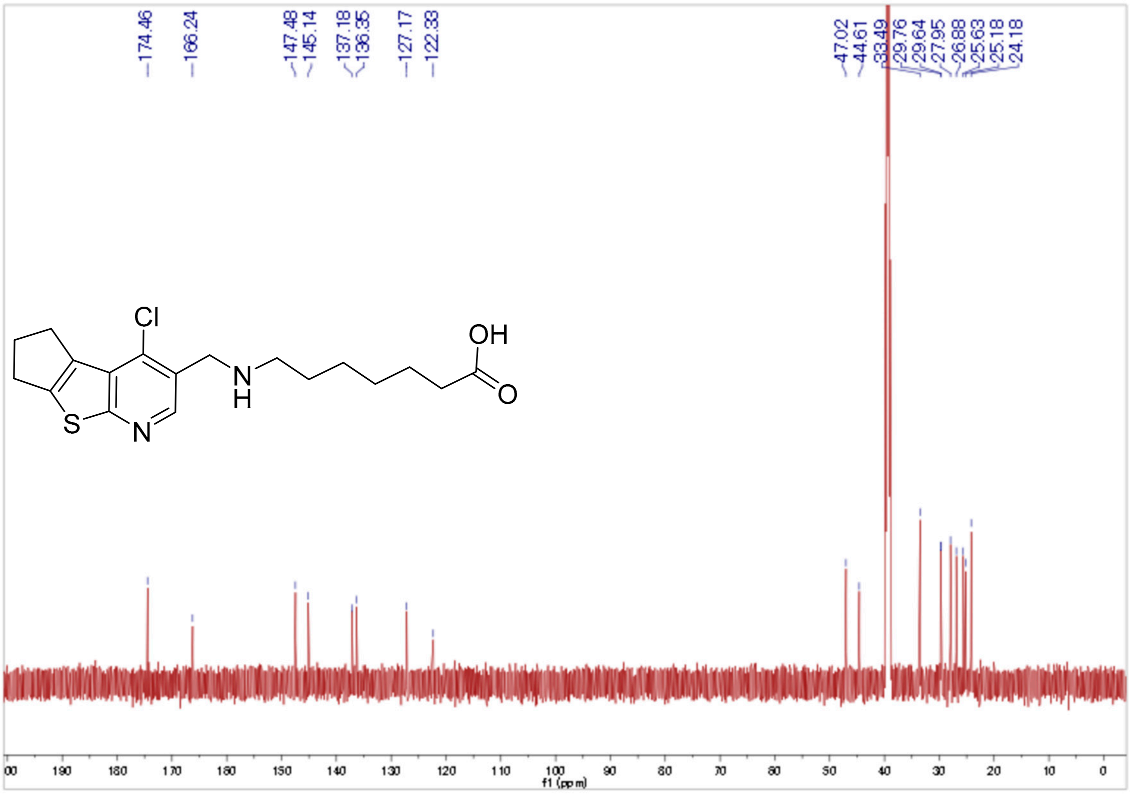

Figure S19. ^{13}C NMR spectrum of compound **5a** (151 MHz, DMSO-*d*6).

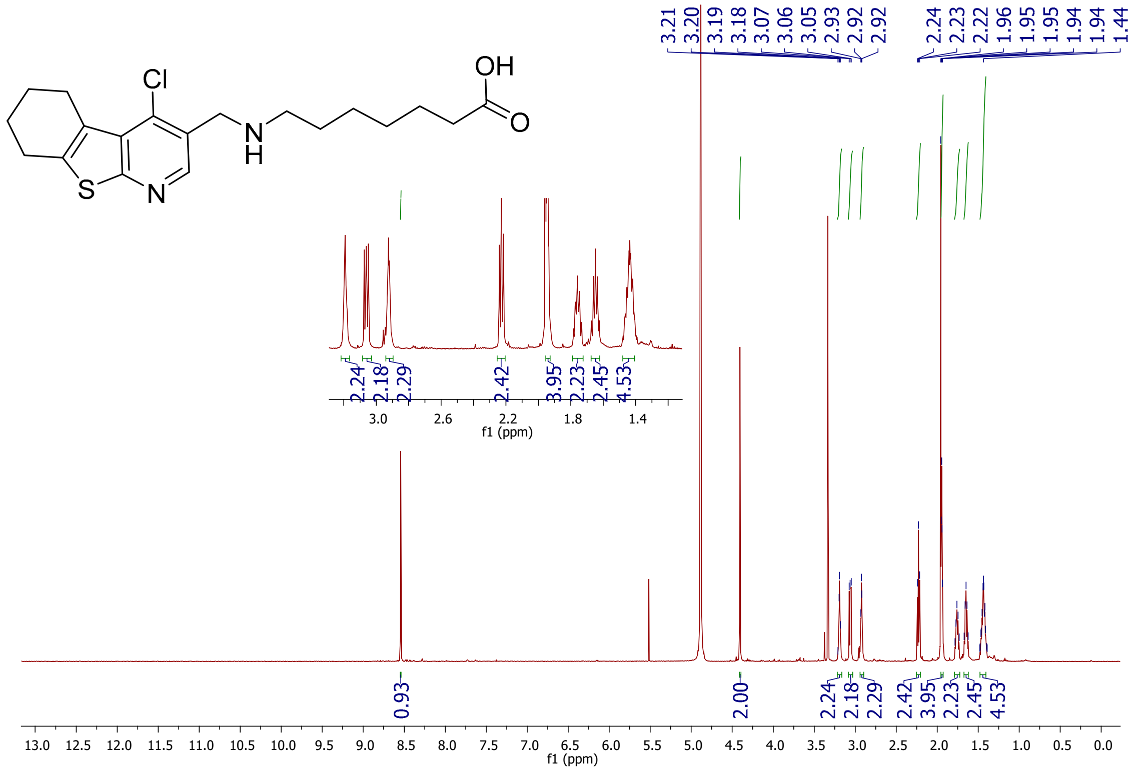

Figure S20. ^{1}H NMR of compound **5b** (600 MHz, MeOD).

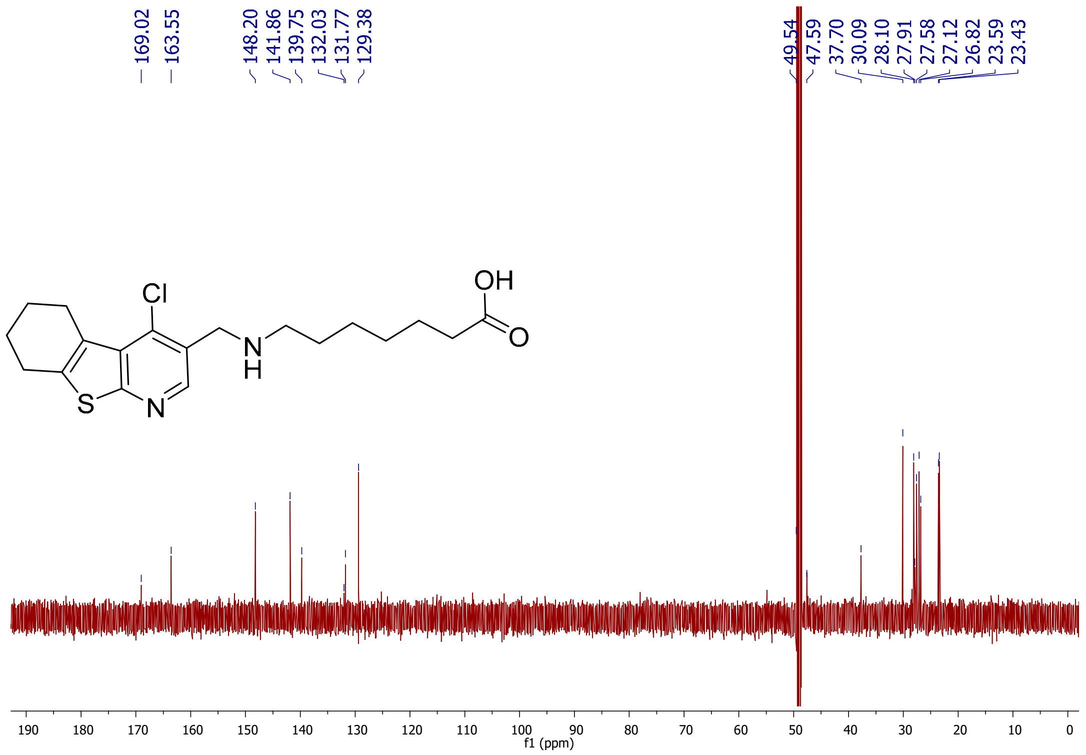

Figure S21. ^{13}C NMR spectrum of compound **5b** (151 MHz, MeOD).

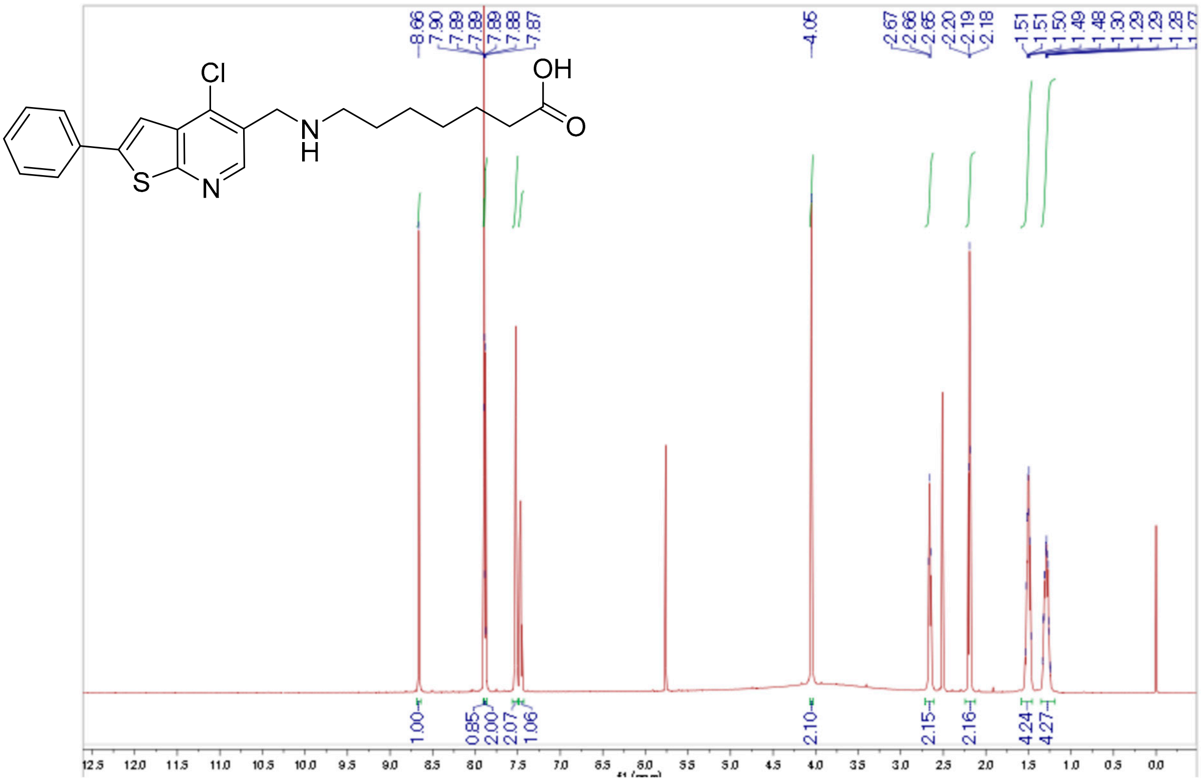

Figure S22. ^{1}H NMR of compound **5c** (600 MHz, DMSO-d_6).

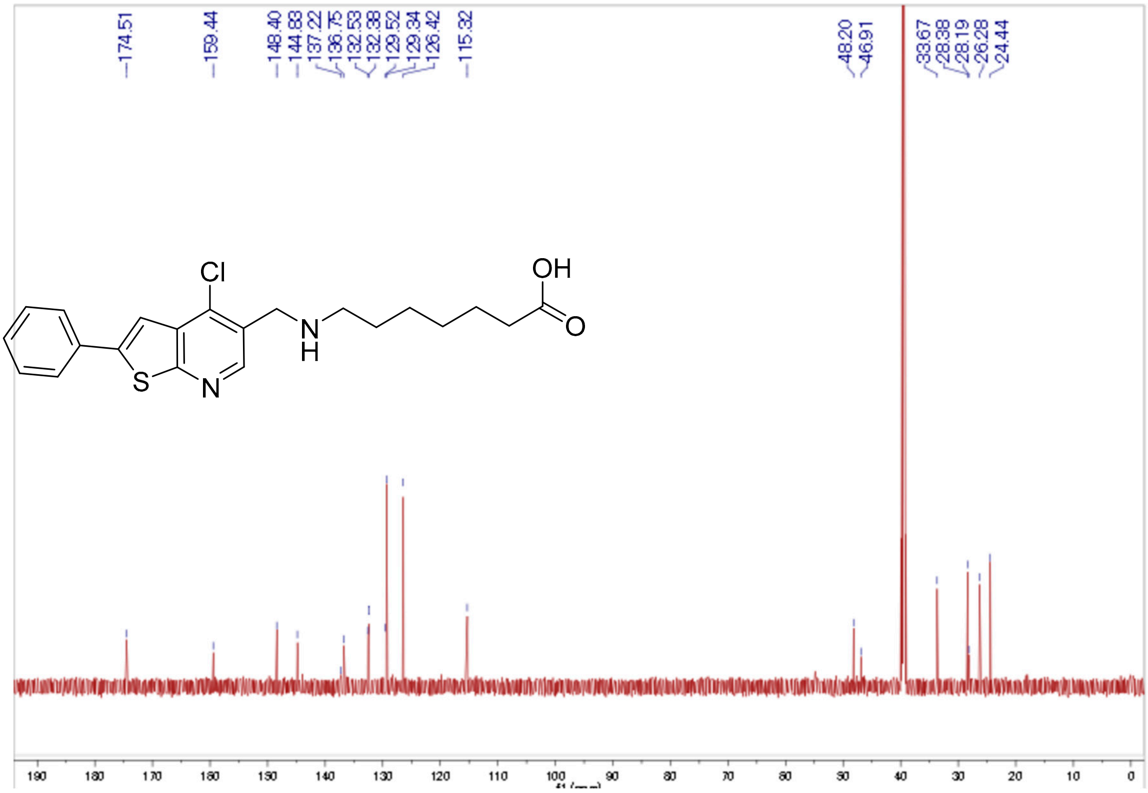

Figure S23. ^{13}C NMR spectrum of compound **5c** (151 MHz, DMSO-d_6).

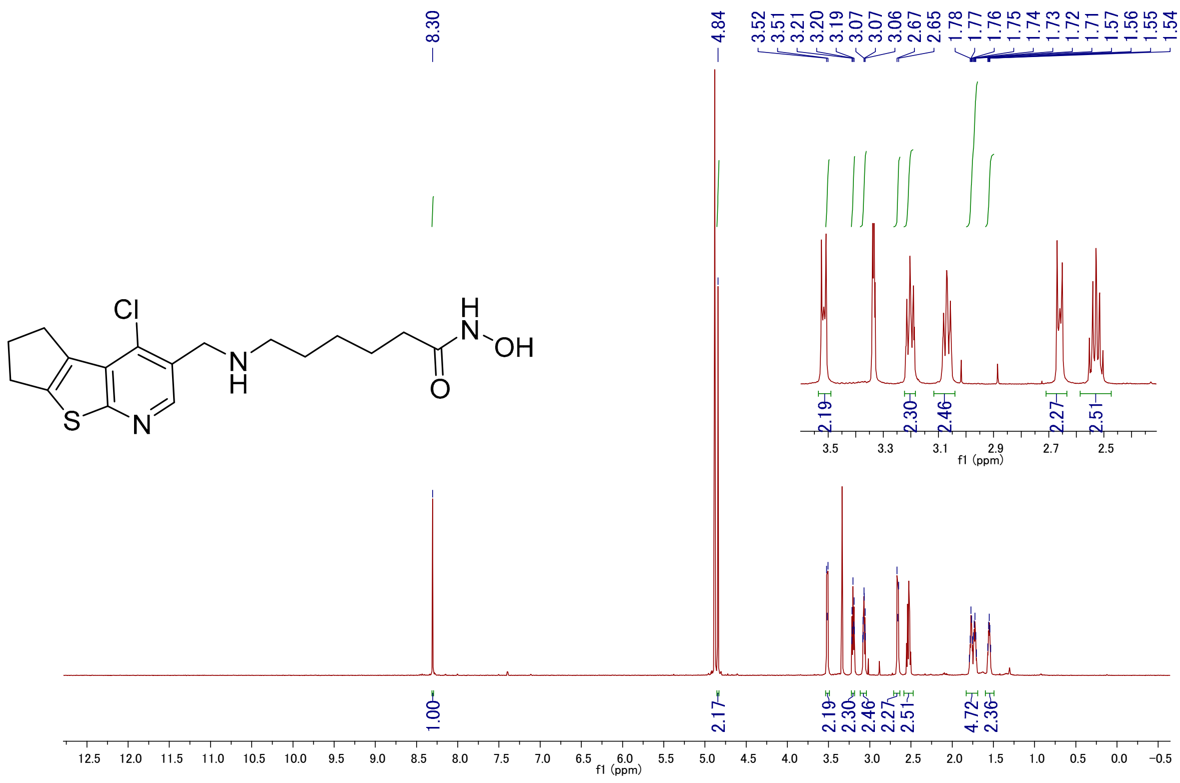

Figure S24. ^{1}H NMR of compound **6a** (600 MHz, MeOD).

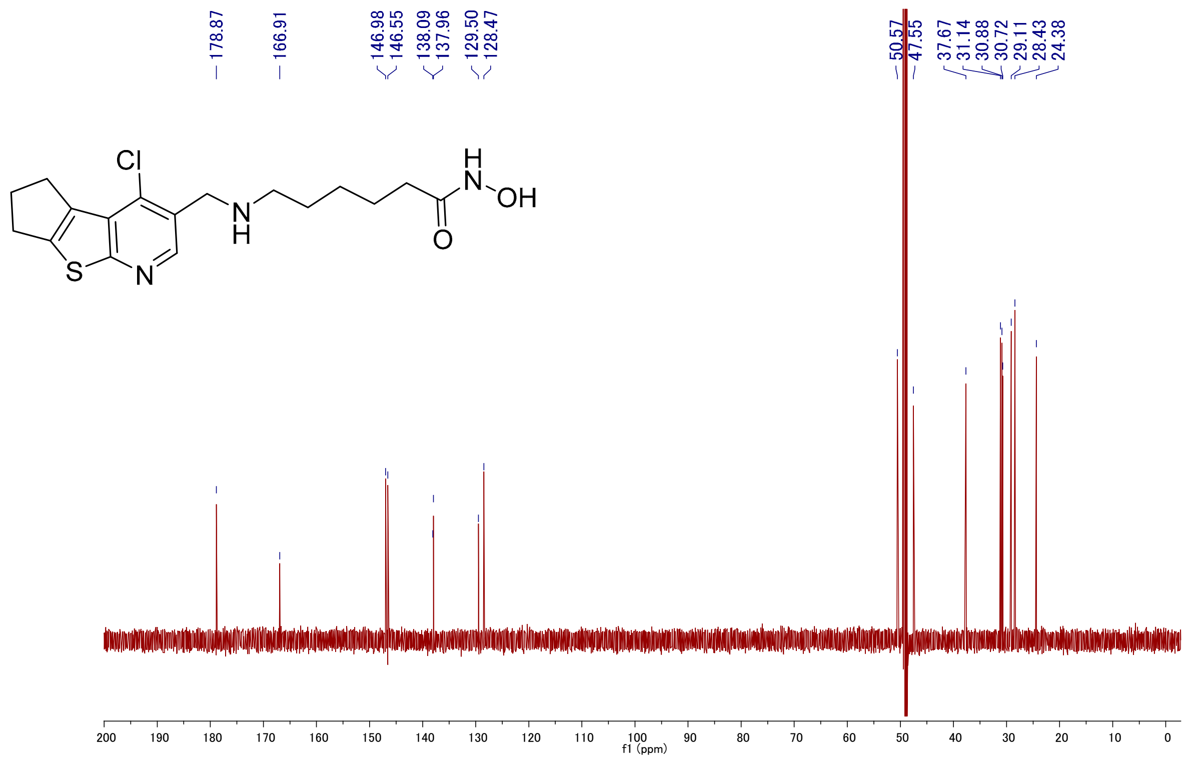

Figure S25. ^{13}C NMR spectrum of compound **6a** (151 MHz, MeOD).

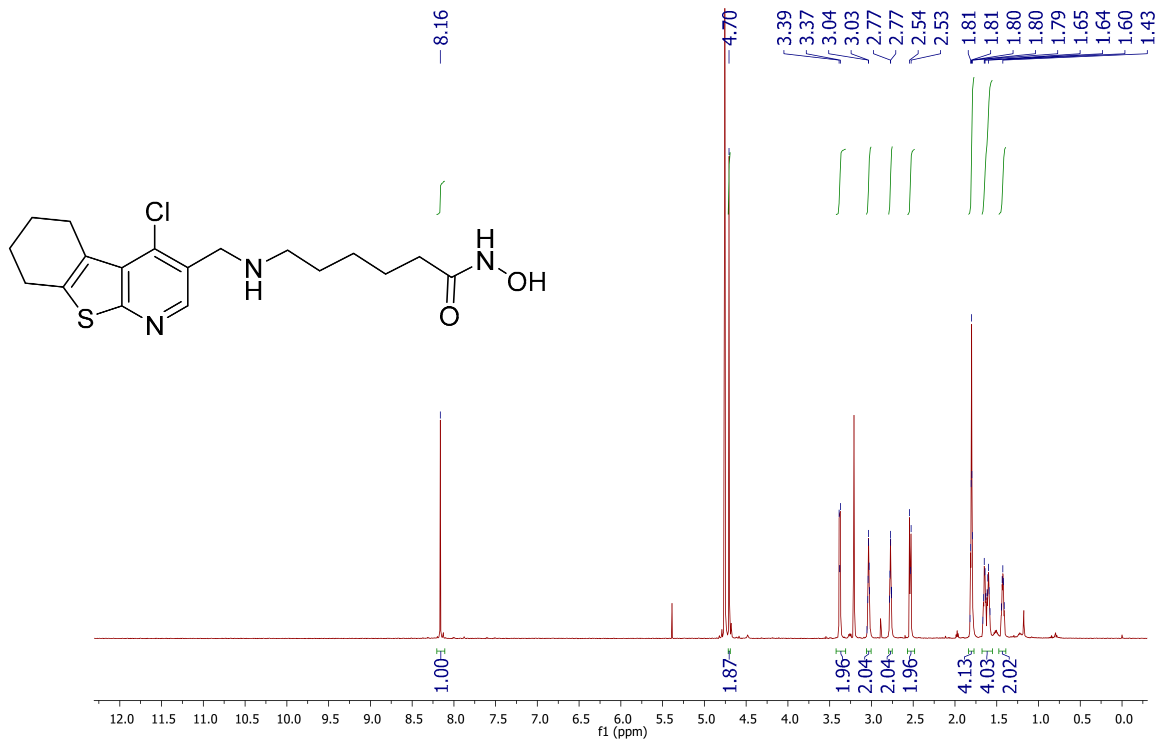

Figure S26. ^{1}H NMR of compound **6b** (600 MHz, MeOD).

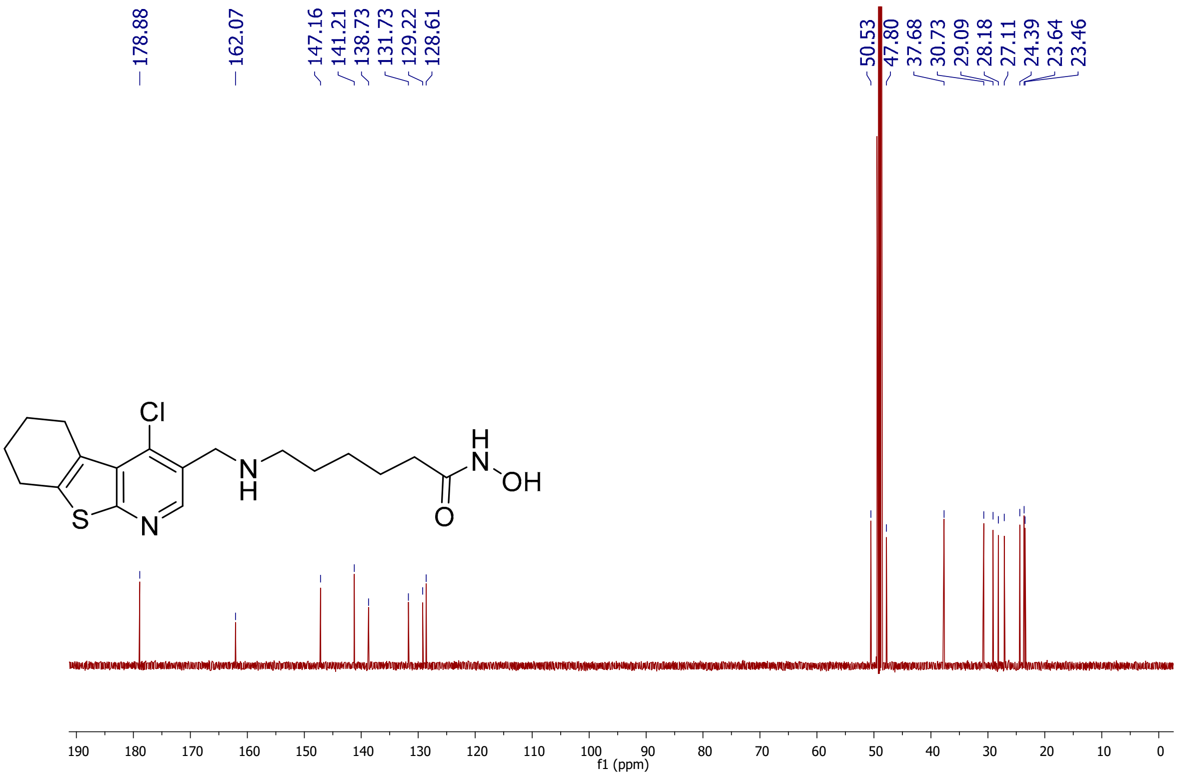

Figure S27. ^{13}C NMR spectrum of compound **6b** (151 MHz, MeOD).

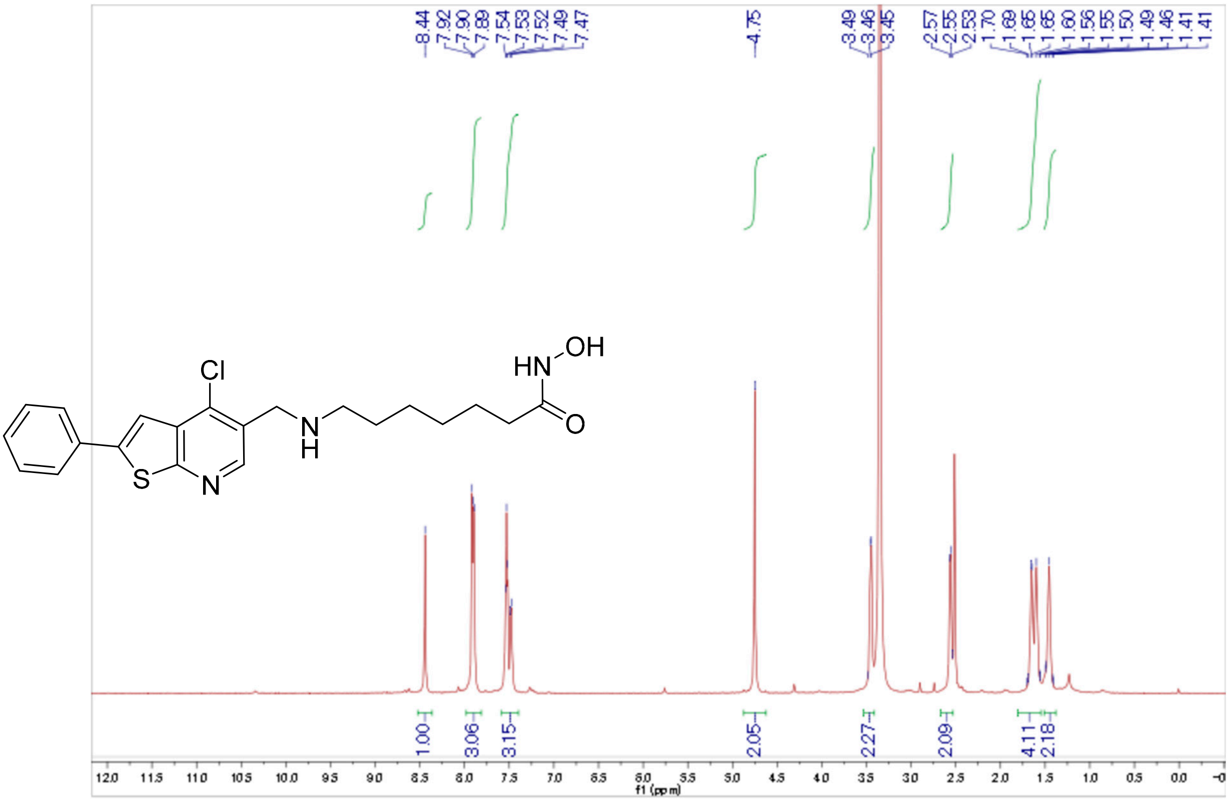

Figure S28. ^{1}H NMR of compound **6c** (600 MHz, DMSO-*d*6).

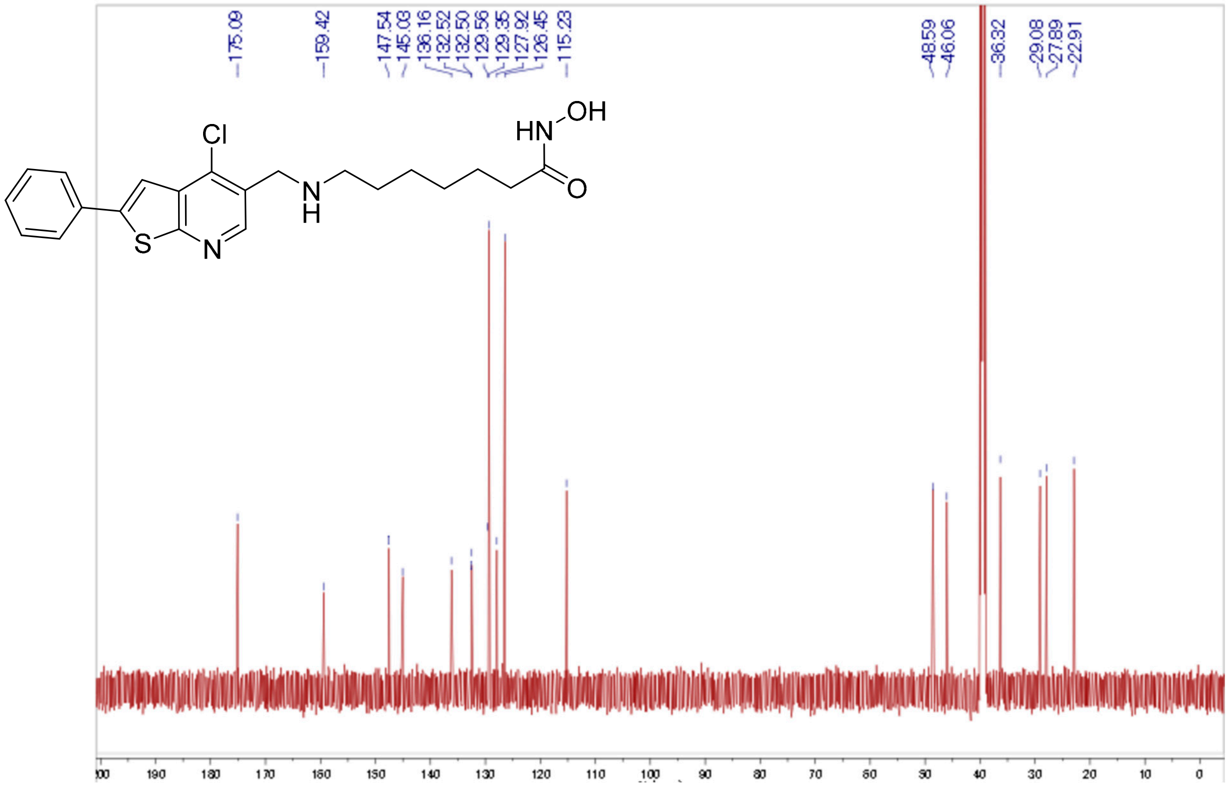

Figure S29. ^{13}C NMR spectrum of compound **6c** (151 MHz, DMSO-*d*6).

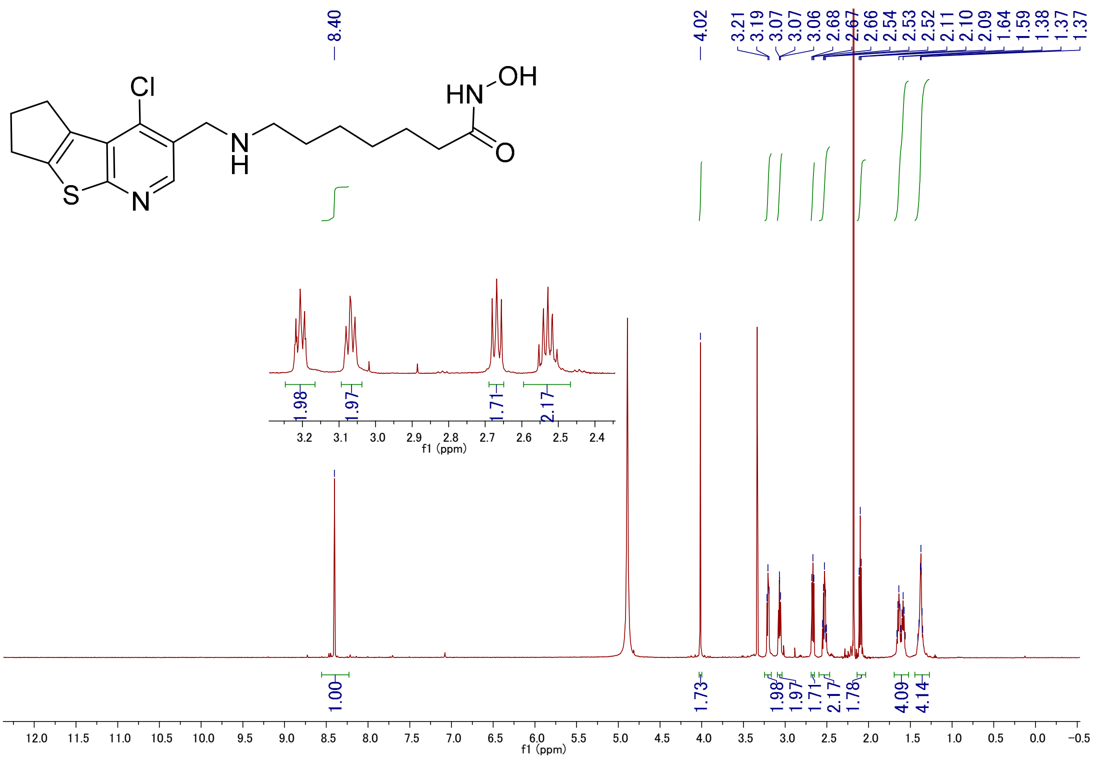

Figure S30. ^{1}H NMR of compound **7a** (600 MHz, MeOD).

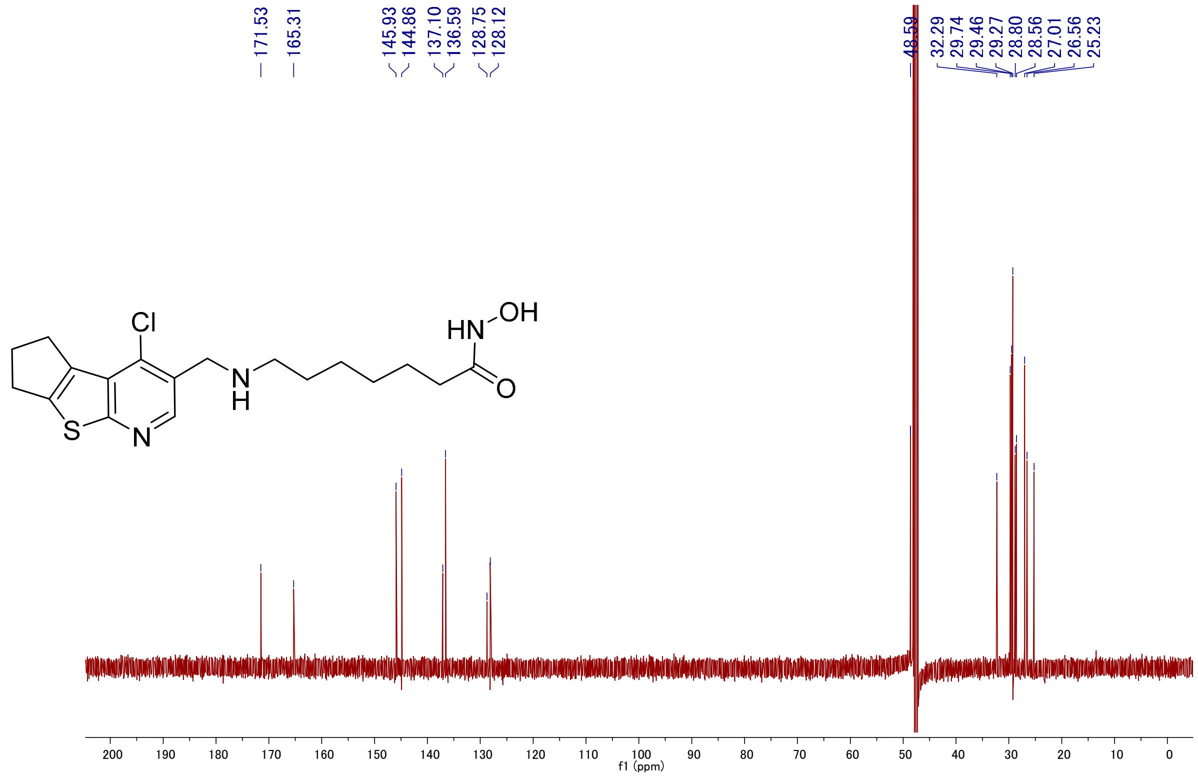

Figure S31. ^{13}C NMR spectrum of compound **7a** (151 MHz, MeOD).

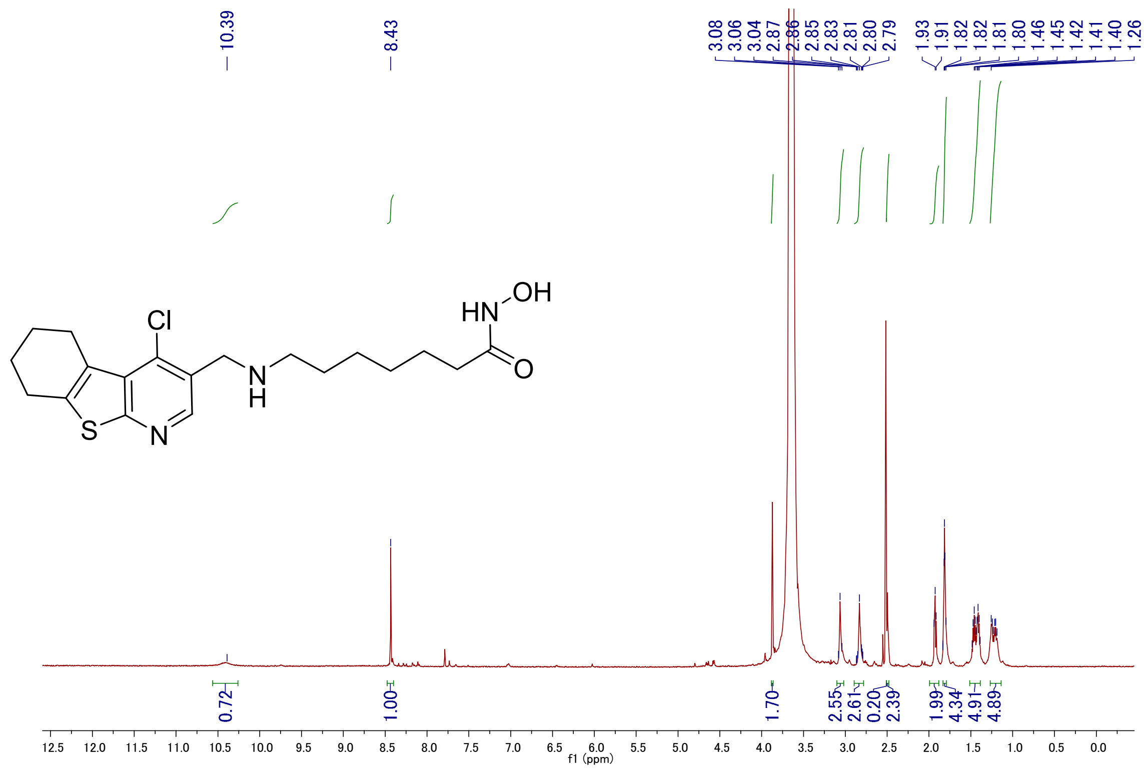

Figure S32. 1H NMR of compound **7b** (600 MHz, DMSO-*d*6).

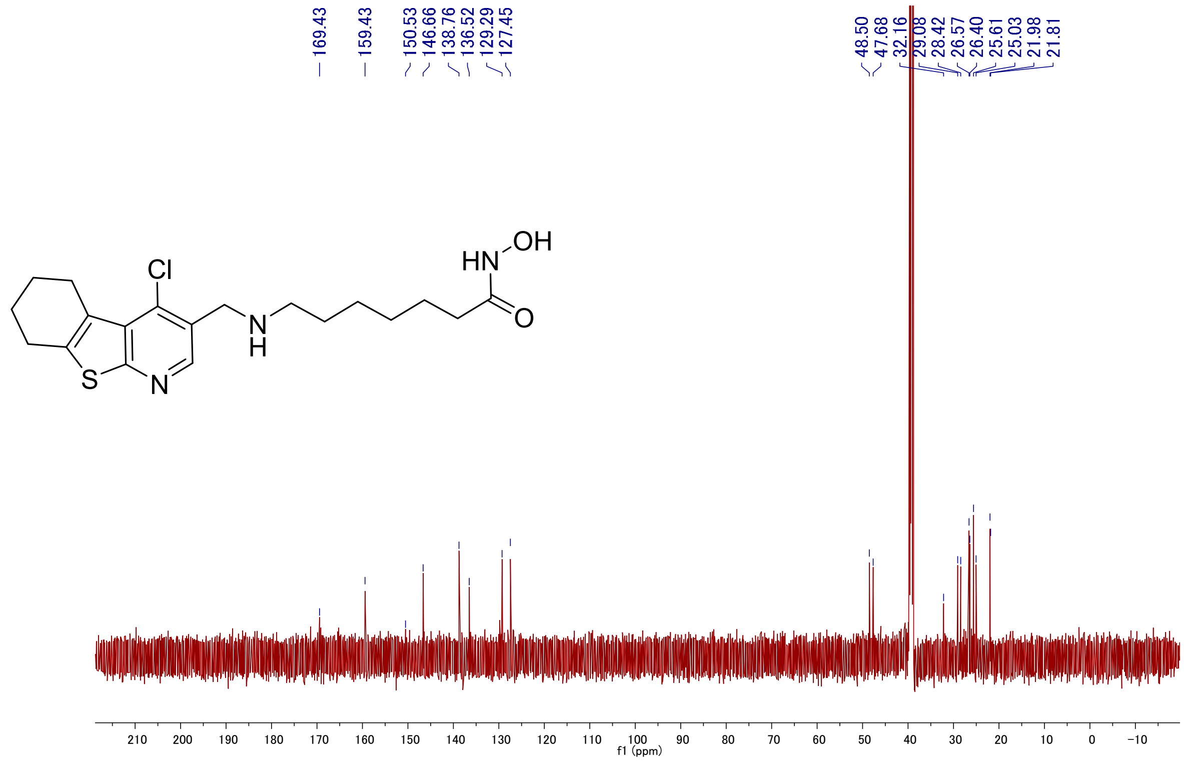

Figure S33. ^{13}C NMR spectrum of compound **7b** (151 MHz, DMSO-*d*6).

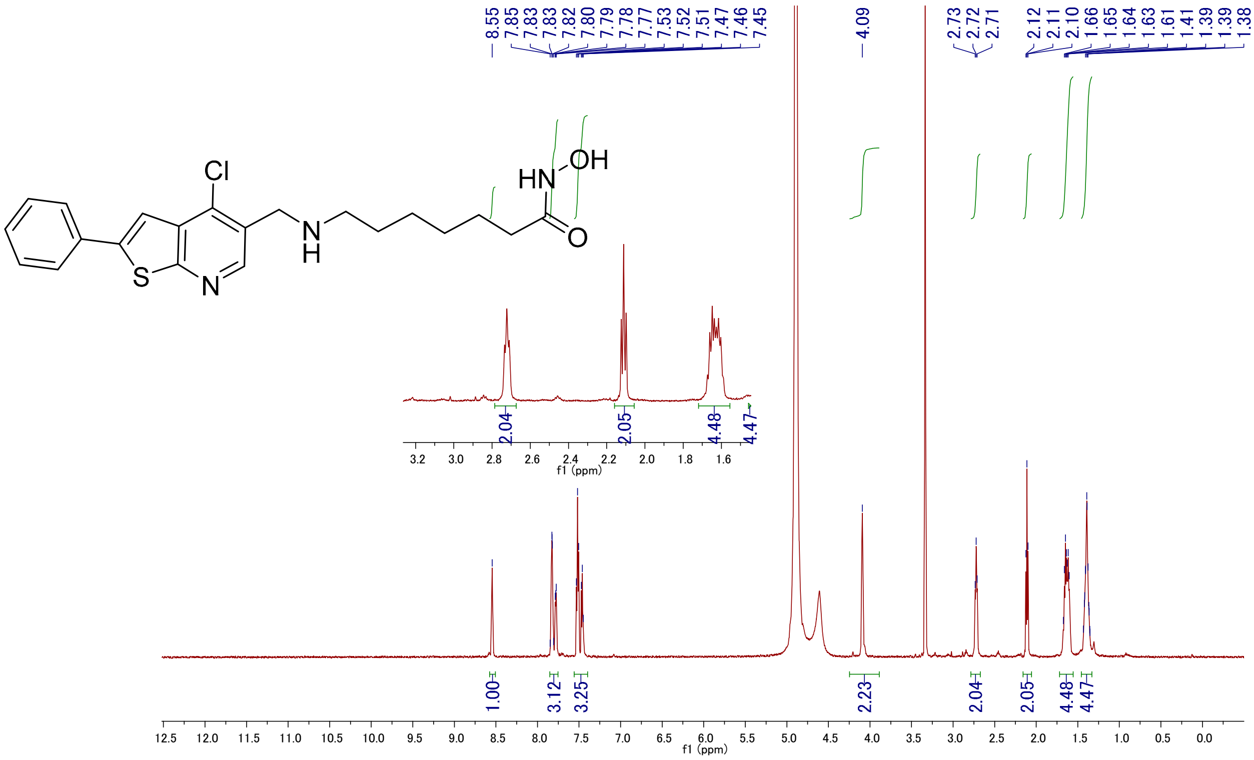

Figure S34. 1H NMR of compound **7c** (600 MHz, MeOD).

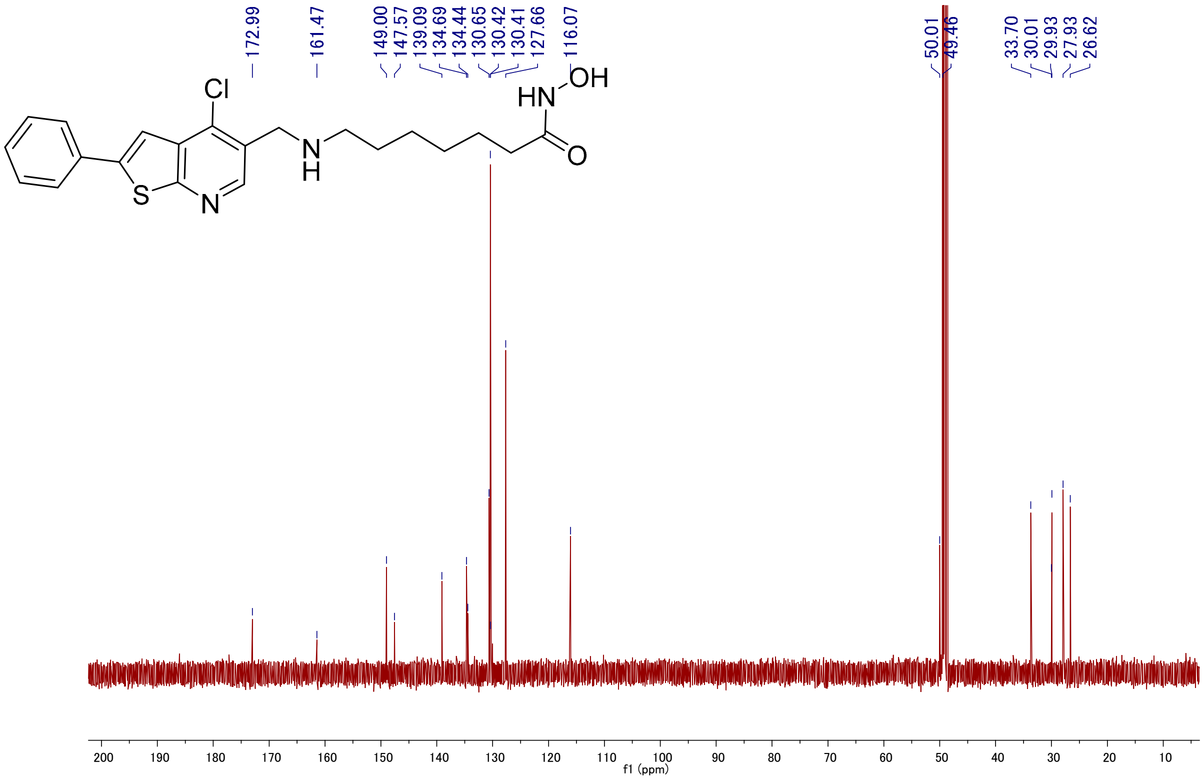

Figure S35. ^{13}C NMR spectrum of compound **7c** (151 MHz, MeOD).

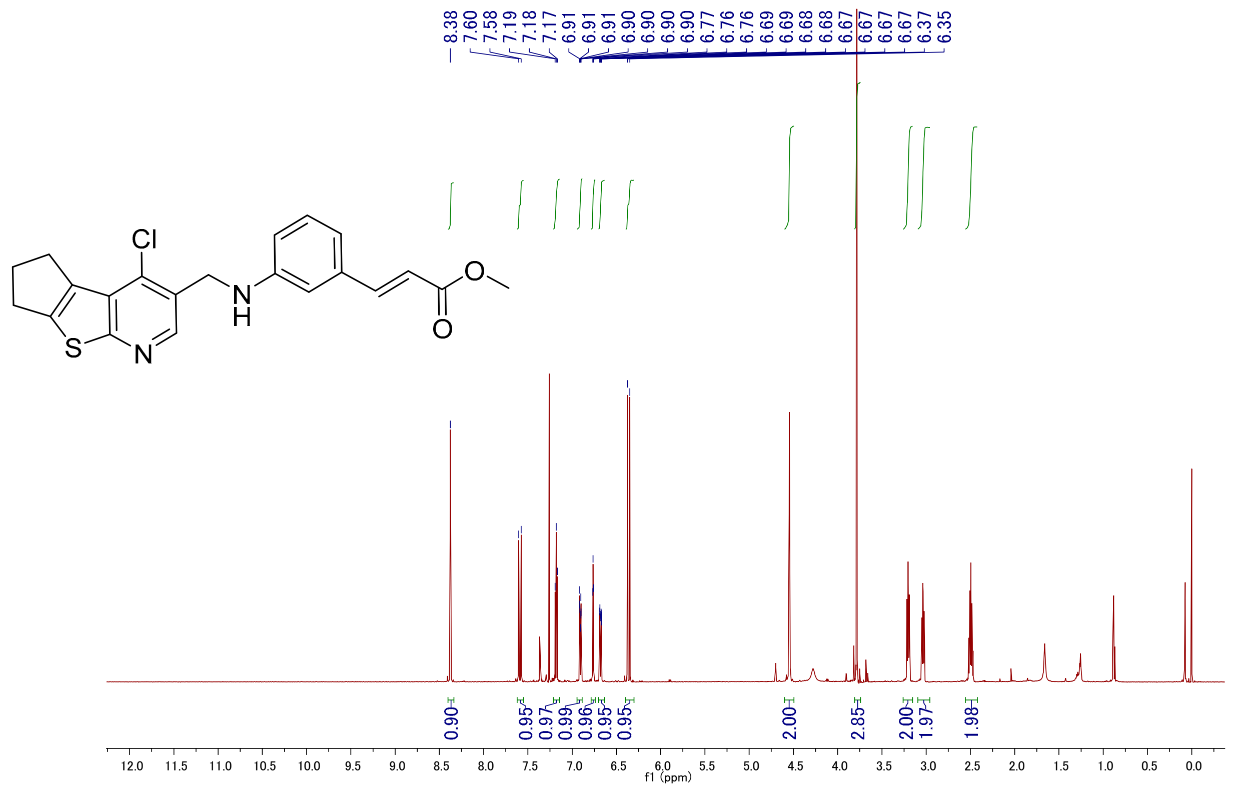

Figure S36. 1H NMR of compound **8a** (600 MHz, $CDCl_3$).

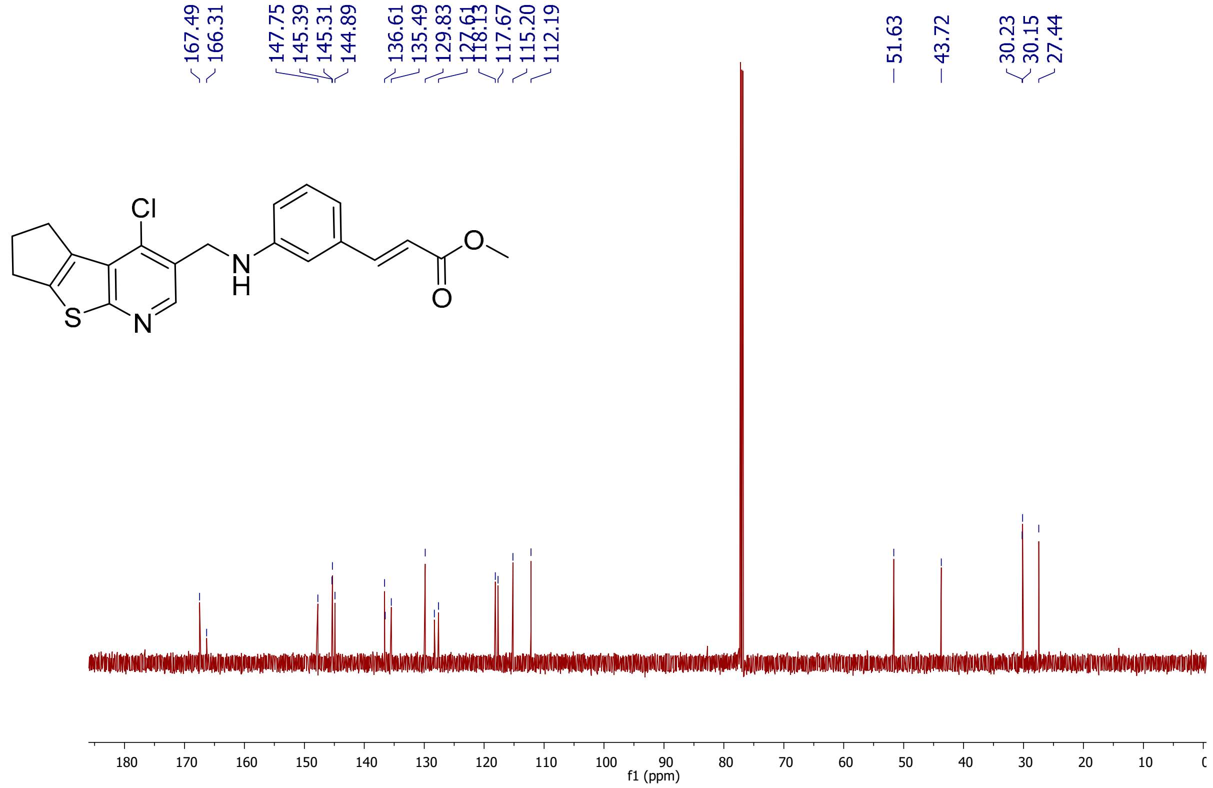

Figure S37. ^{13}C NMR spectrum of compound **8a** (151 MHz, $CDCl_3$).

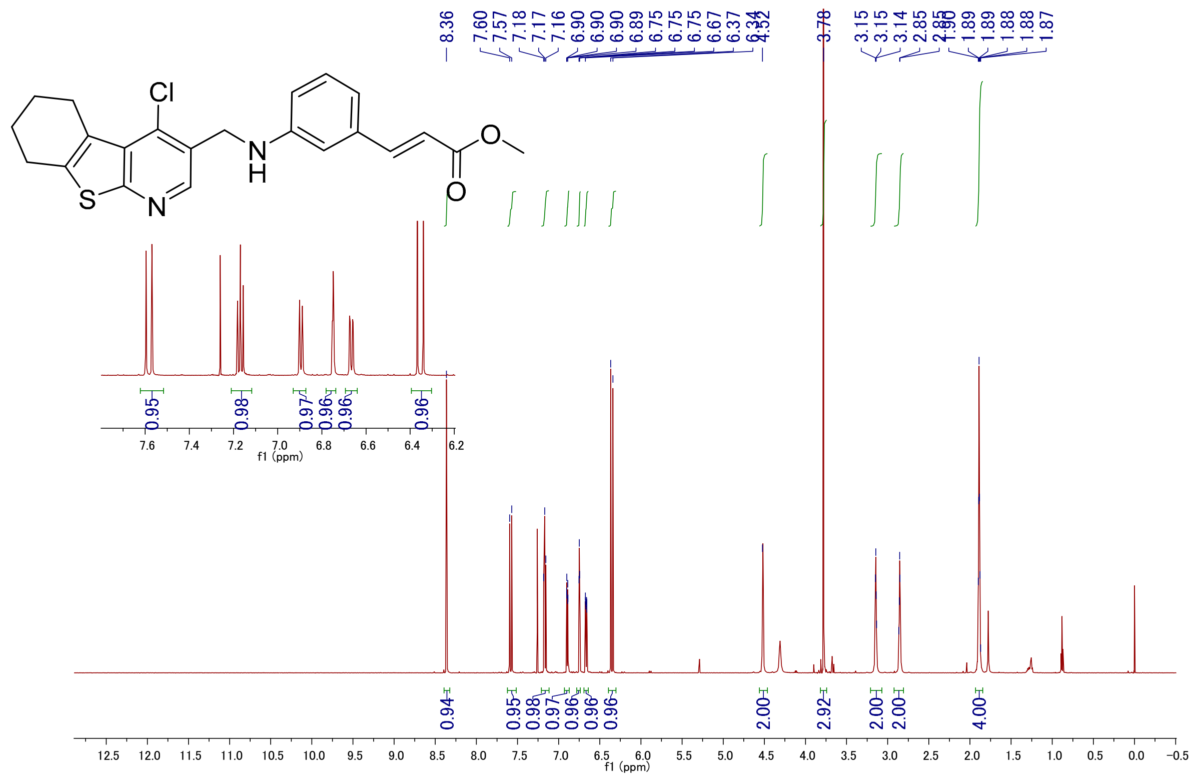

Figure S38. 1H NMR of compound **8b** (600 MHz, $CDCl_3$).

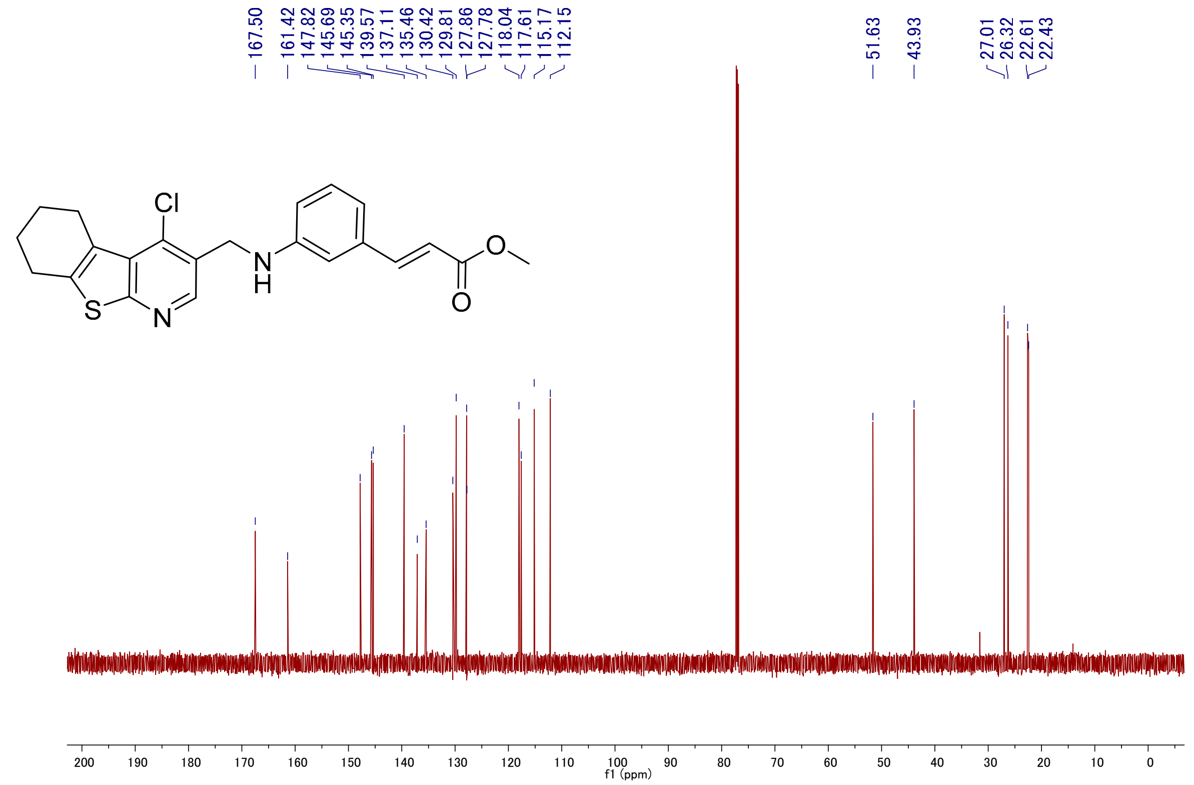

Figure S39. ^{13}C NMR spectrum of compound **8b** (151 MHz, $CDCl_3$).

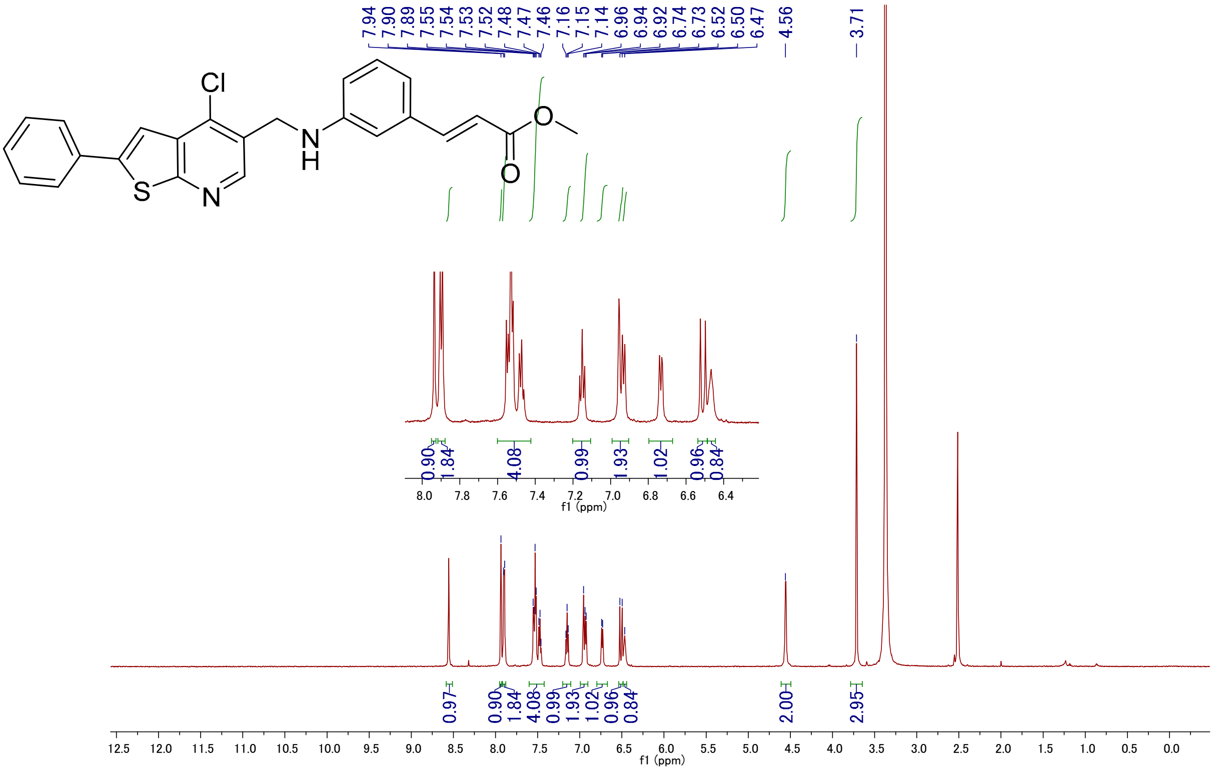

Figure S40. 1H NMR of compound **8c** (600 MHz, DMSO-d_6).

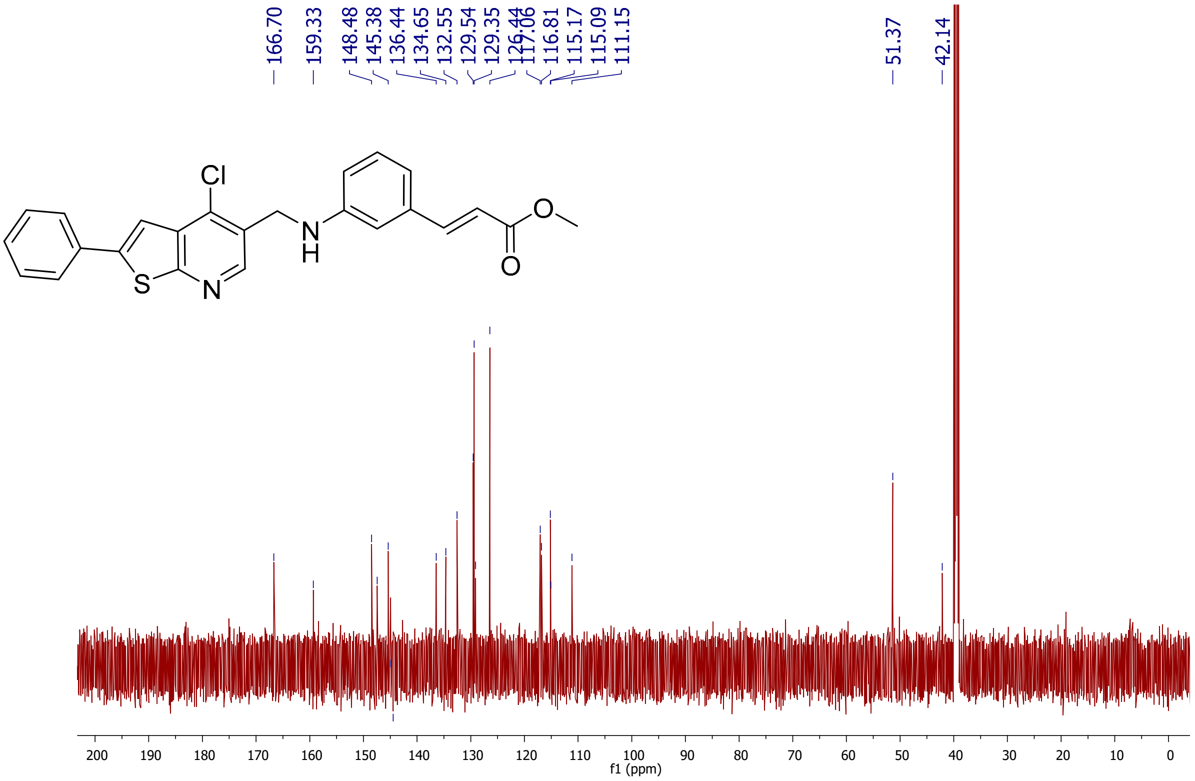

Figure S41. ^{13}C NMR spectrum of compound **8c** (151 MHz, DMSO-d_6).

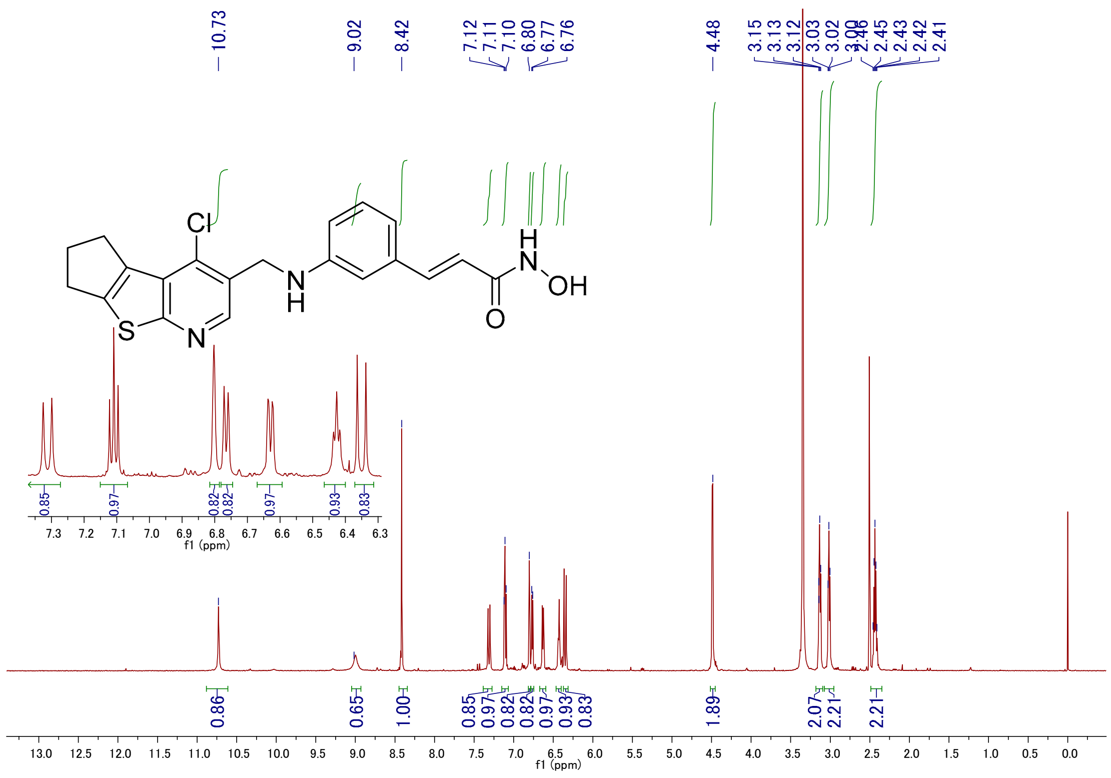

Figure S42. ^{1}H NMR of compound **9a** (600 MHz, DMSO-d_6).

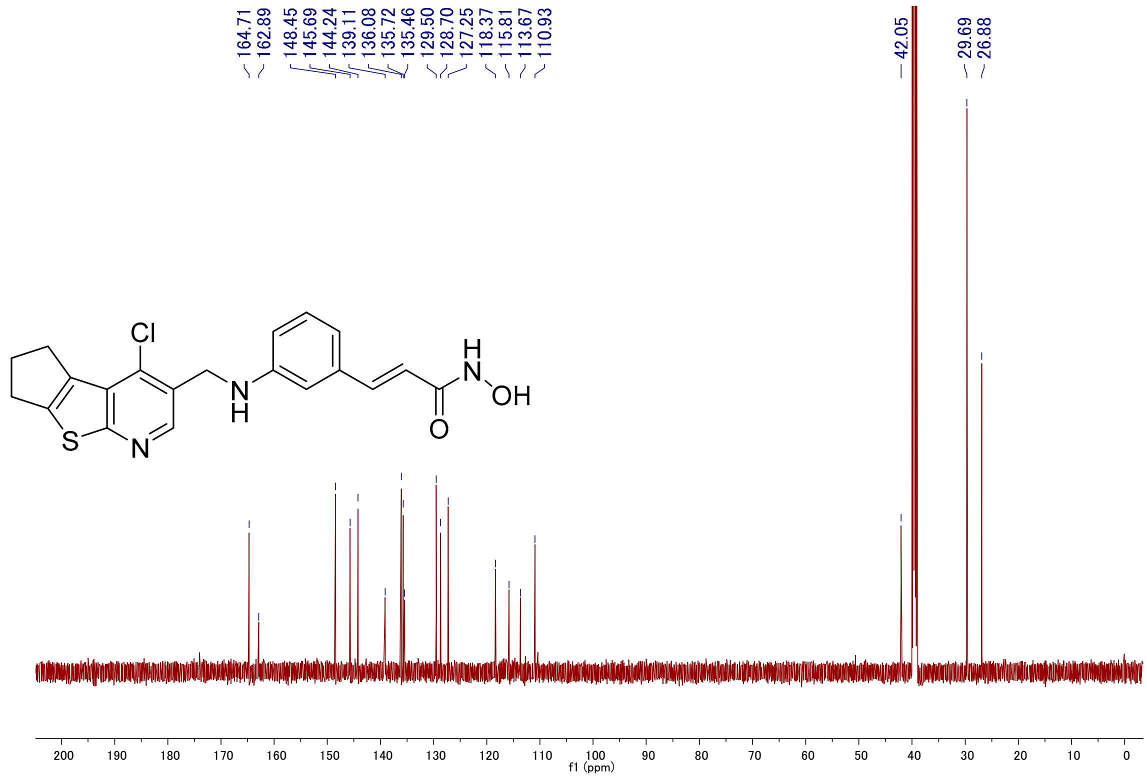

Figure S43. ^{13}C NMR spectrum of compound **9a** (151 MHz, DMSO-d_6).

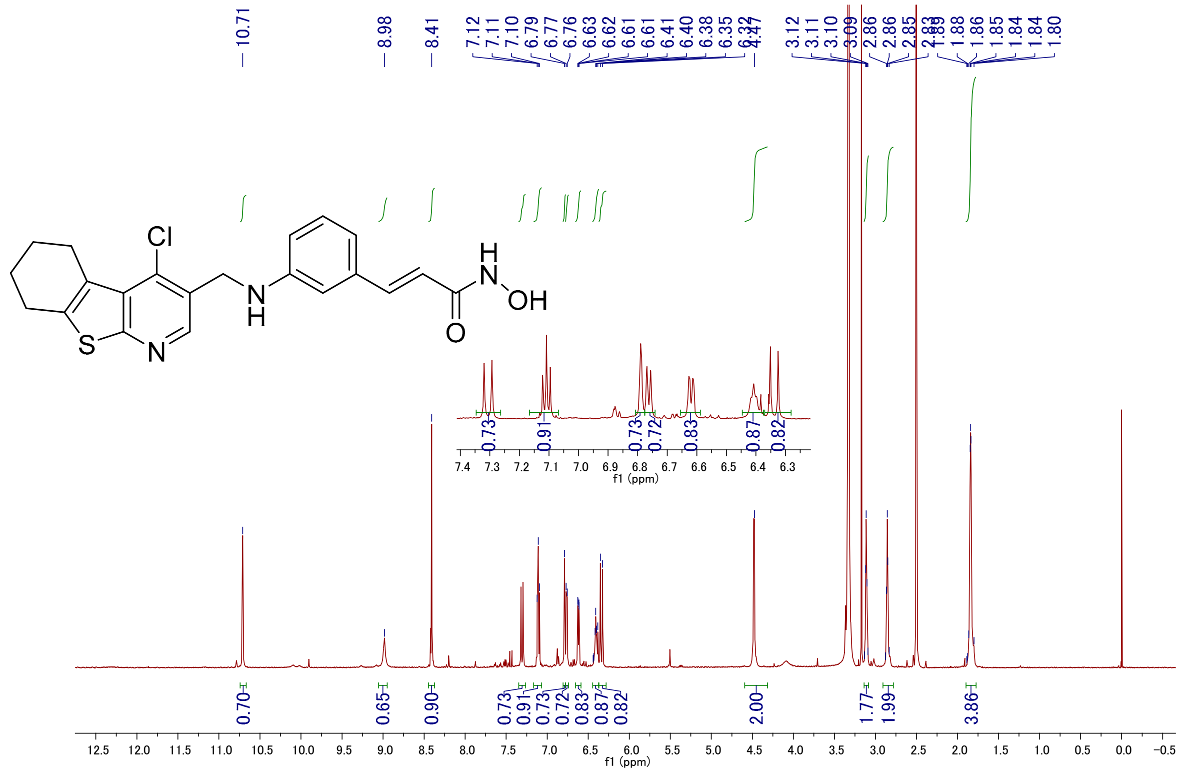

Figure S44. ^{1}H NMR of compound **9b** (600 MHz, DMSO-d_6).

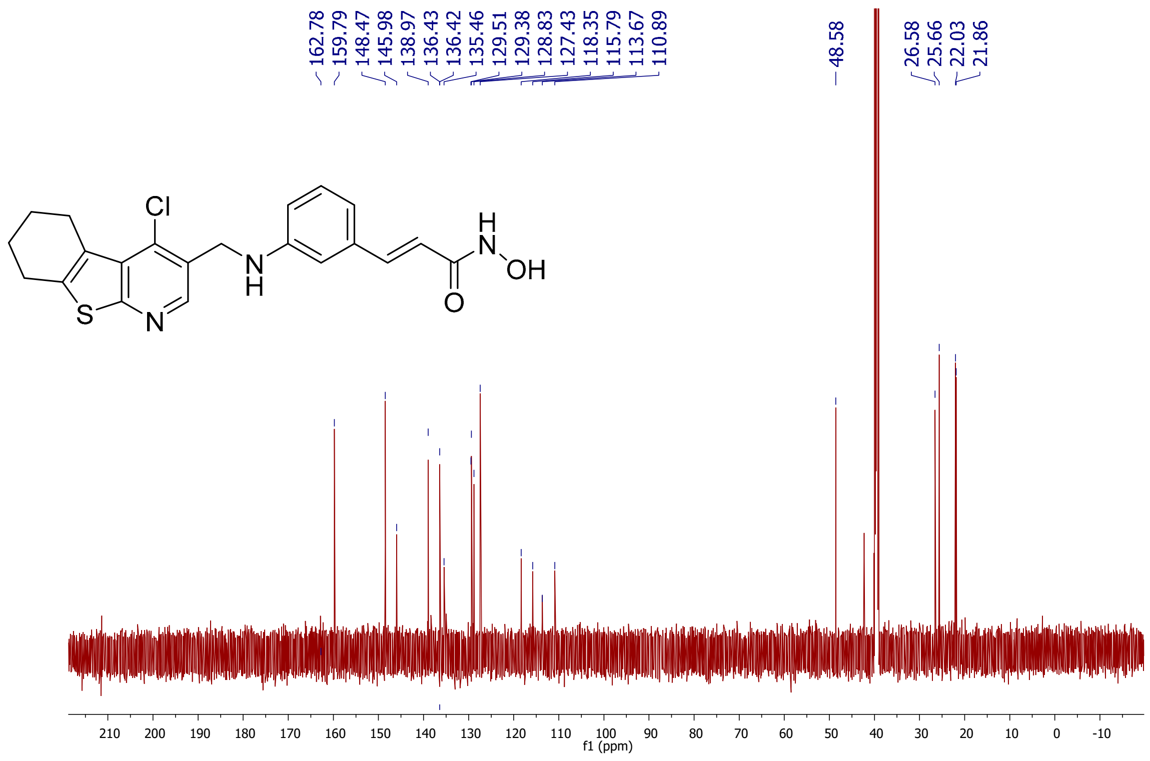

Figure S45. ^{13}C NMR spectrum of compound **9b** (151 MHz, DMSO-d_6).

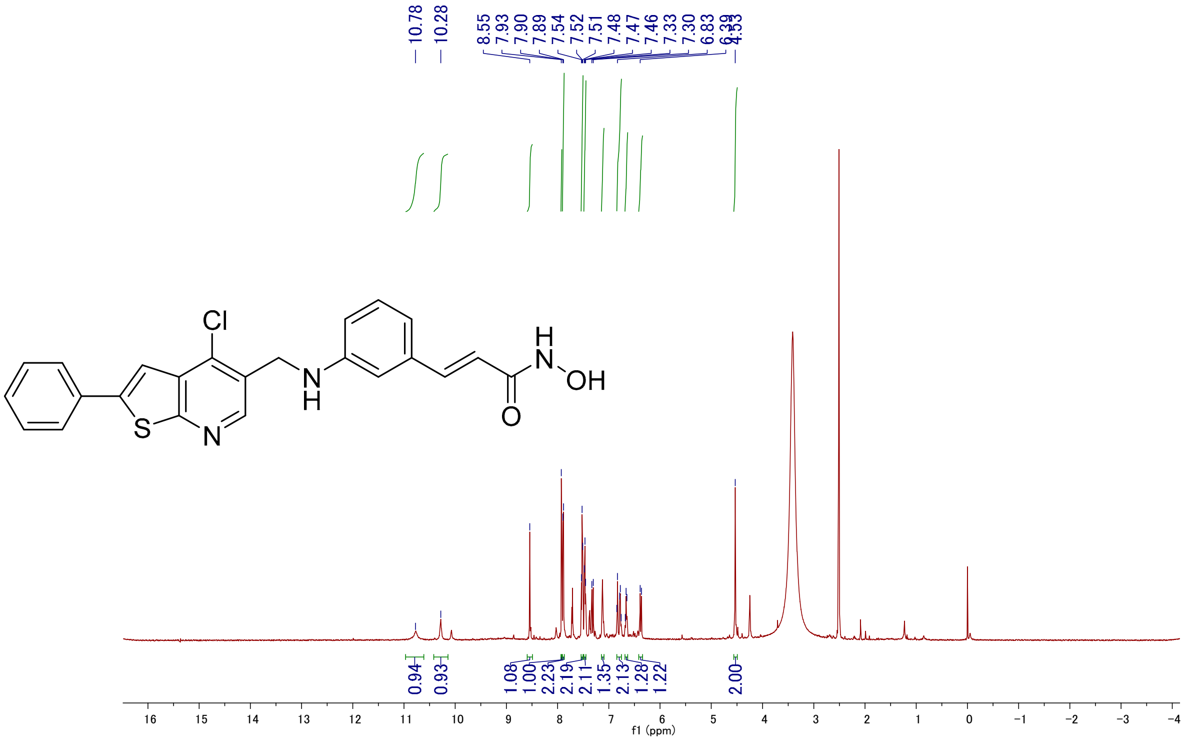

Figure S46. ^{1}H NMR of compound **9c** (600 MHz, , DMSO-d_6).

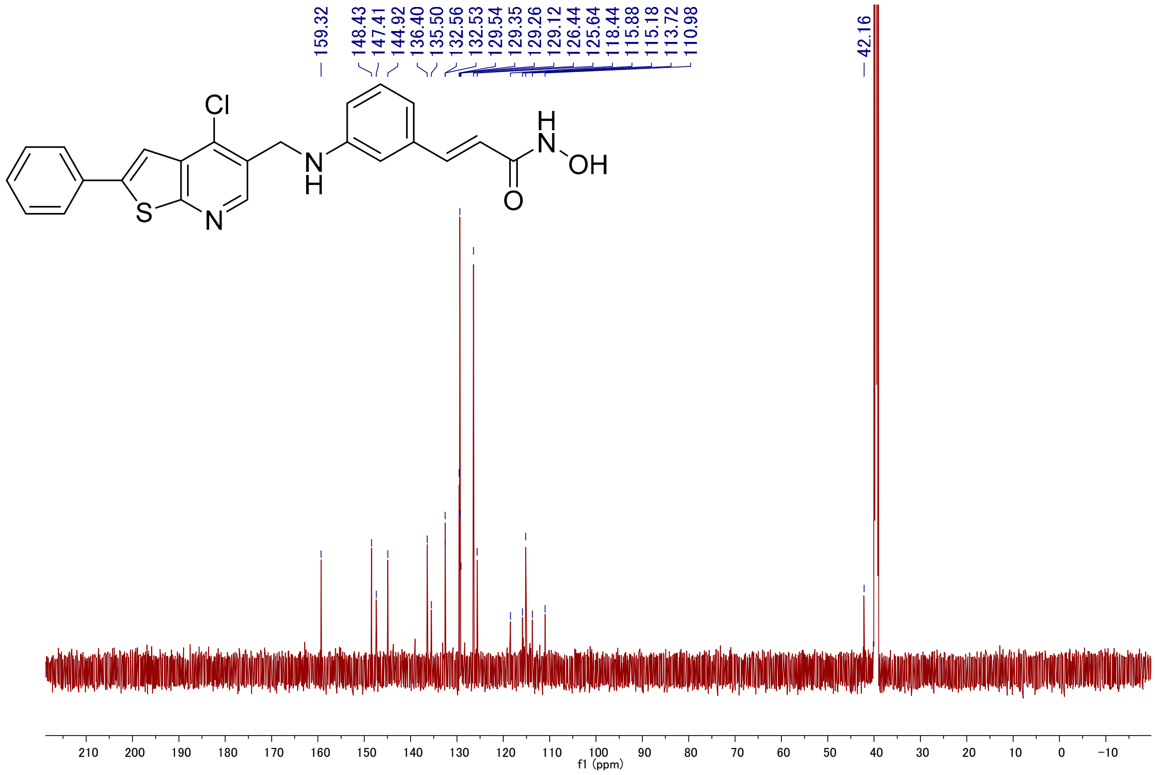

Figure S47. ^{13}C NMR spectrum of compound **9c** (151 MHz, DMSO-d_6).

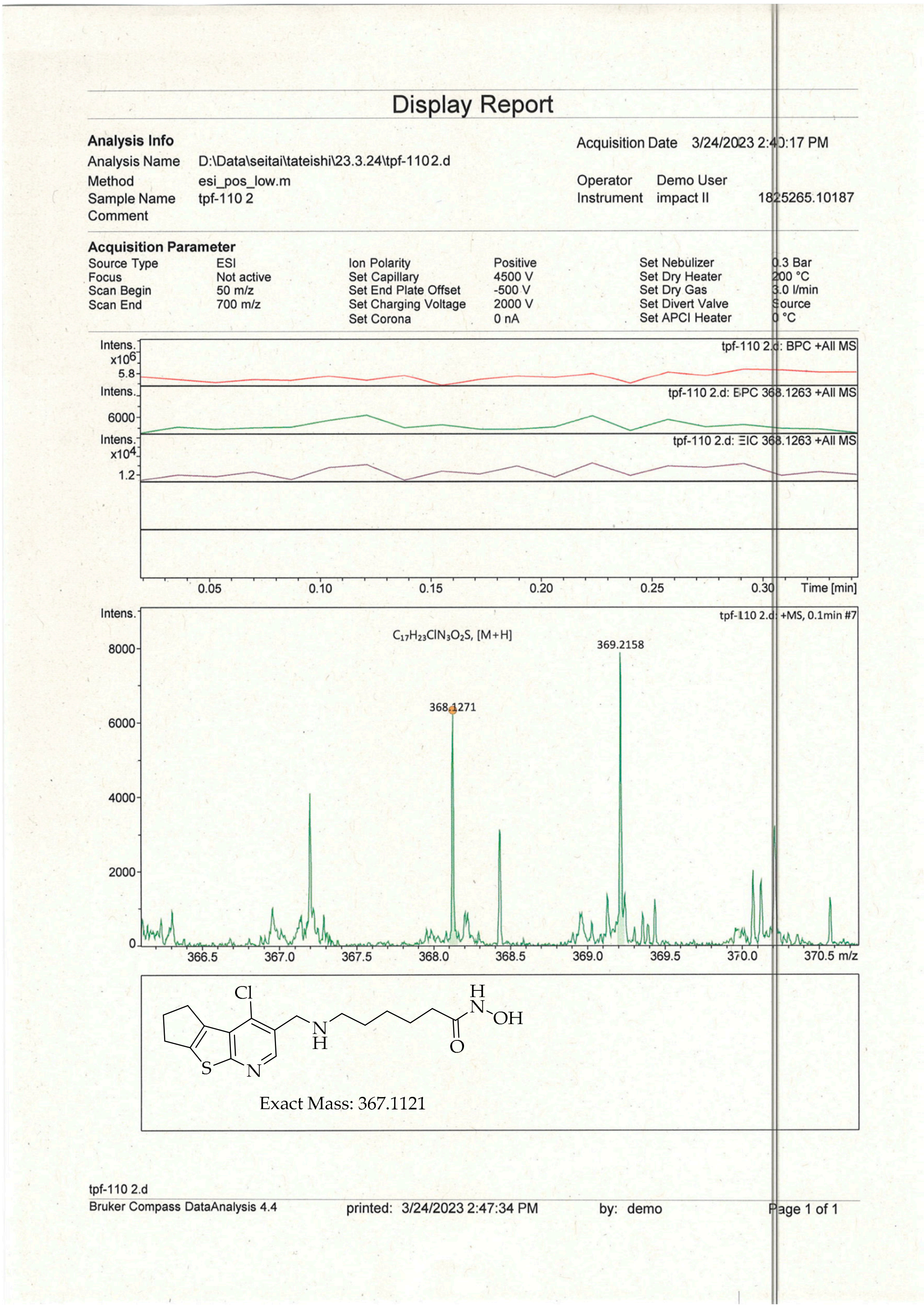
Display Report

Analysis Info

Analysis Name	D:\Data\seitai\tateishi\23.3.24\tpf-110 2.d	Acquisition Date	3/24/2023 2:40:17 PM
Method	esi_pos_low.m	Operator	Demo User
Sample Name	tpf-110 2	Instrument	impact II 1825265.10187
Comment			

Acquisition Parameter

Source Type	ESI	Ion Polarity	Positive	Set Nebulizer	0.3 Bar
Focus	Not active	Set Capillary	4500 V	Set Dry Heater	200 °C
Scan Begin	50 m/z	Set End Plate Offset	-500 V	Set Dry Gas	3.0 l/min
Scan End	700 m/z	Set Charging Voltage	2000 V	Set Divert Valve	Source
		Set Corona	0 nA	Set APCI Heater	0 °C

tpf-110 2.d
Bruker Compass DataAnalysis 4.4 printed: 3/24/2023 2:47:34 PM by: demo Page 1 of 1

Figure S48. ESI-HRMs of compound **6a**.

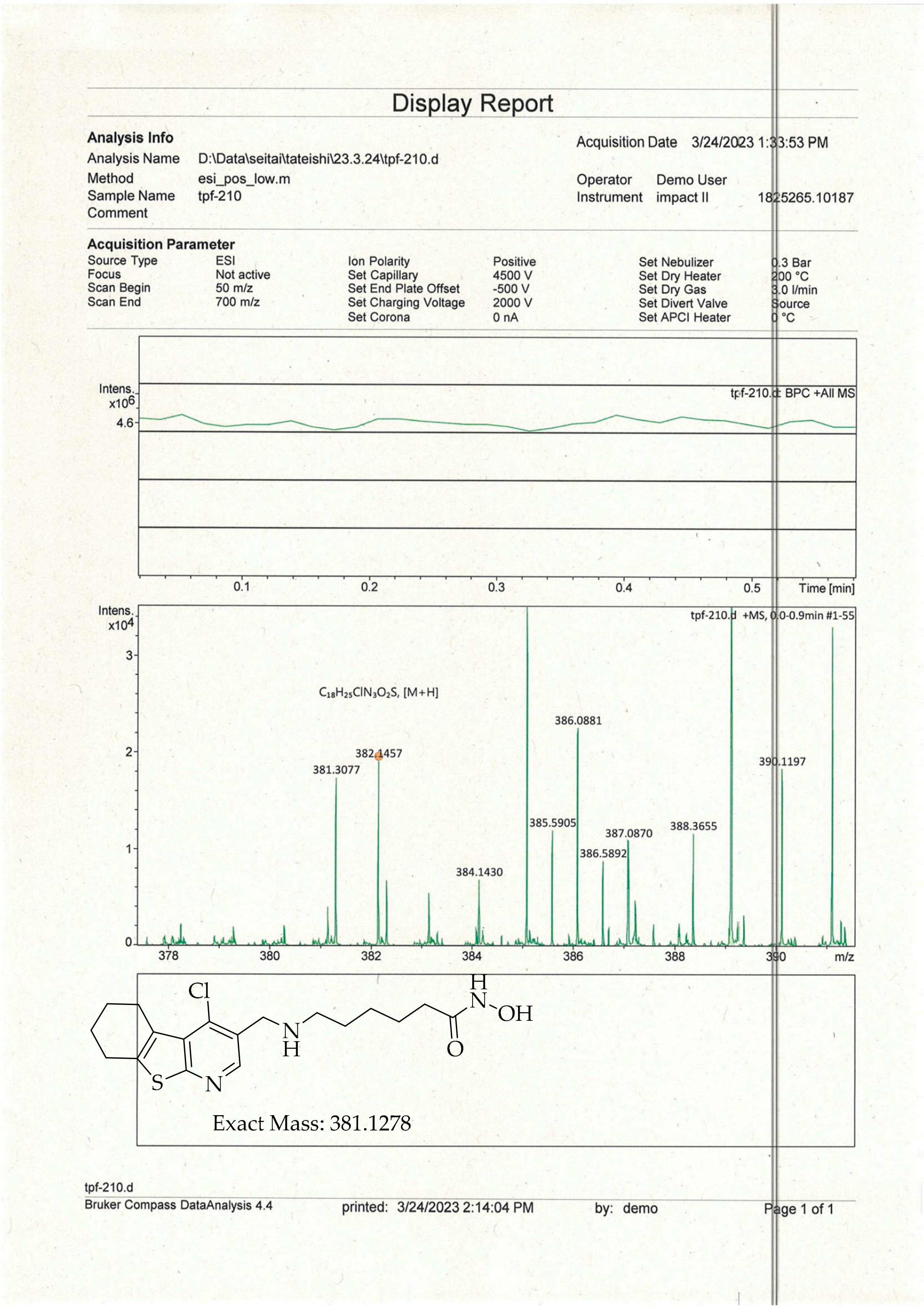

Figure S49. ESI-HRMs of compound **6b**.

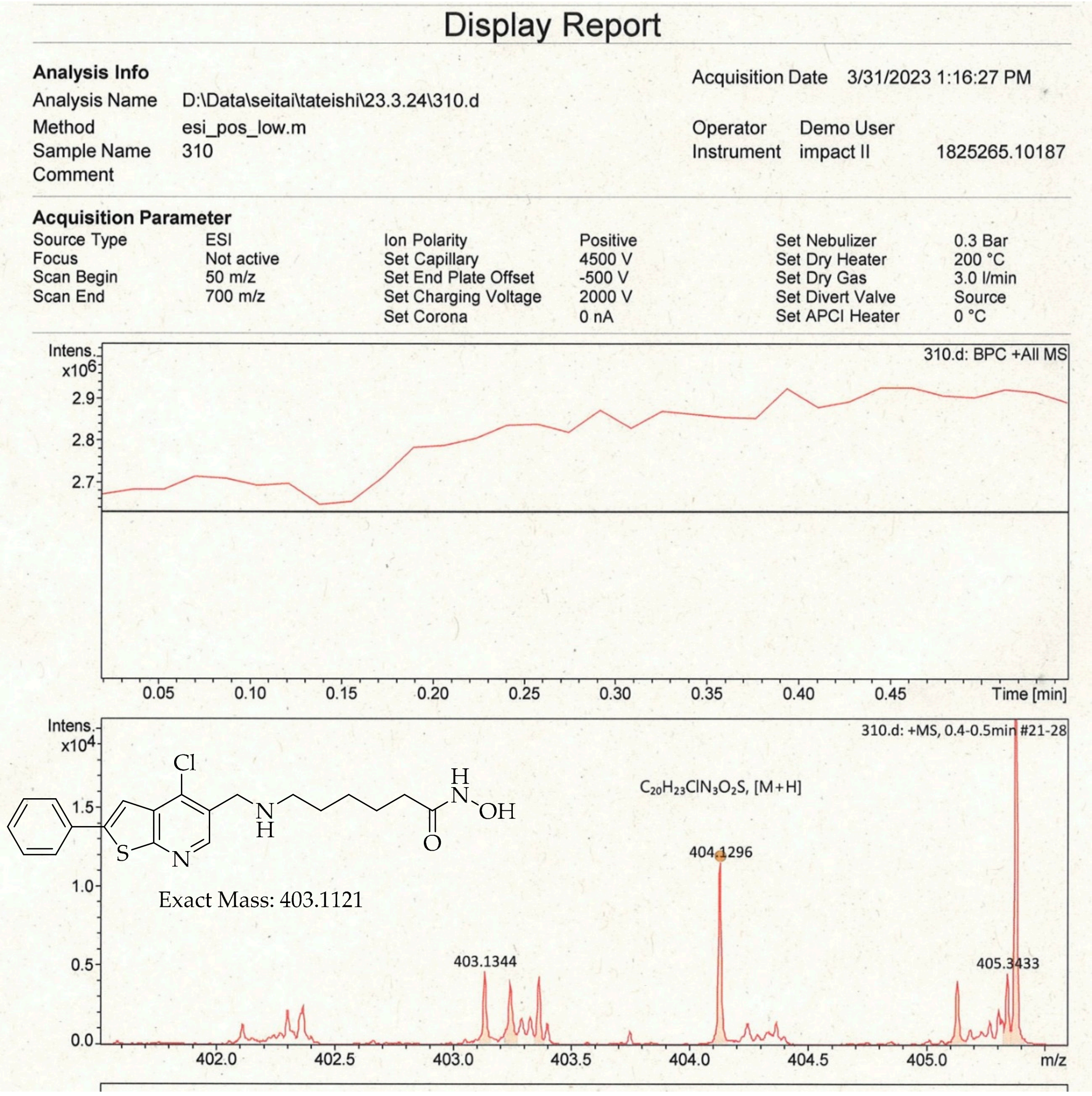

Figure S50. ESI-HRMs of compound **6c**.

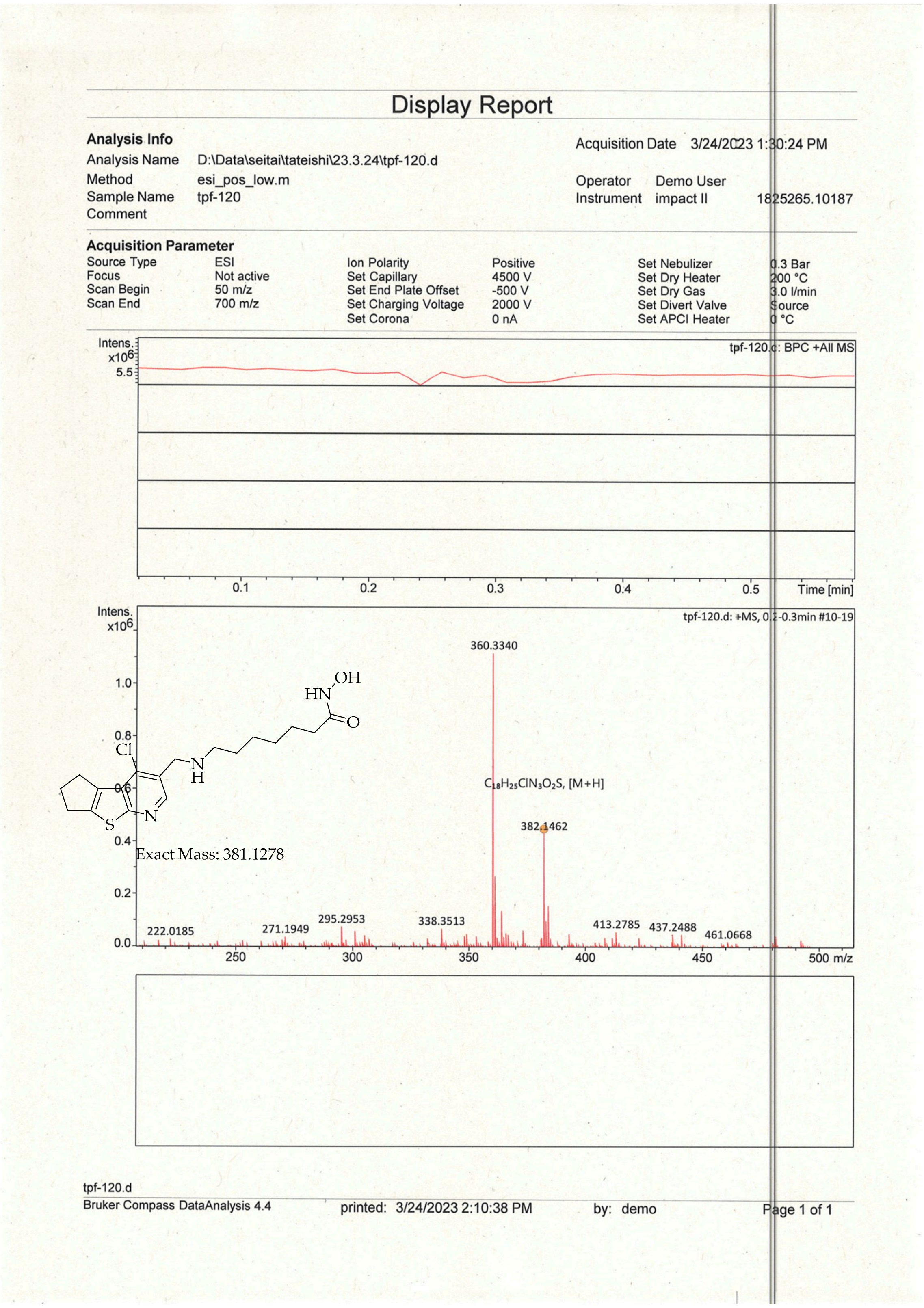

Display Report

Analysis Info

Analysis Name	D:\Data\seitai\tateishi\23.3.24\tpf-120.d	Acquisition Date	3/24/2023 1:30:24 PM
Method	esi_pos_low.m	Operator	Demo User
Sample Name	tpf-120	Instrument	impact II 1825265.10187
Comment			

Acquisition Parameter

Source Type	ESI	Ion Polarity	Positive	Set Nebulizer	0.3 Bar
Focus	Not active	Set Capillary	4500 V	Set Dry Heater	200 °C
Scan Begin	50 m/z	Set End Plate Offset	-500 V	Set Dry Gas	3.0 l/min
Scan End	700 m/z	Set Charging Voltage	2000 V	Set Divert Valve	Source
		Set Corona	0 nA	Set APCI Heater	0 °C

tpf-120.d

Bruker Compass DataAnalysis 4.4 printed: 3/24/2023 2:10:38 PM by: demo Page 1 of 1

Figure S51. ESI-HRMs of compound **7a**.

Display Report

Analysis Info

Analysis Name	D:\Data\seitai\tateishi\23.3.24\tpf-220.d	Acquisition Date	3/24/2023 1:47:02 PM
Method	esi_pos_low.m	Operator	Demo User
Sample Name	tpf-220	Instrument	impact II 1825265.10187
Comment			

Acquisition Parameter

Source Type	ESI	Ion Polarity	Positive	Set Nebulizer	0.3 Bar
Focus	Not active	Set Capillary	4500 V	Set Dry Heater	200 °C
Scan Begin	50 m/z	Set End Plate Offset	-500 V	Set Dry Gas	3.0 l/min
Scan End	700 m/z	Set Charging Voltage	2000 V	Set Divert Valve	Source
		Set Corona	0 nA	Set APCI Heater	0 °C

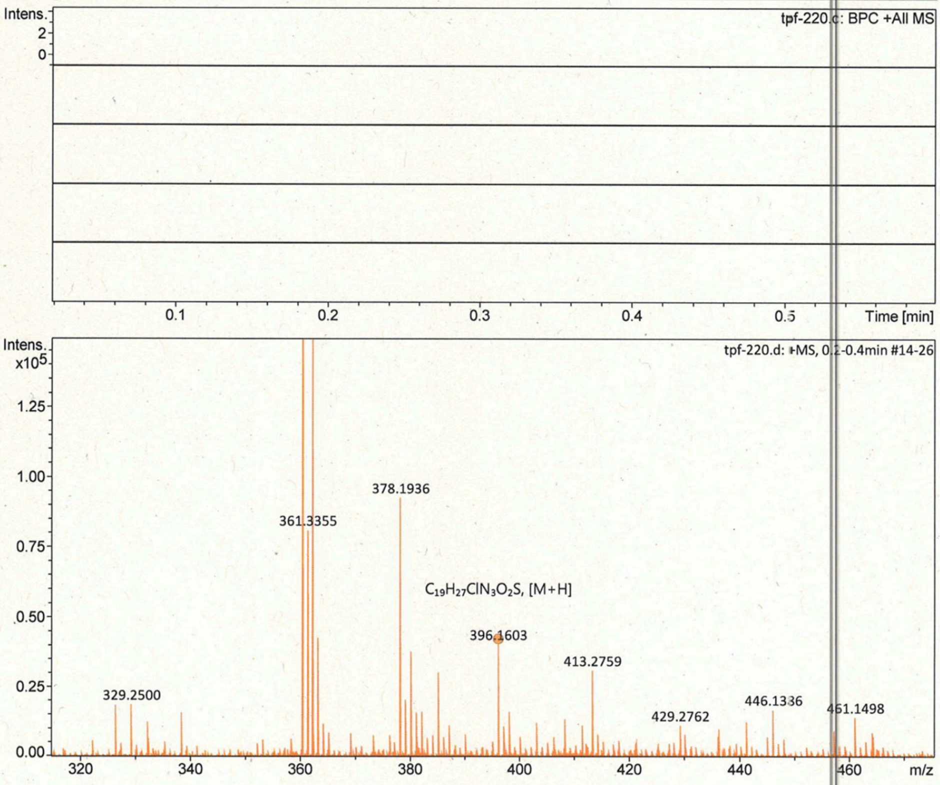

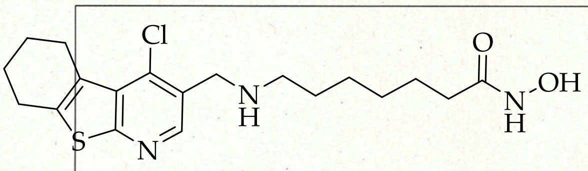

Exact Mass: 395.1434

tpf-220.d
Bruker Compass DataAnalysis 4.4 printed: 3/24/2023 2:20:15 PM by: demo Page 1 of 1

Figure S52. ESI-HRMs of compound **7b**.

Display Report

Analysis Info

Analysis Name	D:\Data\seitai\tateishi\23.3.24\tpf-320.d	Acquisition Date	3/24/2023 1:49:37 PM
Method	esi_pos_low.m	Operator	Demo User
Sample Name	tpf-320	Instrument	impact II 1825265.10187
Comment			

Acquisition Parameter

Source Type	ESI	Ion Polarity	Positive	Set Nebulizer	0.3 Bar
Focus	Not active	Set Capillary	4500 V	Set Dry Heater	200 °C
Scan Begin	50 m/z	Set End Plate Offset	-500 V	Set Dry Gas	3.0 l/min
Scan End	700 m/z	Set Charging Voltage	2000 V	Set Divert Valve	Source
		Set Corona	0 nA	Set APCI Heater	0 °C

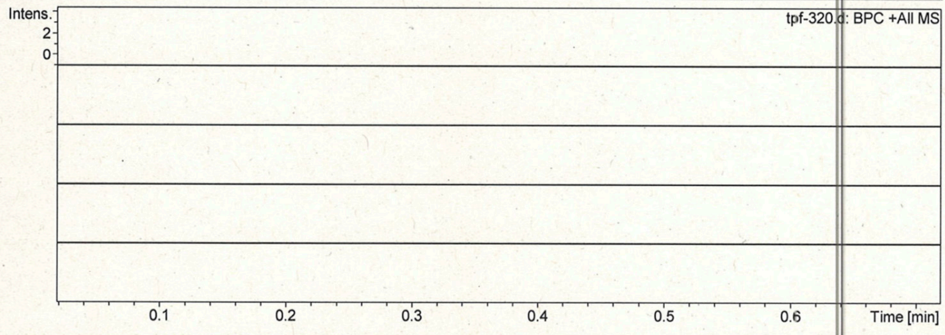

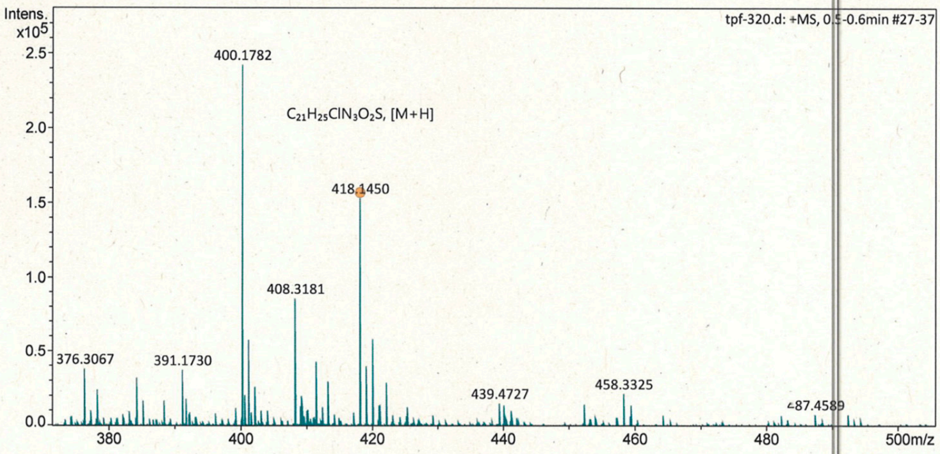

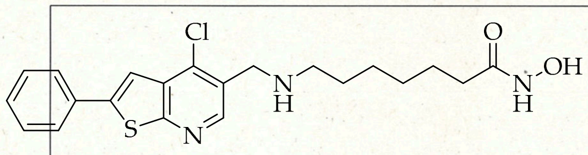

Exact Mass: 417.1278

tpf-320.d
Bruker Compass DataAnalysis 4.4 printed: 3/24/2023 2:22:31 PM by: demo Page 1 of 1

Figure S53. ESI-HRMs of compound **7c**.

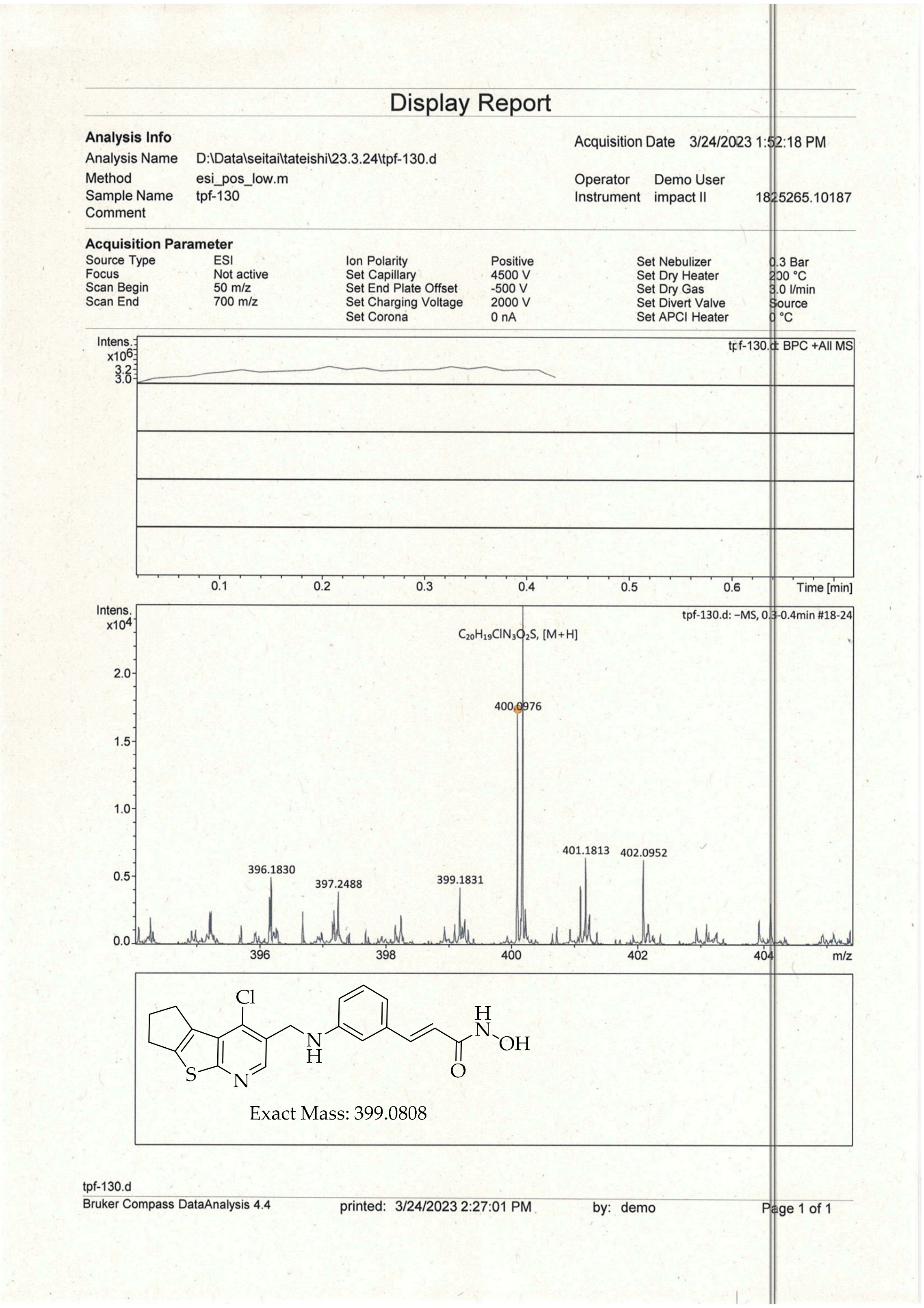

Display Report

Analysis Info

Analysis Name	D:\Data\seitai\tateishi\23.3.24\tpf-130.d	Acquisition Date	3/24/2023 1:52:18 PM
Method	esi_pos_low.m	Operator	Demo User
Sample Name	tpf-130	Instrument	impact II 1825265.10187
Comment			

Acquisition Parameter

Source Type	ESI	Ion Polarity	Positive	Set Nebulizer	0.3 Bar
Focus	Not active	Set Capillary	4500 V	Set Dry Heater	200 °C
Scan Begin	50 m/z	Set End Plate Offset	-500 V	Set Dry Gas	3.0 l/min
Scan End	700 m/z	Set Charging Voltage	2000 V	Set Divert Valve	Source
		Set Corona	0 nA	Set APCI Heater	0 °C

tpf-130.d
Bruker Compass DataAnalysis 4.4 printed: 3/24/2023 2:27:01 PM by: demo Page 1 of 1

Figure S54. ESI-HRMs of compound **9a**.

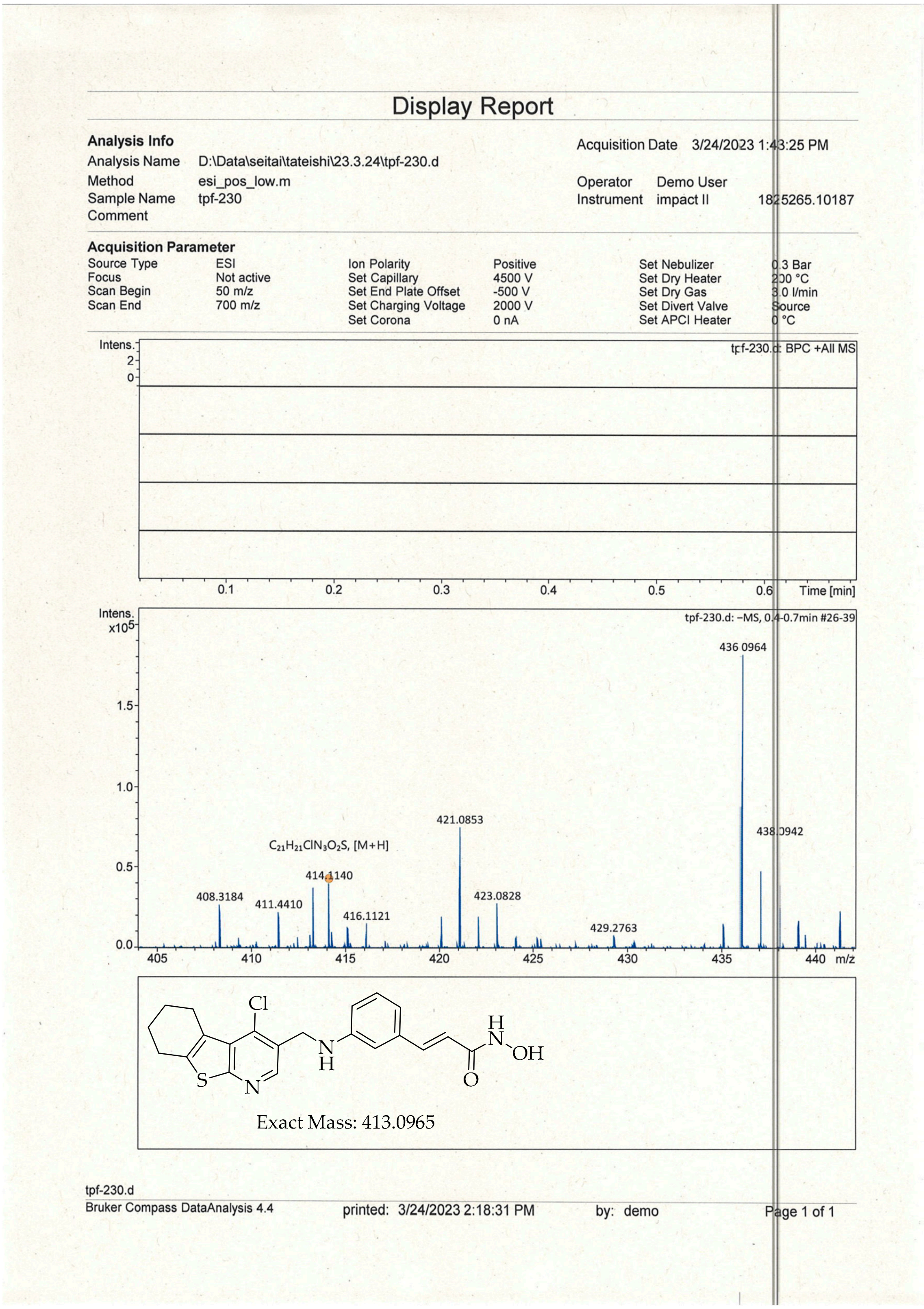
Display Report

Analysis Info

Analysis Name	D:\Data\seitai\tateishi\23.3.24\tpf-230.d	Acquisition Date	3/24/2023 1:43:25 PM
Method	esi_pos_low.m	Operator	Demo User
Sample Name	tpf-230	Instrument	impact II 1825265.10187
Comment			

Acquisition Parameter

Source Type	ESI	Ion Polarity	Positive	Set Nebulizer	0.3 Bar
Focus	Not active	Set Capillary	4500 V	Set Dry Heater	200 °C
Scan Begin	50 m/z	Set End Plate Offset	-500 V	Set Dry Gas	3.0 l/min
Scan End	700 m/z	Set Charging Voltage	2000 V	Set Divert Valve	Source
		Set Corona	0 nA	Set APCI Heater	0 °C

tpf-230.d
Bruker Compass DataAnalysis 4.4 printed: 3/24/2023 2:18:31 PM by: demo Page 1 of 1

Figure S55. ESI-HRMs of compound **9b**.

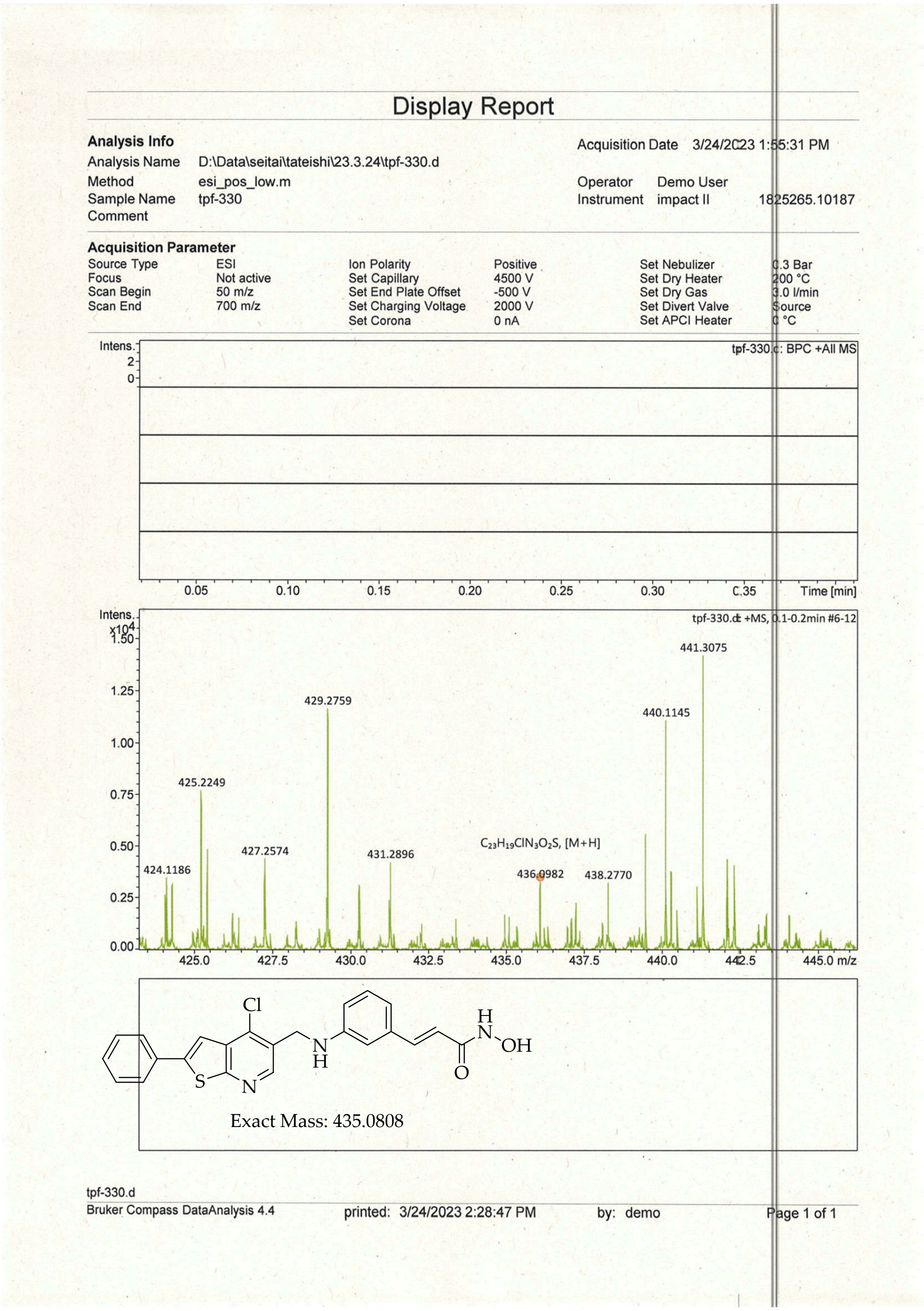

Figure S56. ESI-HRMs of compound **6c**.
